# NCOA4 Accelerates Abdominal Aortic Aneurysm Formation by Regulating Macrophage Activation and Ferroptosis via STAT1–CH25H Axis

**DOI:** 10.1002/advs.78079

**Published:** 2026-09-29

**Authors:** Zhinan Wu, Yuxuan Qian, Wenyi Xu, Fei Liao, Xueliang Liu, Xiao Kuang, Guqing Luo, Xinjie He, Lixian Jiang, Song Ding

**Affiliations:** ^1^ Center Laboratory Punan Branch of Renji Hospital Shanghai Jiao Tong University School of Medicine Shanghai People's Republic of China; ^2^ State Key Laboratory for Innovation and Transformation of Luobing Theory Key Laboratory of Cardiovascular Remodeling and Function Research of MOE NHC CAMS and Shandong Province; Department of Cardiology Qilu Hospital of Shandong University Jinan People's Republic of China; ^3^ National Center for Orthopaedics Shanghai Sixth People's Hospital Affiliated to Shanghai Jiao Tong University School of Medicine Shanghai People's Republic of China; ^4^ Shanghai Key Laboratory of Neuro‐Ultrasound for Diagnosis and Treatment Shanghai Sixth People's Hospital Affiliated to Shanghai Jiao Tong University School of Medicine Shanghai People's Republic of China; ^5^ Department of Cardiology The Second Affiliated Hospital School of Medicine State Key Laboratory of Transvascular Implantation Devices Heart Regeneration and Repair Key Laboratory of Zhejiang province Zhejiang University Hangzhou People's Republic of China; ^6^ Department of Cardiology Shanghai Institute of Cardiovascular Diseases Zhongshan Hospital Fudan University Shanghai People's Republic of China; ^7^ Department of Ultrasound in Medicine Shanghai Sixth People's Hospital Affiliated to Shanghai Jiao Tong University School of Medicine Shanghai People's Republic of China

**Keywords:** 25‐HC, abdominal aortic aneurysm, CH25H, LNP/MBs, macrophage, NCOA4

## Abstract

Abdominal aortic aneurysm (AAA) is a life‐threatening condition lacking effective pharmacological treatments, with emerging evidence highlighting the fundamental role of ferroptosis in its progression. Nuclear receptor coactivator 4 (NCOA4), known to mediate ferritinophagy and contribute to ferroptosis, is upregulated in macrophages from both angiotensin II (Ang II)‐induced murine AAA models and human AAA tissues. Global deletion of *Ncoa4* in mice suppressed Ang II‐induced AAA formation. Combined approaches using bone marrow transplantation and macrophage‐specific adeno‐associated virus (AAV) confirmed that *Ncoa4* knockdown in macrophages alleviates extracellular matrix (ECM) degeneration, inflammation, and vascular damage. Mechanistically, NCOA4 interacts with signal transducer and activator of transcription 1 (STAT1) to co‐activate the transcription of cholesterol 25‐hydroxylase (CH25H), an enzyme that catalyzes the production of 25‐hydroxycholesterol (25‐HC). Elevated 25‐HC levels were detected in the serum of both Ang II‐induced AAA mice and human AAA patients, and they promoted macrophage activation and ferroptosis, thereby driving AAA progression. Importantly, knockdown of *Ch25h* using lipid nanoparticle‐loaded microbubbles (LNP/MBs) attenuated macrophage ferroptosis and AAA progression. These findings reveal a critical role of the NCOA4/STAT1/CH25H axis in AAA pathogenesis and offer promising biomarkers and therapeutic strategies for the disease.

## Graphical abstract

Under pathological stimulation, NCOA4 in macrophages interacts with STAT1 to promote CH25H transcription. The subsequent increase in 25‐HC production activates macrophages, triggers inflammatory responses and ferroptosis, and accelerates the progression of AAA. Intravenous injection of SI‐*Ch25h*‐LNP/MB combined with localized ultrasound bursts can suppress CH25H expression in macrophages within the abdominal aortic wall, thereby offering a therapeutic strategy for AAA.

## 1 Introduction

Abdominal aortic aneurysm (AAA) is a life‐threatening vascular disease characterized by progressive focal weakening and pathological dilation of the abdominal aortic wall [1]. Despite its clinical significance, the precise etiology and pathophysiology of AAA remain poorly understood [2]. Most AAAs progress asymptomatically until rupture, making early diagnosis and prevention difficult [3]. The rising morbidity further exacerbates its substantial healthcare burden worldwide. Current treatment options are limited to surgical interventions, including open repair or endovascular aneurysm repair (EVAR), which carry significant risks and are often unsuitable for small or moderate aneurysms [4]. Given the challenges in timely diagnosis and the lack of effective pharmacological therapies, it is urgent to develop reliable biomarkers and novel therapeutic targets for AAA management.

The pathogenesis of AAA involves a complex interplay of multiple factors, including advanced age, male gender, ethnicity, hypertension, metabolic disorders, platelet dysfunction, and systemic inflammation [5]. Macrophages play a pivotal role in AAA pathogenesis, driving disease progression at every stage [2]. Recruited by chemokines and damage‐associated molecular patterns (DAMPs), circulating monocytes infiltrate the vascular wall, differentiating into macrophages alongside resident populations [6]. Once activated, these macrophages augment vascular inflammation through pro‐inflammatory cytokines (TNF‐α, IL‐6, IL‐1β) and disrupt extracellular matrix (ECM) homeostasis by producing matrix metalloproteinases (MMP‐2, MMP‐9) and suppressing collagen synthesis [7]. Concurrently, excessive reactive oxygen species (ROS) and inflammatory mediators induce endothelial cell and vascular smooth muscle cell (VSMC) damage, promoting ferroptosis and other forms of programmed cell death that accelerate pathological vascular remodeling [8]. Growing preclinical and clinical evidence supports targeting macrophages as a promising therapeutic strategy for AAA, highlighting the importance of further mechanistic studies [9, 10].

NCOA4 (Nuclear Receptor Coactivator 4), also known as ARA70, is a multifunctional protein that has emerged as a key regulator of cellular homeostasis [11]. Numerous studies revealed that NCOA4's role in ferritinophagy involves selectively binding to ferritin heavy chain 1 (FTH1), facilitating ferritin degradation in lysosomes and releasing free iron, thereby maintaining intracellular iron homeostasis [12]. Pathologically, NCOA4‐mediated excessive iron release can lead to elevated ROS production via the Fenton reaction, ultimately inducing cell ferroptosis [13]. Beyond its role in ferritinophagy, NCOA4 exhibits multifaceted biological functions. NCOA4 was initially identified as a transcriptional co‐activator of the androgen receptor (AR), which participates in regulating transcription and DNA replication [14]. Subsequent studies revealed that NCOA4 regulates hematopoiesis through both transcription‐dependent and transcription‐independent mechanisms [15, 16]. Growing evidence highlights the critical involvement of NCOA4 in various cardiovascular diseases and demonstrates the therapeutic potential of targeting the NCOA4‐FTH1 interaction [17, 18]. However, the precise contribution of NCOA4 in AAA remains to be fully elucidated, necessitating further research to evaluate its potential as a therapeutic target for AAA.

In this study, we aimed to elucidate the role of NCOA4 in the pathogenesis of AAA. Our findings demonstrated, for the first time, that global *Ncoa4* knockout or macrophage‐specific *Ncoa4* deficiency attenuated AAA progression. Mechanistically, NCOA4 interacts with signal transducer and activator of transcription 1 (STAT1) to transcriptionally upregulate cholesterol 25‐hydroxylase (CH25H), thereby increasing 25‐Hydroxycholesterol (25‐HC) levels, augmenting inflammatory responses and ferroptosis in macrophages. Besides, targeting CH25H via lipid nanoparticle‐loaded microbubbles (LNP/MBs) in vivo significantly attenuated AAA progression. In summary, our study delineates a novel NCOA4/STAT1/CH25H axis in macrophages that drives AAA pathogenesis. Furthermore, our results identify 25‐HC as an emerging biomarker and suggest potential therapeutic approaches, which collectively could benefit future AAA diagnosis and management. (Scheme 1).

**SCHEME 1:**
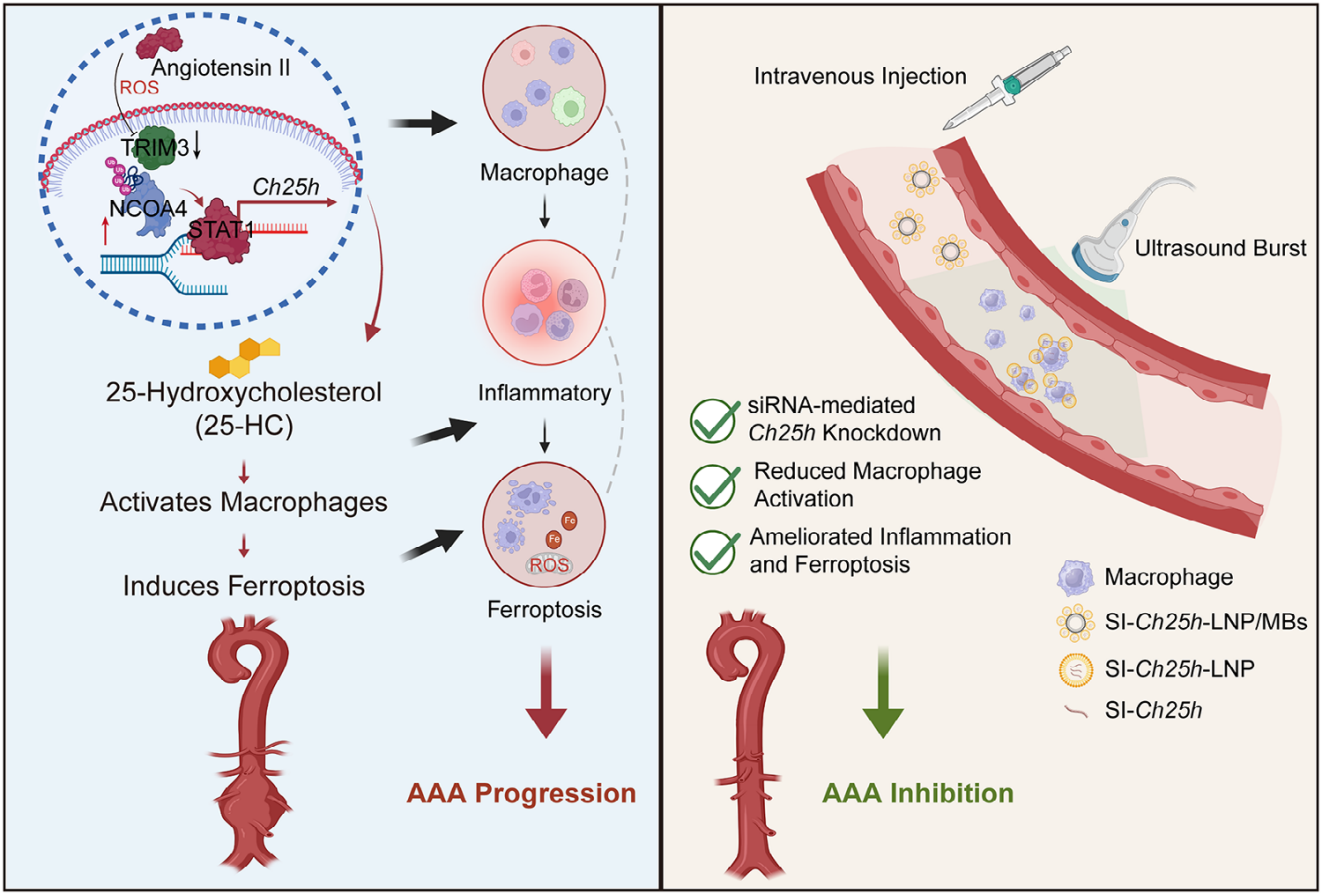
Schematic representation of SI‐*Ch25h*‐LNP/MB for macrophage regulation in AAA. Under pathological stimulation, such as Ang II, NCOA4 in macrophages interacts with STAT1 to form a transcriptional complex that promotes CH25H transcription. The subsequent increase in 25‐HC production by CH25H activates macrophages, triggers inflammatory responses and ferroptosis, and accelerates the progression of AAA. Treatment with intravenous injection of SI‐*Ch25h*‐LNP/MB combined with localized ultrasound bursts can suppress CH25H expression in macrophages within the abdominal aortic wall, thereby offering a therapeutic strategy for AAA. 25‐HC, 25‐hydroxycholesterol; AAA, abdominal aortic aneurysm; Ang II, angiotensin II; CH25H, cholesterol 25‐hydroxylase; LNP/MB, lipid nanoparticle loaded microbubble; NCOA4, nuclear receptor coactivator 4; STAT1, signal transducer and activator of transcription 1. Created in BioRender. P, X. (2026) https://BioRender.com/4q3b4g4.

## 2 Results

### 2.1 Macrophage‐Derived NCOA4 is Upregulated in AAA

To investigate the potential role of NCOA4 in AAA progression, we first analyzed its expression pattern in human AAA tissues and non‐aneurysmal control aortas. The individuals' baseline characteristics are summarized in Table S1. Western blot revealed significant upregulation of NCOA4 protein levels in AAA tissues compared to normal aortic samples (Figure 1A). However, RT‐qPCR analysis showed no significant differences in *NCOA4* mRNA levels between groups (Figure 1B), suggesting post‐transcriptional regulation of NCOA4. This observation was further corroborated in a murine AAA model induced by angiotensin II (Ang II) infusion, where elevated NCOA4 protein expression was detected without corresponding changes in mRNA levels (Figure 1C,D). Immunofluorescence (IF) staining localized NCOA4 expression predominantly in CD68^+^ macrophages within the aortic wall (Figure 1E), implicating macrophages as the primary cellular source of NCOA4 during AAA pathogenesis. Consistent with this finding, Ang II‐stimulated primary peritoneal macrophages (PMs) exhibited increased NCOA4 protein level without alterations in *Ncoa4* at the transcript level (Figure 1F–H), further supporting post‐transcriptional regulation of NCOA4 in AAA development.

**FIGURE 1:**
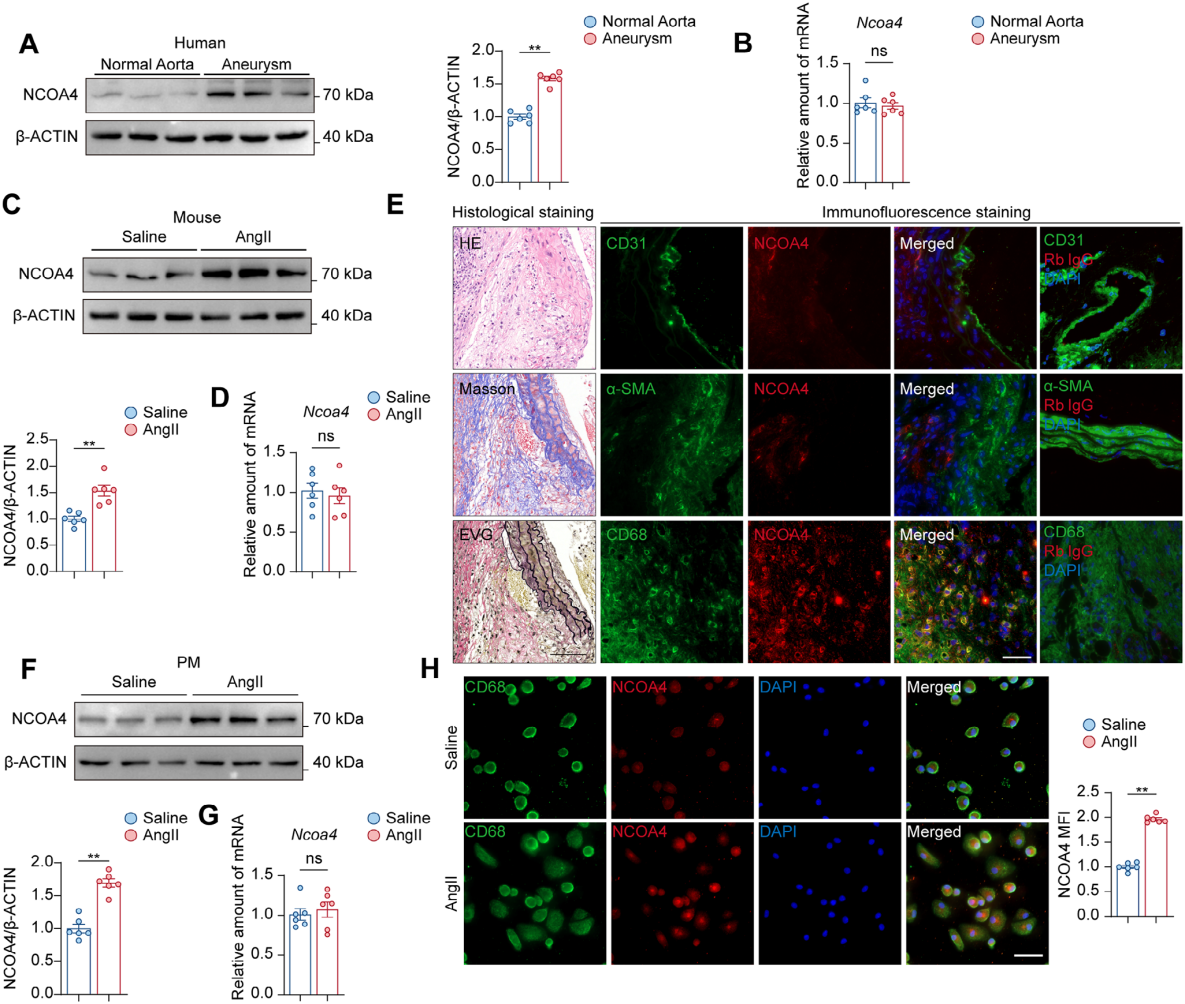
NCOA4 is Upregulated in Human and Mouse AAA. (A) Relative protein levels of NCOA4 in human aortic tissues (n = 6 per group). (B) Relative mRNA levels of *NCOA4* in human aortic tissues (n = 6 per group). (C) Relative protein levels of NCOA4 in mouse aortic tissues (n = 6 per group). (D) Relative mRNA levels of *Ncoa4* in mouse aortic tissues (n = 6 per group). (E) Representative images of histological staining and IF staining of aortic sections (CD68 represents macrophages, CD31 represents endothelial cells, α‐SMA represents vascular smooth muscle, n = 6 per group). Rb IgG was used as the negative control for NCOA4. The scale bar indicates 50 µm. (F) Relative protein levels of NCOA4 in PMs (n = 6 per group). (G) Relative mRNA levels of *Ncoa4* in PMs (n = 6 per group). (H) Representative images of IF staining of PMs and the quantification (n = 6 per group). The scale bar indicates 50 µm. Statistical analyses of A, B, C, D, F, G, and H were analyzed by unpaired *t*‐test. Data are expressed as mean ± SEM. ^**^ indicates *p* < 0.01; ns, not significant. Ang II, angiotensin II; CD31, cluster of differentiation 31; CD68, cluster of differentiation 68; DAPI, 4’,6‐Diamidino‐2’‐phenylindole; EVG, elastic Van Gieson staining; HE, hematoxylin‐eosin staining; MFI, mean Fluorescence Intensity; NCOA4, nuclear receptor coactivator 4; PM, peritoneal macrophage; Rb IgG, Rabbit immunoglobulin G.

To elucidate the molecular mechanisms underlying this regulation, we performed immunoprecipitation coupled with mass spectrometry (IP/MS) in PMs (Figure S1A,B). Among the identified interacting partners, we focused on tripartite motif‐containing 3 (TRIM3), a member of the E3 ubiquitin ligase family known to participate in post‐transcriptional regulation [19]. Co‐immunoprecipitation experiments in both PMs and Human embryonic kidney 293T (HEK‐293T) cells confirmed the physical interaction between NCOA4 and TRIM3 (Figure S1C,D). Notably, TRIM3 co‐expression increased NCOA4 ubiquitination and down‐regulated NCOA4 protein expression in HEK‐293T cells (Figure S1E,F). Further investigation revealed significant downregulation of TRIM3 in both human and murine AAA tissues (Figure S2A–D). In vitro studies demonstrated that Ang II markedly decreased TRIM3 expression in PMs (Figure S2E–G), while treatment with N‐acetylcysteine (NAC), a ROS scavenger, effectively prevented Ang II‐induced TRIM3 downregulation (Figure S2H,I). These findings collectively suggest that Ang II promotes ROS‐mediated TRIM3 suppression, leading to decreased NCOA4 ubiquitination and subsequent protein accumulation in macrophages during AAA progression.

### 2.2 Global *Ncoa4* Ablation Attenuated AAA Progression

Global *Ncoa4* knockout mice were generated to investigate the functional role of NCOA4 in AAA pathogenesis. (Figure S3A). The successful knockout of NCOA4 was verified using PMs from *Ncoa4* ^+/+^ mice and *Ncoa4* ^−/−^ mice (Figure S3B,C). Given NCOA4's established role in ferritinophagy and ferroptosis [20], we first examined key markers and found that NCOA4 deficiency significantly upregulated ferritin heavy chain 1 (FTH1) protein levels, while glutathione peroxidase 4 (GPX4) expression only showed an increasing trend (Figure S4). Using an established Ang II‐induced AAA model (Figure 2), we first confirmed that NCOA4 deficiency did not affect blood pressure and serum lipid profiles (cholesterol, triglycerides, HDL‐C, and LDL‐C) (Figure S5A,B). Of interest, *Ncoa4* knockout significantly improved the survival rates of mice infused with Ang II (Figure 2C). The AAA incidence and the mean maximal abdominal aortic diameter were both down‐regulated in *Ncoa4* ^−/−^ mice compared to littermate controls (Figure 2D,E). Histopathological analysis revealed that *Ncoa4* knockout significantly ameliorated AAA progression; Hematoxylin‐eosin (HE) staining, Masson's trichrome staining, and elastic Van Gieson (EVG) staining all demonstrated significantly reduced aortic remodeling in *Ncoa4* ^−/−^ mice, with reduced elastin degradation grade and maintained collagen content (Figure 2F–H). At the molecular level, NCOA4 deficiency attenuated the inflammatory response induced by Ang II, as evidenced by downregulation of pro‐inflammatory cytokines (*Il1b*, *Il6*, *Tnf*, and *Ptgs2*) in aortic tissues (Figure S6A). The infiltrated macrophages within the aortic wall in the *Ncoa4* ^−/−^ group also decreased (Figure S6B). Furthermore, Western blot analysis showed reduced expression of matrix metalloproteinases (MMP2 and MMP9) in *Ncoa4* ^−/−^ mice (Figure S6C), consistent with the observed reduced ECM degradation after *Ncoa4* knockout. These findings collectively demonstrate that genetic ablation of *Ncoa4* protects against Ang II‐induced aortic remodeling and blunts AAA progression.

**FIGURE 2:**
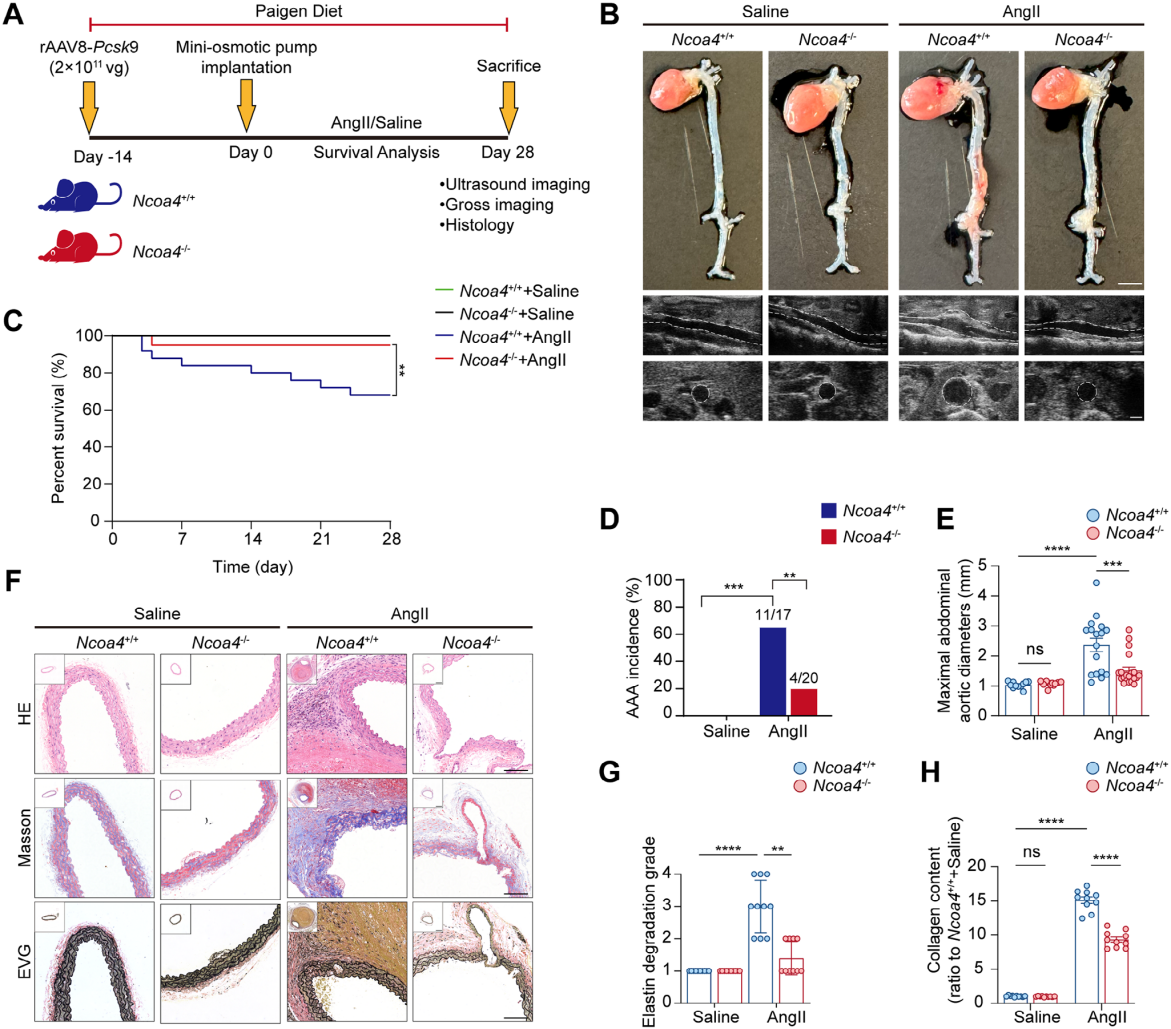
Global *Ncoa4* Ablation Attenuated AAA Progression. (A) Diagram of experimental procedure: *Ncoa4* ^−/−^ mice and *Ncoa4* ^+/+^ mice were injected with rAAV8/D377Y‐mPCSK9 and then subjected to mini‐osmotic pump implantation infused with Ang II (1000 ng/kg/min) for 28 days. All mice were fed a Paigen diet during the procedure. (B) Representative macroscopic and echocardiographic images of abdominal aortas in indicated groups. Scale bars indicate 4 mm (macroscopic image) and 1 mm (echocardiographic image). (C) Survival curves in indicated groups (n = 21–25 per group). Survival rates were analyzed by the Kaplan–Meier method and compared using log‐rank tests. (D) Incidence of AAA induced by Saline or Ang II in indicated groups (n = 11 per group infused with Saline, n = 17–20 per group infused with Ang II). Data were analyzed by a Fisher exact test. (In the *Ncoa4* ^+/+^+Ang II group, 5 mice died with isolated abdominal aortic rupture; 3 mice died with combined thoracic/abdominal aortic rupture prior to termination; in the *Ncoa4* ^−/−^+Ang II group, 1 mouse died with isolated abdominal aortic rupture). (E) Quantification of the maximal diameter of suprarenal abdominal aortas using a caliper at sacrifice (n = 17–20 per group). Data were expressed as the mean ± SEM and analyzed by two‐way analysis of variance (ANOVA) following Tukey's multiple comparisons. (F) Representative images of HE, Masson, and EVG staining of cross‐sections of abdominal aortas. Scale bar indicates 100 µm. (G) Grade of elastin degradation (n = 10 per group). Data were expressed as median with interquartile range and analyzed by nonparametric Kruskal–Wallis test with Dunn's post hoc test. (H) Quantitative analysis of collagen deposition (n = 10 per group). Data were expressed as the mean ± SEM and analyzed by two‐way ANOVA followed by Tukey's multiple comparisons. ns indicates no significance, ^*^ indicates *p* < 0.05, ^**^ indicates *p* < 0.01, ^***^ indicates *p* < 0.001, ^****^ indicates *p* < 0.0001. AAA, abdominal aortic aneurysm; Ang II, angiotensin II; EVG, elastic Van Gieson staining; HE, hematoxylin‐eosin staining.

### 2.3 Macrophage‐Derived NCOA4 Contributed to AAA Formation

Bone marrow transplantation (BMT) experiments were conducted to further investigate the pathogenic role of NCOA4 in AAA development according to previously published protocols [21]. Wild‐type (*Ncoa4* ^+/+^) mice were irradiated to deplete endogenous bone marrow cells (BMCs) and then randomly divided into two groups receiving BMCs from either *Ncoa4* ^+/+^ (*Ncoa4* ^+/+^→*Ncoa4* ^+/+^) or *Ncoa4* ^−/−^ (*Ncoa4* ^−/−^→*Ncoa4* ^+/+^) mice (Figure 3A). The successful BMT was verified by PCR and Western blot (Figure S7A,B). After a 2‐week recovery period, both groups underwent AAA induction. No differences were observed in blood pressure or serum lipid profiles between groups (Figure 3B,C). Notably, wild‐type mice received *Ncoa4* ^−/−^ BMCs showed significantly attenuated AAA progression (Figure 3D), improved survival rates (Figure 3E), reduced AAA incidence, and smaller maximal aortic diameters (Figure 3F,G). Histopathological analysis, including HE staining, MASSON staining, and EVG staining, revealed preserved elastin integrity, reduced collagen deposition, improved aortic remodeling (Figure 3H–J), and reduced infiltrated macrophages (Figure S7B,C) in these mice, which is consistent with the finding in global knockout mice.

**FIGURE 3:**
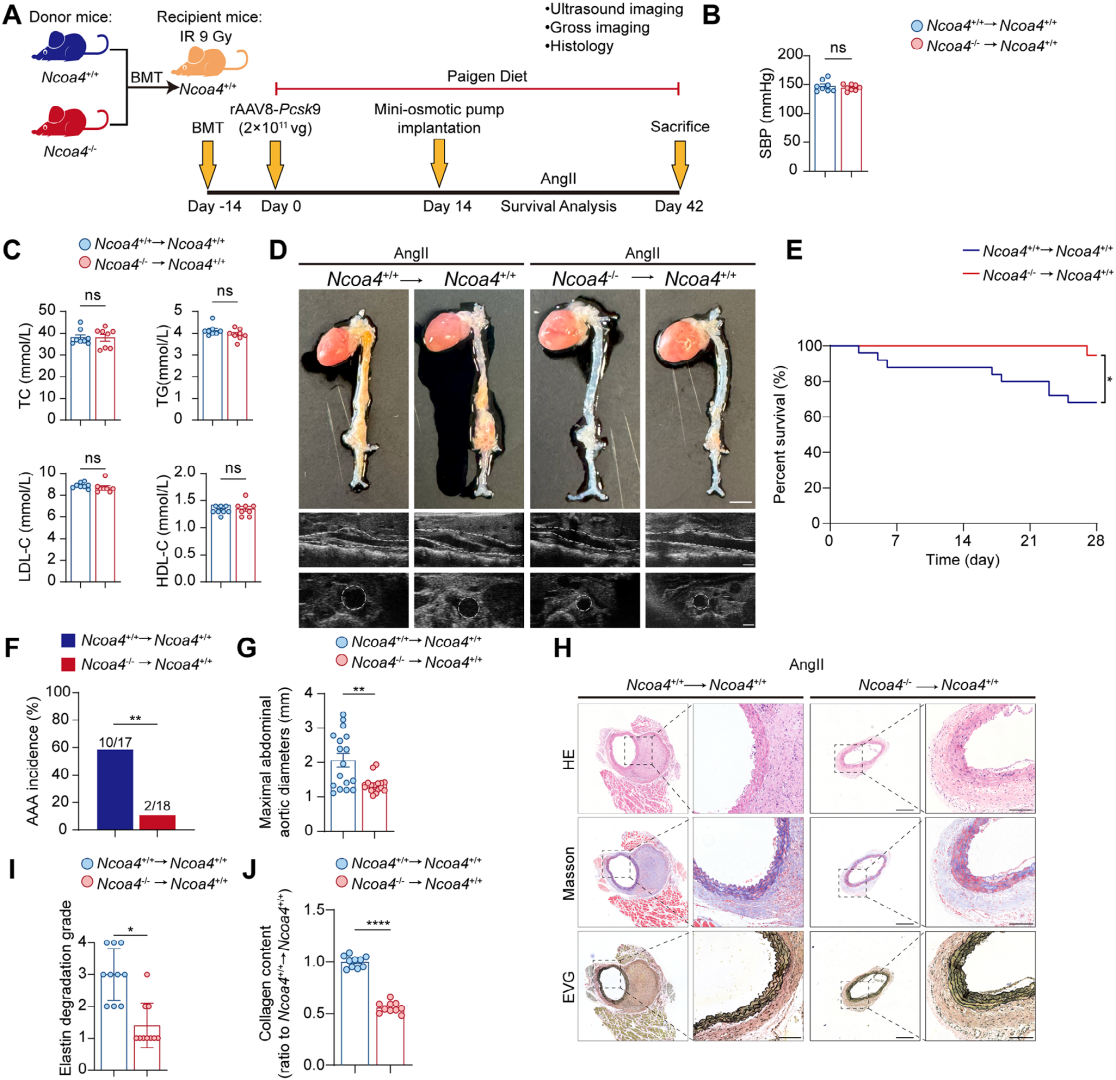
Macrophage‐derived NCOA4 Contributed to AAA Formation. (A) Diagram of experimental procedure: Bone marrow cells from *Ncoa4* ^−/−^ mice or *Ncoa4* ^+/+^ mice were transplanted into recipient *Ncoa4* ^+/+^ mice. After 2 weeks’ recovery, mice were injected with rAAV8/D377Y‐mPCSK9 and then subjected to mini‐osmotic pump implantation infused with Ang II (1000 ng/kg/min) for 28 days. All mice were fed a Paigen diet during the procedure. (B) Blood pressure of *Ncoa4* ^−/−^→*Ncoa4* ^+/+^ mice and *Ncoa4* ^+/+^→*Ncoa4* ^+/+^ mice infused with Ang II (n = 8 per group). (C) Lipid profile of *Ncoa4* ^−/−^→*Ncoa4* ^+/+^ mice and *Ncoa4* ^+/+^→*Ncoa4* ^+/+^ mice infused with Ang II (n = 8 per group). (D) Representative macroscopic and echocardiographic images of abdominal aortas in indicated groups. Scale bars indicate 4 mm (macroscopic image) and 1 mm (echocardiographic image). (E) Survival curves in indicated groups (n = 19–25 for each group). Survival rates were analyzed by the Kaplan–Meier method and compared using log‐rank tests. (In the *Ncoa4* ^+/+^→*Ncoa4* ^+/+^ group, 6 mice died with isolated abdominal aortic rupture; 2 mice died with combined thoracic/abdominal aortic rupture prior to termination; in the *Ncoa4* ^−/−^→*Ncoa4* ^+/+^ group, 1 mouse died with isolated abdominal aortic rupture). (F) Incidence of AAA induced by Ang II in indicated groups (n = 17–18 per group). Data were analyzed by a Fisher exact test. (G) Quantification of the maximal diameter of suprarenal abdominal aortas using a caliper at sacrifice (n = 17–18 per group). (H) Representative images of HE, Masson, and EVG staining of cross‐sections of abdominal aortas. Scale bar indicates 100 µm (left) and 50 µm (right). (I) Grade of elastin degradation (n = 10 per group). Data were expressed as median with interquartile range and analyzed by nonparametric Kruskal–Wallis test with Dunn's post hoc test. (J) Quantitative analysis of collagen deposition (n = 10 per group). Data from B, C, G, and J were expressed as the mean ± SEM and analyzed by unpaired *t*‐test. ns indicates no significance, ^**^ indicates *p* < 0.01, ^***^ indicates *p* < 0.001, ^****^ indicates *p* < 0.0001. AAA, abdominal aortic aneurysm; Ang II, angiotensin II; BMT, bone marrow transplant; EVG, elastic Van Gieson staining; HDL‐C, high‐density lipoprotein cholesterol; HE, hematoxylin‐eosin staining; LDL‐C, low‐density lipoprotein cholesterol; SBP, systolic blood pressure; TC, total cholesterol; TG, triglyceride.

A reciprocal transplantation experiment was performed to confirm the specific contribution of macrophage‐derived NCOA4 to AAA formation (Figure S8A). Wild‐type (*Ncoa4* ^+/+^) BMCs were transplanted into either *Ncoa4* ^+/+^ (*Ncoa4* ^+/+^→*Ncoa4* ^+/+^) or *Ncoa4* ^−/−^ (*Ncoa4* ^+/+^→*Ncoa4* ^−/−^) recipients, with successful engraftment verified (Figure S8B,C). Physiological parameters did not differ significantly between groups (Figure S8D,E). Following AAA induction, both groups showed similar degrees of AAA progression (Figure S8F), comparable survival rates (Figure S8G), identical AAA incidence, and equivalent maximal aortic diameters (Figure S8H,I). Histopathological analysis revealed no differences in aortic remodeling between groups (Figure S8J–L).

To further validate these findings in vivo, we employed a macrophage‐specific adeno‐associated virus (AAV) to knock down NCOA4 (Figure S9A). Mice administered AAV‐Sh*Ncoa4* exhibited a reduced AAA incidence and smaller maximal abdominal aortic diameters compared to the control group injected with AAV‐Sh*Ctrl* (Figure S9B–D). Furthermore, histological analysis revealed attenuated adverse aortic remodeling following AAV‐mediated NCOA4 knockdown (Figure S9E–G). Taken together, these findings strongly suggest that macrophage‐derived NCOA4 plays a critical role in driving AAA progression.

### 2.4 NCOA4/STAT1 Complex Synergistically Modulates Macrophage Activation

To uncover the specific role of NCOA4 in macrophages, we performed RNA‐sequencing on PMs isolated from *Ncoa4* ^+/+^ mice and *Ncoa4* ^−/−^ mice (Figure S10A). Consistent with its transcriptional regulatory function, *Ncoa4* deficiency led to widespread suppression of gene expression, with 631 genes down‐regulated and only 107 genes up‐regulated in *Ncoa4* ^−/−^ PMs compared to *Ncoa4* ^+/+^ PMs (Figure 4A). Bioinformatics analyses revealed that differentially expressed genes (DEGs) were predominantly enriched in immune‐related pathways, including cytokine‐cytokine receptor interaction, antigen processing and presentation, and cell adhesion molecules (Figure 4B), suggesting that NCOA4 plays a critical role in Ang II‐induced macrophage activation and inflammatory responses. In vivo experiments further confirmed that NCOA4 deficiency diminished Ang II‐induced macrophage activation. Key DEGs such as *Arg1*, *Ccr5*, *Irf7*, *Thbs2*, *Ccl5*, and *Gdf3* were all down‐regulated in *Ncoa4* ^−/−^ PMs (Figure 4C), which were all related to macrophage activation and inflammatory response, supporting the conclusion that NCOA4 transcriptionally regulates macrophage activation.

**FIGURE 4:**
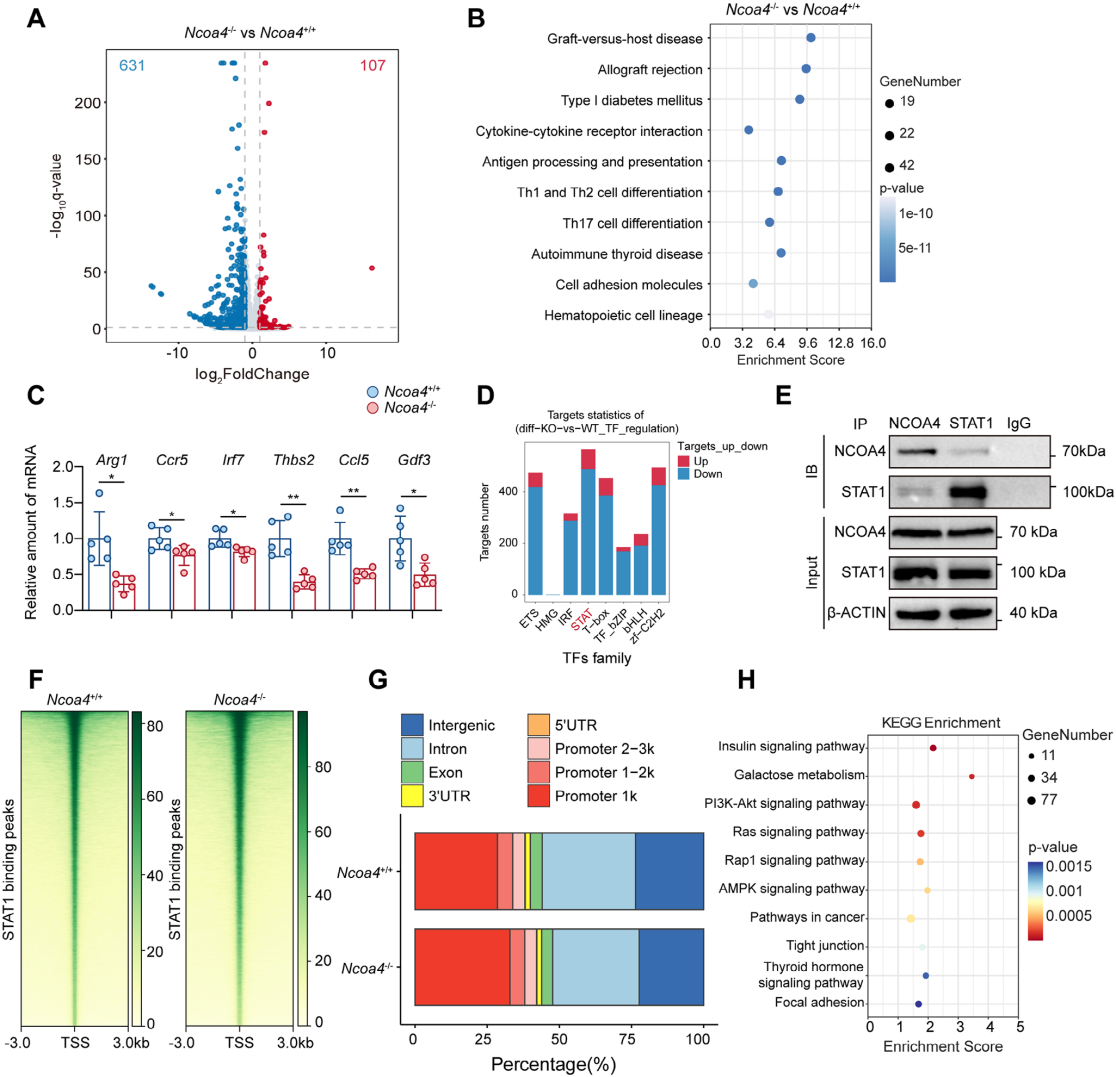
NCOA4/STAT1 Complex Synergistically Modulates Macrophage Activation. (A) Volcano plot revealing the magnitude and significance of DEGs in PMs from *Ncoa4* ^−/−^ mice or *Ncoa4* ^+/+^ mice at 48 h post‐Ang II administration (n = 4 per group). DEGs were defined as genes with a Benjamini‐Hochberg adjusted *P* < 0.05 and |log_2_(foldchange)|≥1. (B) The top 10 enriched KEGG pathways of DEGs in PMs from *Ncoa4* ^−/−^ mice compared to PMs from *Ncoa4* ^+/+^ mice. (C) Relative mRNA expression of indicated genes in PMs from *Ncoa4* ^−/−^ mice or *Ncoa4* ^+/+^ mice at 48 h post‐Ang II administration (n = 6 per group). Data were expressed as the mean ± SEM and analyzed by an unpaired *t*‐test. ^*^ indicates *p* < 0.05, ^**^, *p* < 0.01. (D) Transcription factor prediction using DEGs. (E) Immunoprecipitation results of PMs. (F) Heatmaps showing enrichment of STAT1 CUT & Tag signals in PMs from *Ncoa4* ^−/−^ mice or *Ncoa4* ^+/+^ mice (±3 kb TSS). (G) Annotation of STAT1 binding peaks in PMs from *Ncoa4* ^−/−^ mice or *Ncoa4* ^+/+^ mice. (H) The top 10 enriched KEGG pathways of downregulated STAT1 binding peaks within promoter regions in PMs from *Ncoa4* ^−/−^ mice compared to PMs from *Ncoa4* ^+/+^ mice. CUT & Tag, cleavage under targets and tagmentation; DEGs, differentially expressed genes; IgG, immunoglobulin G; IP, immunoprecipitation; KEGG, Kyoto Encyclopedia of Genes and Genomes; NCOA4, nuclear receptor coactivator 4; PM, peritoneal macrophage; STAT1, signal transducer and activator of transcription 1; TSS, transcription start site.

Given NCOA4's role as a transcription co‐activator, we conducted transcription factor predictive analysis on the RNA‐seq data and identified the signal transducer and activator of transcription (STAT) family as potential partners of NCOA4 (Figure 4D). Revisiting our IP/MS data, we found that STAT1 physically interacts with NCOA4. This interaction was validated by co‐immunoprecipitation (Co‐IP) assays in both PMs and HEK‐293T cells overexpressing Flag‐NCOA4 and HA‐STAT1 (Figure 4E and Figure S10B). To further elucidate the mechanism, we employed Cleavage Under Targets and Tagmentation (CUT & Tag) for NCOA4 in PMs from *Ncoa4* ^+/+^ mice and *Ncoa4* ^−/−^ mice (Figure 4F,G). Notably, downregulated STAT1 binding peaks in *Ncoa4* ^−/−^ PMs were enriched in pathways critical for macrophage activation, including PI3K‐Akt, Ras, and Rap1 signaling (Figure 4H). The above results demonstrated that NCOA4 forms a functional complex with STAT1 to cooperatively regulate transcriptional activities essential for macrophage activation.

### 2.5 NCOA4 Transcriptionally Regulates *Ch25h* in a STAT1‐Dependent Manner

We performed integrated analysis of RNA‐seq, CUT & Tag, and online human and mouse AAA datasets to identify direct targets of the NCOA4/STAT1 complex during AAA pathogenesis (Figure 5A). Among the three overlapping candidate genes (*Ch25h*, *Nfatc4*, and *Ciita*), *Ch25h* exhibited the most pronounced downregulation in *Ncoa4* ^−/−^ PMs (Figure 5B). Integrative Genomics Viewer (IGV) analysis revealed significantly reduced STAT1 binding at the *Ch25h* promoter in *Ncoa4* ^−/−^ PMs, suggesting suppressed *Ch25h* transcription (Figure 5C). The putative binding site of the NCOA4/STAT1 complex at the promoter of the mouse *Ch25h* locus was predicted by JASPAR [22], which was confirmed by chromatin immunoprecipitation‐quantitative polymerase chain reaction (ChIP‐qPCR) in PMs (Figure 5D). Dual‐luciferase reporter assays further demonstrated that NCOA4 and STAT1 synergistically enhanced transcription of *Ch25h*, an effect abolished upon mutation of the binding site (Figure 5E). Given that this transcriptional activity is mediated by STAT1, we investigated the effect of NCOA4 deficiency on STAT1 expression and activation. Of interest, NCOA4 deficiency in macrophages did not alter total STAT1 expression but reduced STAT1 phosphorylation at Serine 727 (p‐STAT1‐S727), indicating suppressed STAT1‐mediated transcriptional activity (Figure S11). Consistent with these findings, *Ncoa4* deficiency led to a marked reduction in CH25H protein levels, as confirmed by Western blot and IF staining (Figure 5F,G). We also verified our conclusions in Bone Marrow‐Derived Macrophages (BMDMs) and observed the identical results (Figure S12). Collectively, these results confirm *Ch25h* is a key transcriptional target of the NCOA4/STAT1 complex in macrophages during AAA development.

**FIGURE 5:**
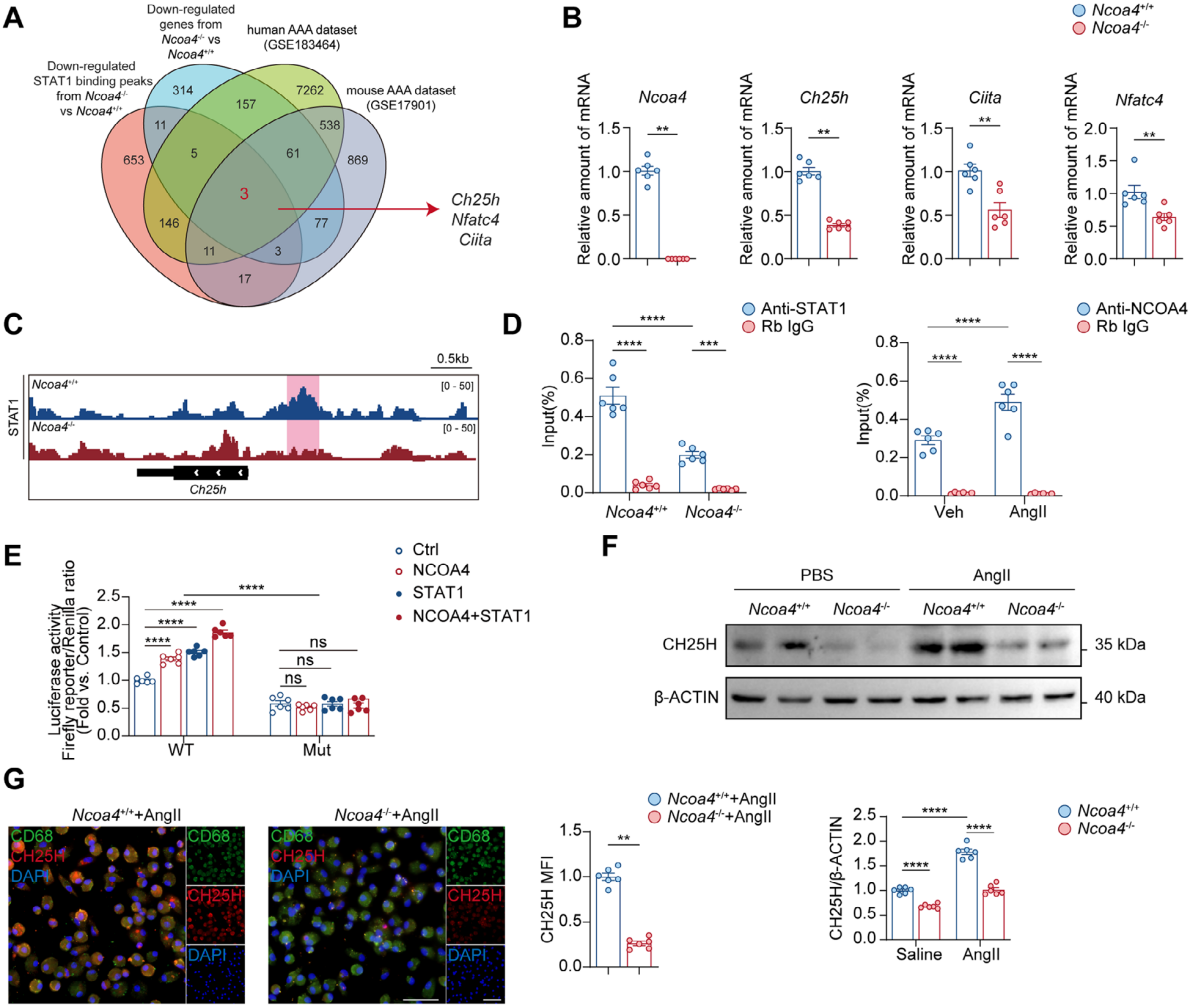
NCOA4 Transcriptionally Regulates *Ch25h* in a STAT1‐Dependent Manner. (A) Venn diagram showing the overlap of down‐regulated STAT1 binding peaks and down‐regulated genes from PMs from *Ncoa4* ^−/−^ mice or *Ncoa4* ^+/+^ mice and DEGs from human AAA dataset (GSE183464) and mouse AAA dataset (GSE17901). (B) Relative mRNA expression of indicated genes in PMs from *Ncoa4* ^−/−^ mice or *Ncoa4* ^+/+^ mice at 48 h post‐Ang II administration (n = 6 per group). Data were expressed as the mean ± SEM and analyzed by unpaired *t*‐test. (C) IGV view of the *Ch25h* gene showing sequencing reads from STAT1 CUT & Tag analyses. The promoter regions are highlighted in red. (D) ChIP‐qPCR was performed for STAT1 or NCOA4 and IgG occupancy at *Ch25h* promoter fragments in indicated groups (n = 6 per group). Data were analyzed by two‐way ANOVA following Tukey's multiple comparisons. (E) *Ch25h* transcriptional activities were analyzed in HEK293T cells co‐transfected with STAT1 and NCOA4 overexpression plasmids and a luciferase reporter driven by the indicated promoter. pRL‐TK‐renilla plasmid was used as an internal control for transfection efficiency (n = 6 per group). Data were expressed as mean ± SEM and analyzed by one‐way ANOVA following Tukey's multiple comparisons. (F) Western blot of PMs and the quantification (n = 6 per group). Data were analyzed by two‐way ANOVA following Tukey's multiple comparisons. (G) Representative images of IF staining of PMs and the quantification (n = 6 per group). Scale bar indicates 50 µm. Data were expressed as the mean ± SEM and analyzed by unpaired *t*‐test. ns indicates no significance; ^**^, *p* < 0.01; ^***^, *p* < 0.001; ^****^, *p* < 0.0001. Ang II, angiotensin II; CH25H, cholesterol 25‐hydroxylase; DEGs, differentially expressed genes; IgG, immunoglobulin G; PM, peritoneal macrophage; STAT1, signal transducer and activator of transcription 1.

### 2.6 CH25H/25‐HC Contributes to AAA Progression via Augmenting Macrophage Inflammation and Ferroptosis

Our examination of CH25H variation during AAA modeling revealed that Ang II infusion significantly increased CH25H expression at both protein and mRNA levels, an effect abolished by *Ncoa4* knockout (Figure 6A,B). IF staining further confirmed elevated CH25H expression in macrophages within AAA lesions (Figure 6C). Notably, serum levels of 25‐hydroxycholesterol (25‐HC), the enzymatic product of CH25H [23], were markedly increased in Ang II‐infused mice (Figure 6D). Consistent with this, CH25H expression is also up‐regulated in peripheral blood mononuclear cells (PBMCs) from those mice (Figure 6E,F). These findings were corroborated in human samples. Compared with controls, AAA patients exhibited significantly elevated CH25H expression in both aneurysmal aortic tissues and PBMCs, accompanied by increased serum 25‐HC levels (Figure 6G–K). The baseline demographic and clinical characteristics of the participants enrolled in the PBMC study are summarized in Table S2. The above results suggest that macrophage‐derived CH25H contributes to AAA progression via 25‐HC production.

**FIGURE 6:**
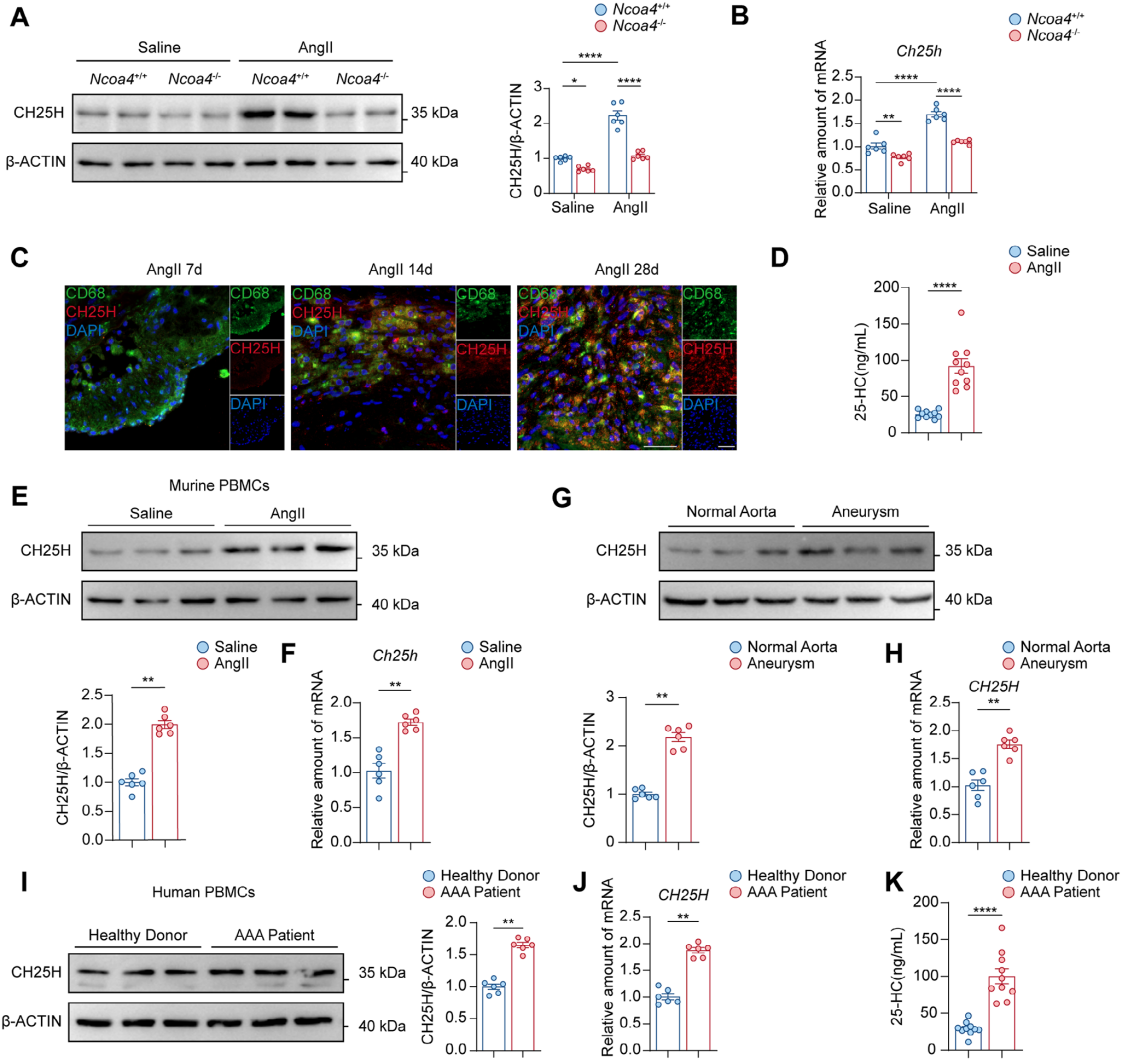
CH25H/25‐HC Contributes to AAA Progression via Augmenting Macrophage Inflammation and Ferroptosis. (A) Relative protein level of CH25H in mouse aortic tissues (n = 6 per group). (B) Relative mRNA level of *Ch25h* in mouse aortic tissues (n = 6 per group). (C) Representative images of IF staining of aortic sections (n = 6 per group). Scale bar indicates 50 µm. (D) 25‐HC levels in serum from mice infused with Saline or Ang II (n = 10 per group). (E) Relative protein levels of CH25H in mouse PBMCs (n = 6 per group). (F) Relative mRNA levels of *Ch25h* in mouse PBMCs (n = 6 per group). (G) Relative protein levels of CH25H in human aortic tissues (n = 6 per group). (H) Relative mRNA levels of *CH25H* in human aortic tissues (n = 6 per group). (I) Relative protein levels of CH25H in human PBMCs (n = 6 per group). (J) Relative mRNA levels of *CH25H* in human PBMCs (n = 6 per group). (K) 25‐HC levels in serum from AAA patients or healthy donors (n = 10 per group). Data from A and B were expressed as mean ± SEM and analyzed by two‐way ANOVA following Tukey's multiple comparisons. Data from D, E, F, G, H, I, J, and K were expressed as the mean ± SEM and analyzed by unpaired *t*‐test. ^**^ indicates *p* < 0.01; ^***^, *p* < 0.001; ^****^, *p* < 0.0001. 25‐HC, 25‐Hydroxycholesterol; AAA, abdominal aortic aneurysm; Ang II, angiotensin II; CH25H, cholesterol 25‐hydroxylase; PM, peritoneal macrophage.

In vitro experiments revealed that Ang II stimulation increased 25‐HC levels in culture medium, an effect abolished by *Ch25h* knockdown (Figure S13A). *Ch25h* knockdown also attenuated Ang II‐induced macrophage activation, as evidenced by reduced expression of pro‐inflammatory factors (*Il1b*, *Il6*, *Tnf*, and *Ptgs2*; Figure S13B). Given recent reports implicating 25‐HC in ferroptosis induction [24], we confirmed that *Ch25h* knockdown preserved expression of ferroptosis inhibitors, including NRF2, SLC7A11, and GPX4, and reduced iron accumulation, ROS production, and lipid peroxidation (Figure S14A,B). Besides, mitochondrial dysfunction and lipid peroxidation were also mitigated, as shown by decreased signals of Mitotracker Red, FerroOrange, and MitoPeDPP staining (Figure S14C).

To further verify the NCOA4/STAT1/CH25H axis in macrophages, we overexpressed NCOA4 in macrophages while simultaneously knocking down *Stat1* expression via siRNA transfection. As anticipated, *Stat1* knockdown significantly abrogated NCOA4‐driven CH25H expression. Concurrently, the associated macrophage phenotypes—specifically macrophage ferroptosis—were effectively reversed (Figure S15A). Furthermore, we overexpressed CH25H in *Ncoa4*‐knockout macrophages. Importantly, CH25H overexpression successfully compensated for NCOA4 deficiency, recapitulating the ferroptotic phenotypes that were initially suppressed by *Ncoa4* loss (Figure S15B). To further validate this relationship in vivo, we employed AAV‐mediated *Ncoa4* and *Stat1* knockdown. Aligning with our in vitro findings, depleting STAT1 robustly blunted AAA progression in the control group. Conversely, *Stat1* knockdown yielded no such protective effect in mice with NCOA4, confirming that the pro‐aneurysmal role of STAT1 depends on the presence of NCOA4 (Figure S16). These findings establish a mechanistic link between NCOA4/STAT1‐mediated CH25H up‐regulation, 25‐HC‐induced macrophage activation and ferroptosis, and ultimately AAA progression.

### 2.7 Spatiotemporal Targeting CH25H Attenuates AAA progression In Vivo

To overcome the challenge of targeting macrophages within abdominal aortic tissues under hemodynamic conditions, we developed an acoustically responsive delivery system based on microbubbles (MBs) for SI‐*Ch25h* delivery. Initially, lipid nanoparticles (LNPs) carrying SI‐*Ch25h* were synthesized using the microfluidic mixing method and connected to SonoVue MBs, an FDA‐approved clinical ultrasound contrast agent, via a Michael addition reaction (Figure S17). Bright‐field imaging using an all‐in‐one fluorescence microscope showed that SI‐*Ch25h*‐LNP/MB retained a uniform spherical morphology similar to unconjugated MB (Figure S18A). Hydrodynamic size measured by Particle Sizing Systems (PSS) revealed values of approximately 2.05 µm for bare MB and approximately 2.22 µm for SI‐*Ch25h*‐LNP/MB, respectively (Figure S18B). Zeta potential measurements, coupled with dynamic light scattering (DLS), further indicated a zeta potential of 47.79 ± 0.98 mV and a particle size of 303±112 nm for SI‐*Ch25h*‐LNP; −57.77 ± 10.39 mV and 1431 ± 315 nm for bare MB; and −29.92 ± 4.36 mV and approximately 1416 ± 416 nm for SI‐*Ch25h*‐LNP/MB (Figure S18C,D). Collectively, these results demonstrate that surface binding with SI‐*Ch25h*‐LNP preserved the morphological characteristics of MB but modified their surface charge and slightly changed the particle size.

Utilizing an in vitro hemodynamic model, we successfully confirmed the ability of the LNP/MB system to penetrate into the microfluidic hydrogel tube wall with ultrasound destruction (Figure S18E). To evaluate the effect of SI‐*Ch25h*‐LNP/MB in vivo, we utilized a clinically used ultrasound system, the Mindray Resona R9 Pro (Microbubble Destruction Mode), leveraging its capacity to induce MB cavitation and facilitate local delivery to the aortic wall. Mice were randomly assigned to four groups after mini‐osmotic pump implantation. SI‐NC, SI‐*Ch25h*, SI‐*Ch25h*‐LNP, or SI‐*Ch25h*‐LNP/MB were intravenously administered on Day 7 after mini‐osmotic pump implantation. Following injection, real‐time imaging of the abdominal aorta was performed with Dual Live mode, and several seconds of Microbubble Destruction mode were performed to destroy MBs and deliver the SI‐*Ch25h*‐LNP into the abdominal aortic tissue via microstreaming and microjets caused by MB destruction (Figure S18F). At 21 days post‐treatment, the abdominal aortic tissues from each group were harvested, and IF staining was performed, revealing that abdominal aortic tissues treated with SI‐*Ch25h*‐LNP/MB had significantly reduced CH25H expression with aneurysm (Figure S18G). Besides, we have also thoroughly evaluated the systemic biodistribution of this approach (Figure S20). Taken together, these findings confirm the successful development of an effective system for lesion‐targeted knockdown of CH25H in macrophages.

After confirming the biosafety of SI‐*Ch25h*‐LNP/MB and SI‐*Ch25h*‐LNP/MB with ultrasound destruction at the level of HE staining, liver and kidney function indices, and complete blood count (Figure S21 and Table S7), we conducted in vivo experiments to further assess the therapeutic efficacy of this strategy (Figure 7A). As expected, lesion‐targeted knockdown of CH25H in vivo significantly attenuated AAA progression and improved survival rates (Figure 7B,C). The incidence of AAA and maximal aortic diameters were markedly reduced following administration of SI‐*Ch25h*‐LNP/MB (Figure 7D,E). The administration did not alter blood pressure or lipid profiles (Figure S19A,B). Histological analysis further demonstrated improved aortic remodeling (Figure 7F–H) and reduced macrophage infiltration (Figure S19C) in the SI‐*Ch25h*‐LNP/MB + Ang II group compared to the SI‐NC‐LNP/MB + Ang II group, corroborating the therapeutic benefits of our approach. The above results indicated the effects of our strategy; we further employed our strategy in mice with advanced aneurysm (Figure S22A) and further verified its therapeutic effects. SI‐*Ch25h*‐LNP/MB administration effectively deterred the progression of AAA compared to the control group (Figure S22B–F).

**FIGURE 7:**
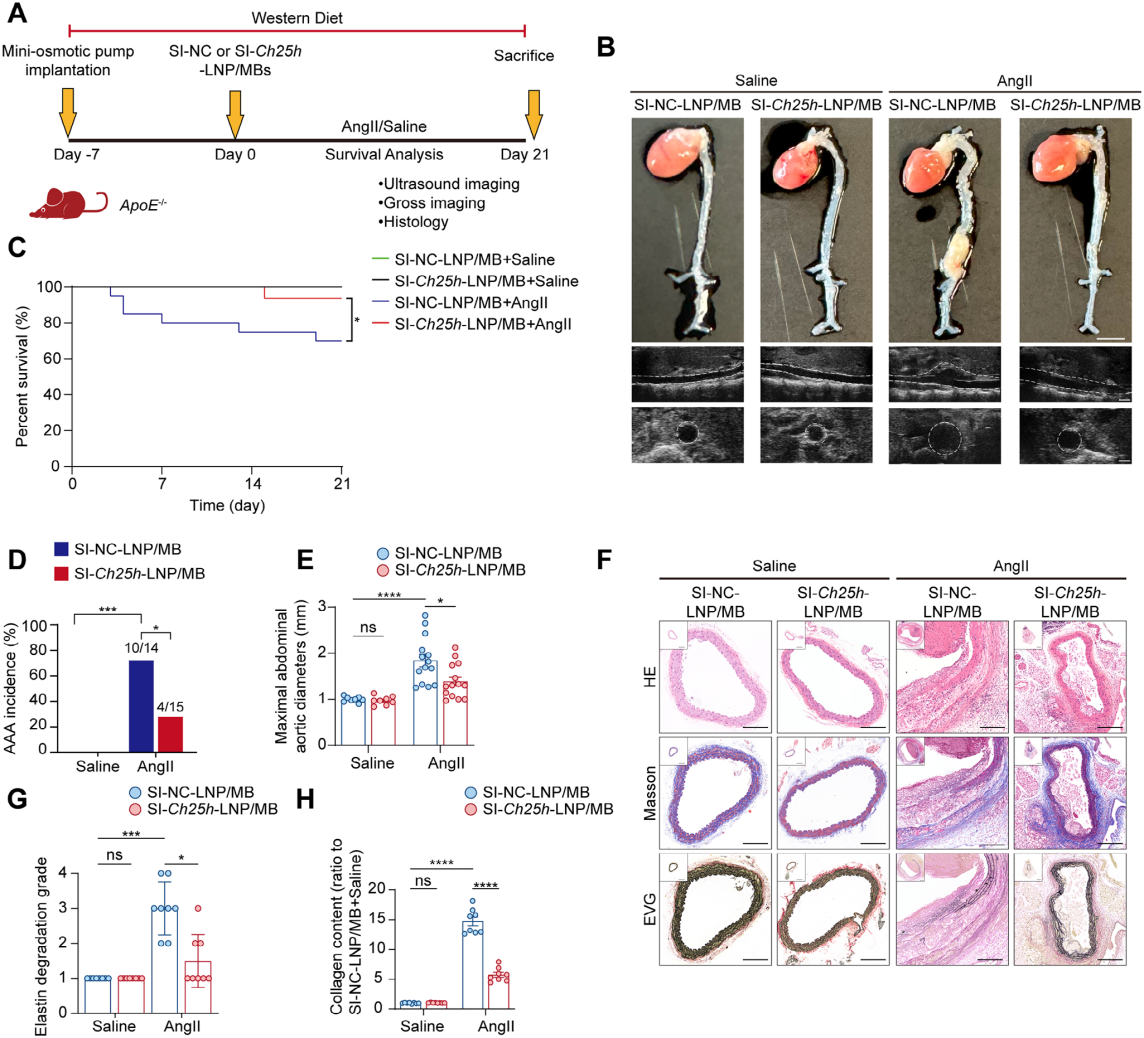
Targeting CH25H via LNP/MBs Attenuates AAA Progression. (A) Diagram of experimental procedure: *ApoE* ^−/−^ mice were randomly divided into 2 groups and subjected to mini‐osmotic pump implantation, infused with Ang II (1000 ng/kg/min) or normal saline. 7 days after operation, the mice infused with Ang II or normal saline were randomly divided into 2 groups again and injected with SI‐NC‐LNP/MB or SI‐*Ch25h*‐LNP/MB. All mice were fed a Western diet during the procedure. (B) Representative macroscopic and echocardiographic images of abdominal aortas in indicated groups. Scale bars indicate 4 mm (macroscopic image) and 1 mm (echocardiographic image). (C) Survival curves in indicated groups (n = 8 per group infused with Saline, n = 16–20 per group infused with Ang II). Survival rates were analyzed by the Kaplan–Meier method and compared using log‐rank tests. (In the SI‐NC‐LNP/MB+Ang II group, 5 mice died with isolated abdominal aortic rupture, and 1 mouse died with combined thoracic/abdominal aortic rupture prior to the termination; in the SI‐*Ch25h*‐LNP/MB group, 4 mice died with isolated abdominal aortic rupture). (D) Incidence of AAA induced by Saline or Ang II in indicated groups (n = 8 per group infused with Saline, n = 14–15 per group infused with Ang II). Data were analyzed by a Fisher exact test. (E) Quantification of the maximal diameter of suprarenal abdominal aortas using a caliper at sacrifice (n = 8 per group infused with Saline, n = 14–15 per group infused with Ang II). Data were expressed as the mean ± SEM and analyzed by two‐way ANOVA following Tukey's multiple comparisons. (F) Representative images of HE, Masson, and EVG staining of cross‐sections of abdominal aortas. Scale bar indicates 100 µm. (G) Grade of elastin degradation (n = 8 per group). Data were expressed as median with interquartile range and analyzed by nonparametric Kruskal–Wallis test with Dunn's post hoc test. (H) Quantitative analysis of collagen deposition (n = 10 per group). Data were expressed as the mean ± SEM and analyzed by two‐way ANOVA followed by Tukey's multiple comparisons. ns indicates no significance, ^*^ indicates *p* < 0.05, ^**^ indicates *p* < 0.01, ^***^ indicates *p* < 0.001, ^****^ indicates *p* < 0.0001. AAA, abdominal aortic aneurysm; Ang II, angiotensin II; EVG, elastic Van Gieson staining; HE, hematoxylin‐eosin staining; LNP/MB, lipid nanoparticle‐loaded microbubble.

## 3 Discussion and Conclusion

In the present work, we elucidate the precise role of NCOA4 in AAA pathogenesis, revealing several critical discoveries: First, macrophage‐derived NCOA4 promotes inflammation, ECM degradation, aortic remodeling, and AAA progression. Second, NCOA4 interacts with STAT1 to transcriptionally upregulate *Ch25h*, thereby elevating 25‐HC levels, inducing macrophage activation, pro‐inflammatory responses, and ferroptosis. Third, we observed elevated 25‐HC levels in serum from both mice and humans with AAA, which could potentially serve as a non‐invasive diagnostic biomarker. Furthermore, targeting CH25H via LNP/MBs in vivo demonstrated therapeutic effects against AAA, suggesting it may hold promise as a future strategy. In general, our study provides novel insights into the pathogenesis of AAA by uncovering a previously unrecognized role of the NCOA4/STAT1/CH25H axis in macrophages. Furthermore, these findings propose a potential biomarker and suggest a novel therapeutic approach that could ultimately contribute to AAA management.

The central role of macrophages in AAA formation and progression has been emphasized by recent studies. At the onset of AAA, classically activated macrophages release pro‐inflammatory cytokines and interact with T cells and mast cells, forming a self‐amplifying feedback loop that sustains chronic inflammation [25]. Targeting the IRF5/PI3Kγ pathway ameliorated AAA by inhibiting macrophage infiltration and migration [26]. Supporting this, Wu et al. demonstrated that *Ninj1* knockout suppressed macrophage activation and inflammatory responses, thereby attenuating AAA progression [27]. During AAA development, ADAR1 or ILF3 exacerbate inflammation via the NF‐κB pathway, disrupting the balance between pro‐ and anti‐inflammatory responses [28, 29]. The suppression of alternatively activated macrophages further compromises vascular repair, leading to an imbalance between tissue damage and regeneration [30]. Concurrently, excessive MMP release accelerates ECM degradation, while complement activation and NETosis intensify oxidative stress [31]. Recent evidence further indicates that elevated ROS and lipid peroxidation drive ferroptosis and lead to AAA rupture, while inhibiting ferroptosis via hydrogen sulfide (H_2_S) supplementation mitigated AAA [32]. Here, our study identified a previously unrecognized NCOA4‐dependent signaling pathway that promotes macrophage activation in AAA, offering novel directions for AAA management.

As a multifunctional protein, NCOA4 exhibits a broad spectrum of biological functions beyond its well‐characterized role in ferritinophagy. For instance, it has been shown to exacerbate sepsis‐induced injury by activating the AGER‐NF‐κB pathway [33]. Notably, one of the most significant aspects of NCOA4 is its involvement in transcriptional regulation. Dysregulation of NCOA4 expression, particularly through chromosomal translocations that fuse NCOA4 with the RET tyrosine kinase gene (resulting in the NCOA4‐RET fusion), is closely associated with the pathogenesis of papillary thyroid carcinoma, highlighting its critical role in transcriptional control [34]. Furthermore, NCOA4 can function as a transcriptional co‐regulator by binding to AR to promote prostate cancer progression [35], or by cooperating with peroxisome proliferator‐activated receptor γ (PPARγ) to regulate transcription in adipocytes [36]. Our study confirmed for the first time that in macrophages, NCOA4 physically interacts with STAT1 to form a complex that synergistically enhances *Ch25h* transcription. Through thorough high‐throughput sequencing and molecular experiments, we conclusively demonstrated that genetic ablation of *Ncoa4* significantly attenuates STAT1‐mediated transcriptional activation of *Ch25h*, suppressing macrophage activation and ferroptosis. Beyond the classical hemorrhage‐driven Fenton reaction and NCOA4‐mediated ferritinophagy, we demonstrate for the first time that NCOA4 also drives ferroptosis through transcriptional regulation. Specifically, our newly identified NCOA4‐STAT1‐CH25H axis transcriptionally amplifies lipid peroxidation. Ultimately, the synergistic interplay between canonical iron metabolism and this novel NCOA4‐dependent transcriptional pathway comprehensively orchestrates macrophage ferroptosis during AAA progression.

A major challenge in dissecting AAA pathogenesis is the intertwined nature of inflammation and ferroptosis. Rather than operating in a linear sequence, our findings, along with emerging evidence, suggest a feed‐forward loop: early inflammatory signals and ROS drive macrophage activation, with ferroptosis arising as a downstream cellular outcome [37, 38]. In turn, ferroptotic cells release Damage‐Associated Molecular Patterns (DAMPs) and cytokines that intensify local inflammation and accelerate tissue degradation [39, 40]. We acknowledge that our current approaches cannot fully separate the effects of ferroptosis from broader anti‐inflammatory actions. Nonetheless, our data strongly support ferroptosis as a critical amplifying mechanism within the macrophage‐driven inflammatory cascade in AAA. These results define a vital, ferritinophagy‐independent role for NCOA4 in inflammation and the initial stage of ferroptosis, emphasizing its fundamental importance in regulating this cell death process.

While NCOA4 has been implicated in various disease processes, it is crucial to recognize its indispensable role in hematopoiesis and erythropoiesis. Research has demonstrated that NCOA4 serves as a critical cofactor for thyroid hormone (TH)‐mediated transcriptional regulation during terminal erythrocyte differentiation, with its deficiency resulting in TH signaling impairment and consequent anemia [41]. Moreover, as a central regulator of iron metabolism and homeostasis, NCOA4 orchestrates murine erythropoiesis through both cell‐autonomous and non‐autonomous mechanisms [15, 16]. Considering the inherent risks of hemorrhage associated with AAA rupture and surgical intervention, we conclude that systemic targeting of NCOA4 is not the best choice for AAA management.

CH25H plays a pivotal role in modulating immune responses and lipid metabolism by catalyzing cholesterol oxidation into 25‐HC [42]. Extensive research has demonstrated that CH25H regulates glial cell activation, enhances the secretion of inflammatory factors and chemokines, and exacerbates neuroinflammation, thereby accelerating neurodegeneration [43, 44, 45, 46]. Under LPS stimulation, CH25H expression is up‐regulated, leading to increased 25‐HC synthesis and secretion, which aggravates pulmonary inflammation and tissue damage [47]. Furthermore, macrophage‐derived 25‐HC has been shown to promote vascular inflammation, atherogenesis, and atherosclerotic lesion remodeling, highlighting its role in activating immune cells (e.g., macrophages) and amplifying inflammatory responses [48]. Recent studies also reveal that 25‐HC induces ROS generation, disrupts oxidative stress balance, and sensitizes cells to ferroptosis [49, 50]. Our investigation initially revealed upregulated CH25H expression in macrophages derived from both patients and mice with AAA. Consistently, high serum 25‐HC levels were detected in both AAA patients and experimental mice, suggesting its potential as a novel serological biomarker for AAA diagnosis. Importantly, in vivo and in vitro experiments confirmed that *Ch25h* knockdown significantly reduced Ang II ‐induced 25‐HC production, macrophage activation, and ferroptosis, ultimately mitigating AAA progression. These findings underscore CH25H/25‐HC as a critical therapeutic target for AAA.

Given the inherent risks and limited applicability of surgical interventions, recent research efforts have focused on developing effective pharmacological treatments for AAA. While certain therapeutic agents, such as antihypertensive drugs, lipid‐lowering drugs, antiplatelet agents, and anti‐inflammatory compounds, have demonstrated partial efficacy, their clinical outcomes remain suboptimal. Notably, emerging macrophage‐targeted therapies have shown promising advancements in AAA treatment, exhibiting superior therapeutic potential. Building upon these developments, our study has specifically focused on the CH25H/25‐HC pathway, establishing a novel macrophage CH25H‐targeted therapeutic strategy that offers a novel approach for AAA management. Our siRNA delivery system is based on the FDA‐approved ultrasound contrast agent SonoVue. Both LNPs and SonoVue MBs are widely used in clinical situations [51], conferring favorable biocompatibility and prospects of clinical translation to this system. SI‐*Ch25h*‐LNP/MB generate microstreaming and microjets in response to appropriate acoustic irradiation, which can facilitate penetration across the abdominal aortic wall and enable efficient siRNA delivery into macrophages residing within the abdominal aortic tissues. Furthermore, owing to the deep tissue penetration and focal tunability of ultrasound, gene delivery can be confined to the intended site, minimizing systemic exposure. Our strategy paves the way for highly efficient and targeted therapy for AAA. This approach is readily applicable in a clinical setting, as it simply combines intravenous injection with ultrasound detection, enabling simultaneous diagnosis and treatment.

We acknowledge several limitations in the present study. First, regarding cellular specificity, while bone marrow transplantation and AAV‐CD68‐sh*Ncoa4* approaches strongly support a macrophage‐dependent mechanism, they lack absolute cell specificity. Future studies using lineage‐specific conditional knockout mice (e.g., *Lyz2*‐Cre; *Ncoa4* ^fl/fl^ or *Cx3cr1*‐Cre; *Ncoa4* ^fl/fl^) are warranted to definitively establish the cell‐autonomous role of NCOA4 in macrophage‐driven AAA progression. Second, although 25‐HC exhibits strong translational potential, large‐scale, prospective clinical cohorts are essential to validate its diagnostic efficacy as an AAA biomarker. Third, the multiple murine AAA models employed necessitate varying diets, potentially introducing metabolic variability. However, since no single model perfectly recapitulates human AAA, the consistent phenotypes observed across these distinct models reinforce the robustness and clinical translatability of our findings. Fourth, we utilized exclusively male mice due to the significantly higher incidence of AAA in males. This single‐sex approach remains a limitation of our study. Therefore, validating our findings in female murine models is essential to elucidate potential sex‐specific mechanisms prior to future clinical translation. Finally, the exact intermediate mediating NCOA4‐dependent STAT1 Ser727 phosphorylation remains undetermined. Given that STAT1 lacks intrinsic kinase activity, NCOA4 may function as a scaffold to recruit kinases, sterically hinder phosphatases, or indirectly modulate upstream inflammatory/ROS homeostasis. Identifying these specific kinases or phosphatases represents a crucial direction for future research.

In conclusion, the identification of NCOA4 functioning as a transcriptional co‐regulator in macrophage activation and ferroptosis might broaden our understanding of the multiple biological functions of NCOA4. Our study unveils a previously undescribed NCOA4/STAT1/CH25H axis that contributes to AAA, providing new insights into the transcriptional networks of AAA pathogenesis and, most importantly, offering novel biomarkers and therapeutic strategies for AAA management.

## 4 Experimental Section

### 4.1 Human Study

All human studies were approved by the Human Research Ethics Committee of Renji Hospital, School of Medicine, Shanghai Jiao Tong University (KY2025‐0006‐C). The study was performed according to the criteria set by the Declaration of Helsinki (2013). Written informed consent was obtained from all individual participants included in the study. All study participants were recruited after providing informed consent and with approval by the Ethics Committee of Renji Hospital, School of Medicine, Shanghai Jiao Tong University.

Human AAA tissue samples were obtained from patients who underwent open surgical repair. Healthy abdominal aorta samples from brain‐dead organ donor patients were used as controls. The management of AAA patients was performed according to the 2018 Society of Vascular Surgery Practice Guidelines on the care of patients with an AAA [52]. In another series of studies, venous blood samples were collected from 10 AAA patients and 10 healthy donors. There were non‐significant differences in age, sex, general diseases, and medication between groups. Blood samples were collected in Blood Collection Tubes and Plasma Preparation Tubes (Becton Dickinson, Plymouth, UK) upon initial diagnosis, preceding any pharmacological treatment. Samples were centrifuged (10 min, 3000 × *g*, 4°C) immediately after collection and aliquots of plasma and serum were stored at −80°C. Peripheral blood mononuclear cells were isolated as previously described [53]. Briefly, peripheral blood mononuclear cells were isolated by centrifugation on a Ficoll gradient (Ficoll‐Paque PLUS, GE Healthcare Life Sciences). Monocytes were selected using antibody‐coated magnetic beads and magnetic columns according to the manufacturer's instructions (CD14 MicroBeads, Miltenyi Biotec).

### 4.2 Animal Experiment Procedures

The protocols of animal experiments in this study were approved by the Animal Ethics Committee of Renji Hospital, School of Medicine, Shanghai Jiao Tong University (RJ2024‐176B), and all the procedures were conducted following the National Institutes of Health (NIH) Guidelines for the Care and Use of Laboratory Animals. All mice used in this study were housed under specific pathogen‐free conditions at a temperature of 23°C with 60% relative humidity and a 12 h dark/light cycle. *Ncoa4* ^−/−^ mice on a C57BL/6J background were produced by Shanghai Biomodel Organism Co., Ltd. Briefly, heterozygous *Ncoa4* ^+/−^ mice were hybridized to produce homozygous *Ncoa4* ^−/−^ mice and littermate *Ncoa4* ^+/+^ mice as wild‐type controls. Male mice aged 8 weeks were used in this study. To determine the effect of *Ch25h* knockdown on AAA formation, male *ApoE* ^−/−^ mice (GemPharmatech Co., Ltd.) aged 8 weeks were used. For the induction of AAA modeling, anesthesia was initially induced in mice with 2.5% isoflurane. Throughout the mini‐pump implantation procedure, anesthesia was maintained using 1.5% isoflurane. At the end of the experiments, euthanasia was performed via intravenous injection of a lethal dose of pentobarbital sodium (150 mg/kg). All animal experiments were carried out independently by two investigators who remained blinded to the group assignments. Randomization was conducted using the RAND function in Excel to assign groups based on body weight, and assignment information was concealed from the experimenters for the duration of the study. The sample size for the mouse aortic aneurysm experiments was determined a priori with PASS software, based on preliminary data from similar AAA models previously generated in our laboratory. A minimum of 8 animals per group was deemed necessary to achieve 90% power (β = 0.1) for detecting significant differences in maximal aortic diameter at a significance level of α = 0.05. Each mouse was considered an independent experimental unit.

The Angiotensin II (Ang II)‐induced AAA models were established as reported [54, 55]. A single injection of recombinant type 8 adeno‐associated virus of murine proprotein convertase subtilisin/kexin type 9 mutants (rAAV8/D377Y‐mPCSK9, 2 × 10^11^ vg) was performed in each mouse 2 weeks prior to osmotic pump implantation, combined with a Paigen diet (TP28640, Trophic Animal Feed High‐Tech Co., Ltd, China) to induce hypercholesterolemia. The ALZET osmotic pumps (model 2004, Durect, Cupertino, CA, USA) were infused with Ang II (MCE, HY‐13948, China) or saline. For AAA modeling, mice were first subjected to 2.5% isoflurane to induce anesthesia. During the process of mini pump implantation, mice were continuously anesthetized with 1.5% isoflurane. The delivery of Ang II (1000 ng/kg/min) or saline lasted 28 days. Aneurysm progression was evaluated by ultrasound imaging, and the severity of AAA was determined by measuring the maximal aortic diameters using a caliper at sacrifice. Macroscopic photos of aortas were taken with a Nikon camera, and periadventitial tissues were removed before taking pictures. We used the well‐accepted criterion to define AAA: the maximal aortic diameter of an aneurysm is at least 1.5 times the aortic diameter of mice infused with normal saline [56]. Mice that died prior to the experiment termination were excluded from further analysis except for calculating mortality and rupture rates. To determine the causes of death, necropsies were performed. The fatal events included isolated thoracic aortic dissection/rupture, combined thoracic and abdominal aortic rupture, and isolated abdominal aortic rupture. Mice with isolated thoracic aortic dissection/rupture were excluded from the survival analysis.

Bone marrow transplantation experiments were performed as previously reported [21]. Briefly, mice aged 6 weeks were maintained on antibiotic water containing neomycin (100 mg/L) and polymyxin B sulphate (10 mg/L) for 7 days before and 14 days after transplantation. Recipient mice were subjected to total body irradiation (9 Gy) and injected intravenously with 5 × 10^6^ bone marrow cells from pools of bone marrow from *Ncoa4* ^−/−^ and *Ncoa4* ^+/+^ donor mice. Tail clip samples and peritoneal macrophages were used for genotyping and confirmation of reconstitution. After 2 weeks of recovery, mice aged 8 weeks were then subjected to AAA modeling as previously described.

To study the effect of in vivo *Ch25h* knockdown in macrophages on AAA, male *ApoE* ^−/−^ mice (GemPharmatech Co., Ltd) aged 8 weeks were used. An II‐induced AAA model was performed as previously described. For histological analysis, aortas were flushed with saline and subsequently submerged in paraformaldehyde for fixation. For molecular experiments, aortas were quickly removed, flushed, and then stored in liquid nitrogen.

To determine the effect of macrophage‐specific NCOA4 deficiency on AAA formation, male *ApoE* ^−/−^ mice aged 6 weeks were randomly divided into 2 groups and injected with pAAV‐hCD68‐MCS‐EGFP‐3xFLAG‐miR30shRNA(NC)‐WPRE (AAV‐Sh*Ctrl*) or pAAV‐hCD68‐MCS‐EGFP‐3xFLAG‐miR30shRNA(Ncoa4)‐WPRE (AAV‐Sh*Ncoa4*) (5 × 10^11^ vg). Ang II‐induced AAA model was established 2 weeks after AAV injection. Mice were fed the Western diet during the modeling. In another set of experiment, male *ApoE* ^−/−^ mice aged 6 weeks were randomly divided into 4 groups and injected with pAAV‐hCD68‐MCS‐EGFP‐3xFLAG‐miR30shRNA(NC)‐WPRE (AAV‐Sh*Ctrl*) or pAAV‐hCD68‐MCS‐EGFP‐3xFLAG‐miR30shRNA(Ncoa4)‐WPRE (AAV‐Sh*Ncoa4*) and pAAV‐hCD68‐MCS‐EGFP‐3xFLAG‐miR30shRNA(NC)‐WPRE (AAV‐Sh*Nc*) or pAAV‐hCD68‐MCS‐EGFP‐3xFLAG‐miR30shRNA(Stat1)‐WPRE (AAV‐Sh *Stat1*) (5 × 10^11^ vg). Ang II‐induced AAA model was established 2 weeks after AAV injection. Mice were fed the Western diet during the modeling.

Systolic blood pressure (SBP) was evaluated using a non‐invasive tail‐cuff method (BP‐2010E; Softron) as previously reported. Briefly, mice were acclimated to the system and restraint procedure for seven consecutive days prior to the actual measurements. During the procedure, mice were placed in restraint tubes maintained at 37°C, and their tails were passed through the cuff sensor. Measurements were conducted at the same time daily for 20 min, with at least six valid readings obtained per animal.

### 4.3 Vascular Ultrasound Imaging

The abdominal hair of mice was removed using depilatory cream prior to detection. For ultrasound detection, experiments were carried out independently by two investigators who remained blinded to the group assignments. Mice were first subjected to 2.5% isoflurane to induce anesthesia. After inducing anesthesia, mice were placed on the heating panel and continuously anesthetized with 1.5% isoflurane. Images were obtained by the *Vevo* 2100 high‐resolution imaging system (Visual Sonics, Inc., Toronto, Canada). The images of abdominal aortas were captured at end‐systole and recorded on both long axis and short axis.

### 4.4 RNA Interference, Plasmid Construction, AAV Production and Adenovirus Generation

RNAi was performed using siRNAs, synthesized by GenePharma (Shanghai, China). Sequences of *Ch25h*‐siRNA are listed in Table S3. The siRNA was dissolved in RNase‐free water. PMs were seeded into 6‐well plates, and the culture medium was replaced before transfection. siRNA or SI‐Scramble was mixed with the reagent Lipofectamine 3000 (Invitrogen) for 15 min at room temperature (RT) before transfection. After 8 h of transfection, the culture medium was replaced, and cells were incubated for an additional 48 h. The efficacy of RNA interference was verified by Western Blot.

The NCOA4‐Flag plasmid, TRIM3‐HA plasmid, STAT1‐HA plasmid, and UB‐Myc plasmid were constructed by Obio Technology Corporation (Shanghai, China). The promoter regions, including Chr19: 34452549–34454548 (‐) for *Ch25h*, were amplified from mouse genomic DNA and inserted into the pGL3‐Basic vector to construct a wild‐type *Ch25h* firefly reporter. The firefly reporter containing the mutant *Ch25h* promoter was constructed using the Mut Express Universal Fast Mutagenesis Kit (Vazyme, Nanjing, China). The pRL‐TK vector (Renilla luciferase, Promega, USA) was used as an internal control for normalizing transfection efficiency.

The adeno‐associated virus (AAVs) rAAV8/D377Y‐mPCSK9 used in this study was constructed and packaged by Vigene Bioscience (Jinan, China). Adenoviruses encoding mouse Flag‐tagged NCOA4 and Flag‐tagged CH25H adenovirus were generated by Obio Technology Corporation (Shanghai, China). The AAVs including pAAV‐hCD68‐MCS‐EGFP‐3xFLAG‐miR30shRNA(NC)‐WPRE, pAAV‐hCD68‐MCS‐EGFP‐3xFLAG‐miR30shRNA(Ncoa4)‐WPRE, pAAV‐hCD68‐MCS‐EGFP‐3xFLAG‐miR30shRNA(Stat1)‐WPRE and pAAV‐hCD68‐MCS‐EGFP‐3xFLAG‐miR30shRNA(NC)‐WPRE were constructed and packaged by OBiO Technology (Shanghai, China) Corp., Ltd.

### 4.5 Histological Assay and Immunofluorescent Staining

Mice were euthanized via a single intravenous injection of a lethal dose of pentobarbital sodium (150 mg/kg), after which their aortas were harvested, fixed, and embedded in paraffin. Serial sections of 5 µm thickness were then prepared for pathological staining or immunofluorescent (IF) staining. Hematoxylin and eosin (H&E), Masson's trichrome, and elastic Van Gieson staining were performed to evaluate morphological changes, collagen deposition, and elastin degradation, respectively. The images were acquired using a NanoZoomer S360 Digital slide scanner (Hamamatsu, Japan). The extent of elastin degradation in EVG‐stained sections was assessed using a semi‐quantitative 4‐grade scale, with the following criteria: Grade 1, No degradation; Grade 2, Mild degradation; Grade 3, Severe degradation; Grade 4, Aortic rupture. Preparation of sections and image analysis were carried out independently by two investigators who remained blinded to the group assignments.

For IF staining, selected sections underwent gradient rehydration, antigen repair, and blocking, and were incubated with primary antibodies overnight at 4°C. Antibodies used in IF staining are listed in Table S4. The other day, sections were washed with PBS for 3 times and incubated with Alexa Fluor 488‐/555‐conjugated secondary antibodies (1:1000, Invitrogen, USA) at RT in a humid chamber for an hour. DAPI (P0131, Beyotime Biotechnology, Shanghai, China) was added to visualize the nuclei before sealing. Images were captured by an electron microscope (DS‐Ri2, Nikon, Japan) and analyzed by ImagePlus.

### 4.6 Biochemical Analysis

All mice were fasted overnight and weighed before collecting blood. The plasma was separated and measured for total cholesterol, total triglycerides, HDL‐C, and LDL‐C using commercial kits (Nanjing Jiancheng Technology, A110‐1‐1, A111‐1‐1, A112‐1‐1, A113‐2‐1) following the manufacturer's instructions.

### 4.7 Cell Isolation and Culture

Primary peritoneal macrophages (PMs) were obtained using *Ncoa4* ^−/−^ mice and *Ncoa4* ^+/+^ mice aged 8–10 weeks. Mice were intraperitoneally injected with 1 mL sterile 6% starch solution. 3 days later, mice were euthanized via single intravenous injection of a lethal dose of pentobarbital sodium (150 mg/kg) and sterilized by immersion in 75% ethanol for 5 min. Under aseptic conditions, the abdominal cavity was opened, and the peritoneum was gently lavaged three times with 10 mL of ice‐cold sterile PBS (supplemented with 1% penicillin‐streptomycin) per wash. The lavage fluid was collected and centrifuged at 1000 rpm for 5 min. The supernatant was discarded, and the cell pellet was treated with red blood cell lysis buffer for 1 min, followed by two washes with PBS. Finally, the cells were resuspended in DMEM complete medium (supplemented with 10% FBS) and seeded in culture dishes. After 2 h of incubation at 37°C with 5% CO_2_, non‐adherent cells were removed. After 24 h, the PM was subjected to transfection or Ang II administration.

Primary bone marrow‐derived macrophages (BMDMs) were obtained using *Ncoa4* ^+/+^ mice and *Ncoa4* ^−/−^ mice aged 8–10 weeks. Mice were euthanized via a single intravenous injection of a lethal dose of pentobarbital sodium (150 mg/kg) and sterilized by immersion in 75% ethanol for 5 min. The femurs and tibias were excised, and the surrounding muscular tissues were carefully removed. The ends of the bones were cut, and the bone marrow cavity was gently flushed three times with ice‐cold sterile PBS (supplemented with 1% penicillin‐streptomycin) using a 1 mL syringe fitted with a 25‐gauge needle. The bone marrow suspension was collected and centrifuged at 1000 rpm for 5 min. The supernatant was discarded, and the cell pellet was treated with red blood cell lysis buffer for 1 min, followed by two washes with PBS. Finally, the cells were resuspended in DMEM complete medium (supplemented with 10% FBS and 20 ng/mL recombinant murine M‐CSF) and seeded in culture dishes. The cells were incubated at 37°C with 5% CO_2_, and the culture medium was refreshed every 3 days. After 7 days of incubation, non‐adherent cells were removed. The mature BMDMs were then subjected to transfection or Ang II administration.

Human embryonic kidney 293T (HEK293T) cells were obtained from the American Type Culture Collection (ATCC; USA). HEK293T cells were cultured in DMEM supplemented with 10% FBS in a humidified culture incubator at 37°C with 5% CO_2_.

### 4.8 Co‐Immunoprecipitation Assay

Co‐Immunoprecipitation Assay was performed to verify the interaction between NCOA4 and TRIM3 or STAT1. To verify the interaction, PMs or HEK‐293T cells were seeded into 6 cm dishes. After corresponding administration, the proteins were extracted using NP‐40 lysis buffer with an added protease inhibitor cocktail (AR0107, Boster Biological Technology, Wuhan, China). Protein concentration was determined by the Pierce BCA Protein Assay Kit (23225; Thermo Fisher Scientific, USA). For immunoprecipitation experiments, lysates were incubated with target antibodies overnight at 4°C on a rotary shaker. Species‐ and isotype‐matched IgG was used as the negative control. The lysates were then incubated with 10–20 µL of Pierce Protein G Magnetic Beads (88847; Thermo Fisher Scientific, USA) on the rotary shaker at 4°C for 2 h. Following incubation, the beads were washed three times with lysis buffer. The protein complexes were denatured and separated by SDS‐PAGE electrophoresis. The procedure for Western blot has been described previously. VeriBlot for IP Detection Reagents (Abcam, ab131366) were used to detect bound primary antibodies to avoid interference from the IgG heavy chain during chemiluminescence. The antibodies used for Western blot are listed in Table S4.

### 4.9 Western Blotting Analysis

Total protein from cells and aortic tissues was extracted using RIPA buffer with an added protease inhibitor cocktail (AR0102, Boster Biological Technology, Wuhan, China). Denatured proteins were separated by electrophoresis using SDS‐polyacrylamide gels and transferred to PVDF membranes (0.45 µm, Millipore). Pre‐stained protein ladders (SB‐WB009; Share‐Bio, China or 26616; Thermo Fisher Scientific, USA) were used for molecular weight reference. Membranes were blocked with 5% skimmed milk at RT for 1 h and incubated with primary antibodies at 4°C overnight. HRP‐conjugated secondary antibodies (Jackson ImmunoResearch, USA) and ECL detection reagent (SB‐WB012; Share‐Bio, China) were used for detecting bound antibodies. The Tanon Chemi 5200 Multi automatic luminescence imaging system (Tanon, Shanghai, China) was used for chemiluminescence. Antibodies used in the experiments are listed in Table S4.

### 4.10 Reverse Transcription‐Quantitative Polymerase Chain Reaction (RT‐qPCR)

Total RNA of cells and aortic tissues was extracted using RNAiso Plus (9109, Takara) following the manufacturer's instructions. 1 µg RNA from each sample was reversely transcribed into cDNA using HiScript IV RT SuperMix for qPCR (R423‐01, Vazyme, Nanjing, China). RT‐qPCR was performed using Taq Pro Universal SYBR qPCR Master Mix (Q712‐03, Vazyme, Nanjing, China) following the manufacturer's instructions. Primers used in the experiments are listed in Table S3. LightCycler 480 Instrument II (Roche LifeScience) was used for RT‐qPCR experiments and data analysis.

### 4.11 Immunoprecipitation‐Mass Spectrometry (IP/MS)

As shown in Figure S1A, the PMs were transfected with *Noca4*‐overexpressing adenovirus for 48 h. IP was performed as described above. Electrophoresis was performed first, then the gel was stained with Coomassie brilliant blue staining (Beyotime), and the bands of interest were cut for liquid chromatography‐mass spectrometry analysis, which was performed and analyzed by OE Biotech Co. Ltd. (Shanghai, China).

### 4.12 RNA Sequencing

RNA‐sequencing was conducted by OE Biotech Co. Ltd. (Shanghai, China). Briefly, total RNA from PMs from *Ncoa4* ^−/−^ mice and *Ncoa4* ^+/+^ mice (n = 4 per group) was isolated using RNAiso Plus. The data of RNA‐sequencing was analyzed in R (V3.2.0). DESeq2 package was used to identify genes differentially expressed between the two groups. The *p*‐values were adjusted using the Benjamini–Hochberg approach. The thresholds were set to a false discovery rate (FDR) < 0.05 and a fold change > 2. Volcano plots were generated using the ggplot2 package. The Kyoto Encyclopedia of Genes and Genomes (KEGG) enrichment analysis was conducted in R utilizing the hypergeometric distribution for statistical assessment. The RNA‐seq data have now been deposited in the NCBI database under the accession number PRJNA1493069.

### 4.13 Cleavage Under Targets and Tagmentation (CUT & Tag) Sequencing and Analysis

PMs from *Ncoa4* ^−/−^ mice and *Ncoa4* ^+/+^ mice were used for CUT & Tag assay using the Hyperactive Universal CUT & Tag Assay Kit for Illumina (TD904; Vazyme Biotech Co., Ltd, China) following the manufacturer's instructions. PMs were digested using Trypsin‐EDTA (25200072; Thermo Fisher Scientific, USA), resuspended in phosphate‐buffered saline (PBS, Thermo Fisher Scientific, USA), and counted. Next, 1 × 10^6^ cells per sample were combined with 10 µL of Concanavalin A‐coated magnetic beads at RT for 10 min. Bead‐bound nuclei or cells were then incubated with the indicated primary antibody or normal IgG (2729, Cell Signaling, USA) overnight at 4°C. Subsequently, cells were incubated with the goat anti‐rabbit IgG antibody (Ab207; Vazyme Biotech Co., Ltd, China) for 1 h at RT. DNA tagmentation was performed by incubating the samples with Hyperactive pA/G‐Transposase in Tagmentation buffer. Libraries were amplified using the TruePrep Index Kit V2 for Illumina (TD202; Vazyme Biotech Co., Ltd, China) and purified using VAHTS DNA Clean Beads (N411; Vazyme Biotech Co., Ltd, China). Library size analysis was performed using the Agilent 4200 TapeStation system (Agilent Technologies, USA). Finally, sequencing was carried out on the Illumina NovaSeq 6000 platform, generating 150 bp paired‐end reads for subsequent analysis.

Adapter and low‐quality reads were removed using FASTP software (Version 0.20.1). The clean reads were then aligned to reference genome sequences using the BWA program (Version 0.7.12). Peak calling was performed using MACS2 software (Version 2.2.7.1) with a *q*‐value cutoff of < 0.05, utilizing the BAM file generated by uniquely mapped reads. Deeptools (Version 3.5.3) was used to generate aggregation and heatmap plots centered at the transcriptional start sites of mm10 gene annotations. Differential peaks were assessed using the R package DESeq2 (Version 1.40.2) with a *p*‐value cutoff of < 0.05 and log2 fold change > 1. Peak annotation analysis was carried out using BEDTools software (Version 2.25.0) to determine the relationship between peak regions and various functional genomic regions. The CUT & Tag sequencing data have now been deposited in the NCBI database under the accession number PRJNA1493325.

### 4.14 Integrated Bioinformatics Analysis

Human AAA dataset (GSE183464) and mouse AAA dataset (GSE17901) were downloaded from the Gene Expression Omnibus database (https://ncbi.nlm.nih.gov/geo/). These were used to identify DEGs in AAA. Data analysis was performed using R project (R version 4.0.3).

### 4.15 Chromatin Immunoprecipitation (ChIP) Assay

ChIP assay was performed using the SimpleChIP enzymatic ChIP kit (#9005, Cell Signaling Technology) according to the manufacturer's instructions. Briefly, PMs from *Ncoa4* ^−/−^ mice and *Ncoa4* ^+/+^ mice were seeded into 15 cm dishes, then cross‐linked with formaldehyde for 10 min. Glycine solution was added to stop cross‐linking. Cells were lysed and sonicated to produce DNA fragments. A total of 2% of the supernatant was used as the chromatin input before adding antibodies. Specific antibodies were added to the solution and incubated at 4°C overnight. 20 µL Pierce Protein G Magnetic Beads (88848, Thermo Fisher Scientific, Waltham, MA, USA) were added to each sample for IP. After washing, de‐crosslinking, eluting, and purifying, the purified DNA was used for qPCR. The primers used in ChIP‐qPCR were listed in Table S3.

### 4.16 Luciferase Reporter Assays

HEK‐293T cells were co‐transfected with plasmids of firefly luciferase reporters of WT or MUT *Ch25h* promoters, Renilla luciferase combined with *Stat1 or Ncoa4* expression, or empty vector using Lipo8000 transfection reagent (C0533, Beyotime Biotechnology, Shanghai, China). The activity of both firefly and Renilla luciferases was measured using the Dual‐Luciferase Reporter Assay system (E1910, Promega) following the manufacturer's instructions, and firefly luciferase activity was normalized to Renilla activity and expressed as relative luciferase activity.

### 4.17 MitoSOX Red Staining

The production of superoxide by mitochondria was measured by MitoSOX Red Mitochondrial Superoxide Indicator (M36007, Thermo Scientific, USA) according to the manufacturer's instructions. The PMs were seeded into 12‐well plates and subjected to transfection and Ang II administration. After the administration, the cells were washed gently 3 times with PBS. Then the probe (5 µM) was added to the culture medium. After incubating at 37°C for 20 min in the dark, the cells were washed 3 times with PBS to remove the free probe, and images were captured by confocal microscopy (Nikon, Japan) and measured by Image J.

### 4.18 Enzyme‐Linked Immunosorbent Assay (ELISA)

The levels of 25‐Hydroxycholesterol (25‐HC) in serum from AAA patients or healthy donors and the levels of 25‐HC in serum from mice were determined by commercial ELISA kits (Shanghai Enzyme‐linked Biotechnology Co., Ltd., China), according to the manufacturer's instructions. First, 50 µL of samples or standards were dispensed into each well, followed by the addition of 100 µL of horseradish peroxidase (HRP)‐conjugated detection antibody. The plate was sealed with adhesive film and incubated at 37°C for 60 min. After incubation, the liquid was discarded, and the plate was blotted dry on absorbent paper. The wells were then washed five times with 350 µL of washing buffer (1 min incubation per wash, followed by removal and drying). Subsequently, 50 µL each of substrate A and B were added to each well, and the plate was incubated at 37°C in the dark for 15 min. The reaction was terminated by adding 50 µL of stop solution, and the optical density (OD) was measured at 450 nm.

To determine the 25‐HC produced by PMs, PMs were seeded into a 6‐well plate and transfected with si‐Scramble or si‐*Ch25h*. After treatment with Ang II for 48 h, 1 mL of culture medium from each well was collected and centrifuged at 1000 g, 4°C for 20 min. The supernatants were collected and subjected to detection as previously described using the corresponding ELISA kit. The cells in 6‐well plates were collected and lysed in RIPA buffer and then centrifuged at 12 000 g, 4°C for 15 min. The supernatants were collected, and the protein concentration was determined using the Pierce BCA Protein Assay Kit.

### 4.19 Measurement of GSH/GSSG

PMs were seeded into a 6‐well plate and transfected with si‐Scramble or si‐*Ch25h*. After treatment with Ang II for 48 h, cells were washed with PBS for 3 times and collected. The levels of glutathione (GSH) and glutathione disulfide (oxidized glutathione, GSSG) were evaluated by a GSH and GSSG Assay Kit (S0053, Beyotime Biotechnology, Shanghai, China), according to the manufacturer's instructions. The ratio of GSH to GSSG (GSH/GSSG) was subsequently calculated.

### 4.20 Measurement of Malondialdehyde

PMs were administered as previously described; malondialdehyde (MDA) was assessed with the commercial Kit (S0131S, Beyotime Biotechnology, Shanghai, China), according to the manufacturer's instructions. Briefly, the samples were lysed in RIPA buffer and then centrifuged at 12 000 g for 15 min. 100 µL aliquot of each cell supernatant was then reacted with the working solution, which comprised thiobarbituric acid (TBA) diluent, TBA storage solution, and antioxidants. Following a 15 min incubation at 100°C, the reaction mixtures were cooled to RT in a water bath and centrifuged at 1000 g for 10 min. The absorbance of the resulting supernatant (100 µL) was measured at 532 nm.

### 4.21 Measurement of Ferrous Iron (Fe^2+^)

PMs were administered as previously described; the relative iron concentration in cell lysates was assessed with an Iron Assay Kit (ab83366; Abcam, USA) according to the manufacturer's instructions. Cells were collected and homogenized on ice with 100 µL of iron assay buffer. The samples were then centrifuged at 16 000 g for 10 min at 4°C to separate the supernatant, which was collected for analysis. To this supernatant, 5 µL of an iron reducer was added, mixed, and incubated at 37°C for 30 min. 100 µL of iron probes were then added and thoroughly mixed. The reaction mixture was incubated for 60 min at 37°C before its absorbance was measured at 593 nm.

### 4.22 Measurement of FerroOrange

A FerroOrange probe (F374; Dojindo, Shanghai, China) was used to detect intracellular Fe^2+^ according to the manufacturer's instructions. After the indicated treatments, cells were washed with PBS for 3 times and incubated with FerroOrange working solution (1 µmol/L) for 30 min. After incubation, the cells were washed with PBS for 3 times and observed using a fluorescence microscope at an excitation of 505 nm and emission of 535 nm. The images were captured by confocal microscopy (Nikon, Japan).

### 4.23 Measurement of MitoPeDPP

A MitoPeDPP probe (M466; Dojindo, Shanghai, China) was used to detect intracellular lipid peroxide generation according to the manufacturer's instructions. After the indicated treatments, cells were washed with PBS for 3 times and incubated with MitoPeDPP working solution (1 µM) for 30 min. After incubation, the cells were washed with PBS for 3 times and observed using a fluorescence microscope at an excitation of 452 nm and emission of 470 nm. The images were captured by confocal microscopy (Nikon, Japan).

### 4.24 SI‐Ch25h‐LNP Preparation

LNPs were prepared using a previously reported microfluidic mixing method. The lipid composition consisted of 1,2‐dilinoleyloxy‐3‐dimethylaminopropane (Dlin‐MC3‐DMA), 1,2‐distearoyl‐sn‐glycero‐3‐phosphocholine (DSPC), 1,2‐dimyristoyl‐rac‐glycero‐3‐methoxypolyethylene glycol‐2000 (DMG‐PEG2000), cholesterol and DMG‐PEG2000‐MAL at a molar ratio of 50:38:1.5:10:0.5. Encapsulation of siRNA was achieved by controlled mixing in a microfluidic device, wherein an aqueous solution of SI‐*Ch25h* in 50 mM sodium citrate buffer at pH 4.0 was introduced at three folds of the flow rate and volume of the ethanolic lipid mixture. The resulting formulations were collected in dextrose 5% in water (D5W). The materials used for SI‐*Ch25h*‐LNP preparation are listed in Table S5.

### 4.25 Microbubble Preparation

SonoVue MBs, a widely used ultrasound contrast agent, which is composed of a phospholipid shell and a sulfur hexafluoride (SF6) gas core, were purchased from Bracco SpA, Milan, Italy. To induce thiol groups onto the MBs’ shell, 1% 1,2‐distearoyl‐sn‐glycero‐3‐phosphoethanolamine‐N‐(polyethylene glycol‐2000)‐thiol (DSPE‐PEG2000‐SH) was added. The materials used for MBs preparation are listed in Table S5.

### 4.26 SI‐Ch25h‐LNP/MB Preparation and Characterization

SI‐*Ch25h*‐LNP was conjugated to MB via a Michael addition reaction through the maleimide on the SI‐*Ch25h*‐LNP and the thiol on the MB in the dark at RT. The zeta potential of SI‐*Ch25h*‐LNP, MB, and SI‐*Ch25h*‐LNP/MB was analyzed using nanoparticle tracking analysis (Zetasizer Lab, Malvern). The particle size of MB and SI‐*Ch25h*‐LNP/MB was measured by the single Particle Sizing Systems (PSS) method (AccuSizer 780A Autodiluter, PSS NICOMP) and the DLS method (Zetasizer Lab, Malvern). Bright‐field images of MB and SI‐*Ch25h*‐LNP/MB were observed using an All‐in‐One Fluorescence Microscope (BZ‐X810, Keyence). The instruments used for SI‐*Ch25h*‐LNP characterization are listed in Table S6.

### 4.27 Contrast‐Enhanced Ultrasound Imaging

Ultrasound imaging (Resona R9 Pro, Mindray, Shenzhen, China) was performed with a high‐frequency linear‐array probe. The abdominal aorta was imaged with fundamental imaging at 20 MHz to optimize the imaging plane along the longitudinal axis. Contrast‐enhanced ultrasound (CEU) was performed with Contrast Pulse Sequencing, which detects the nonlinear fundamental signal component for MB. The gain was set just below visible speckle at baseline and held constant. Real‐time imaging was performed with Dual Live mode, which included both Tissue Harmonic Imaging (THI) and B image, after intravenous injection of SI‐*Ch25h*‐LNP/MB. After several seconds of Micro‐bubble Destruction mode, MB were destroyed. Subsequent post‐destruction images were acquired. The instrument used for contrast‐enhanced ultrasound imaging is listed in Table S6.

### 4.28 In Vivo Biodistribution Assessment

An in vivo fluorescence imaging system (VISQUE InVivo ART 400, Vieworks) was employed to conduct in vivo fluorescence imaging at 24 h after administration. Additionally, major organs of the mice (heart, liver, spleen, lung, and kidney) were harvested 24 h after administration for fluorescence imaging. Furthermore, aorta tissues of mice in each group were stained for fluorescence imaging and then were sealed with Antifade Mounting Medium with DAPI and scanned in full using an All‐in‐One Fluorescence Microscope (BZ‐X810, Keyence). The instruments used for in vivo biodistribution assessment are listed in Table S6.

### 4.29 Biosafety Assessment

Saline and SI‐*Ch25h*‐LNP/MB were administered intravenously to each group of C57 mice. Blood samples were collected from the mice in the control group, SI‐*Ch25h*‐LNP/MB group, and the SI‐*Ch25h*‐LNP/MB with ultrasound destruction group on day 7 following administration for complete blood count and plasma biochemical examinations, including the assessment of liver and kidney function indices. After 7 days, the major organs (heart, liver, spleen, lung, and kidney) were harvested for hematoxylin and eosin (HE) staining and subsequent analysis.

### 4.30 Statistical Analysis

All statistical analyses were performed using GraphPad Prism 10. For statistical comparisons, the Shapiro–Wilk test was performed initially to evaluate data for normality. Normally distributed data are presented as mean ± Standard Error of the mean (SEM), while non‐normally distributed data are presented as median and interquartile range. The statistical differences between groups were assessed as follows: (1) For independent samples between two groups, statistical differences were assessed using two‐tailed unpaired Student's *t*‐tests with homogeneous variances. Two‐tailed unpaired Student's *t*‐test with Welch correction was applied in the presence of heterogeneity in variances. (2) One‐way ANOVA was applied in scenarios of comparisons involving more than 2 independent groups with a single factor. For non‐normally distributed data, the Kruskal–Wallis test was used, followed by post hoc analysis via the Dunn multiple comparisons test. (3) Two‐way ANOVA followed by Tukey's multiple comparison tests was used for comparing differences among more than two groups with two factors. (4) For non‐normally distributed data, the nonparametric Mann‐Whitney U test was used for comparisons of two samples, whereas the Kruskal–Wallis test was applied for comparisons of more than two data samples. (5) Kaplan–Meier method plus a stratified log‐rank test was applied to evaluate the survival rates between groups. (6) For data with unequal variances, Brown‐Forsythe and Welch ANOVA tests were used. (7) Fisher's Least Significant Difference (LSD) post‐hoc analysis was applied following one‐way ANOVA where specified. *p*‐value < 0.05 was considered statistically significant.

## Author Contributions

**Zhinan Wu**: Conceptualization, Investigation, Writing – original draft, Writing – review & editing, Validation, Visualization, Methodology, Software, Formal analysis, Data curation. **Yuxuan Qian**: Writing – original draft, Investigation, Writing – review & editing, Visualization, Validation, Software, Formal analysis, Methodology, Data curation. **Wenyi Xu**: Validation, Formal analysis, Data curation, Visualization, Methodology. **Fei Liao**: Methodology, Validation. **Xueliang Liu**: Resources. **Xiao Kuang**: Resources. **Guqing Luo**: Data curation. **Xinjie He**: Data curation, Visualization. **Lixian Jiang**: Methodology, Supervision, Resources, Writing – review & editing, Writing – original draft, Project administration, Validation, Software. **Song Ding**: Supervision, Conceptualization, Investigation, Writing – review & editing, Funding acquisition, Resources, Project administration.

## Funding

This work was supported by the National Natural Science Foundation of China (Grant No. 82370475 and 82070477); Shanghai Eastern Talent Plan (Grant No. BJWS2024007); The Medical Discipline Construction Program of Shanghai Pudong New Area Health Commission (the Key Disciplines Program (Grant No. PWZXK‐2022‐11 and PWZXK2025‐16).

## Ethics Approvals

This study was approved by the Human Research Ethics Committee (KY2025‐0006‐C) and the Animal Ethics Committee (RJ2024‐176B) of Renji Hospital, School of Medicine, Shanghai Jiao Tong University. Human studies were performed according to the Declaration of Helsinki (2013) with written informed consent obtained from all participants. All animal procedures strictly followed the NIH Guidelines for the Care and Use of Laboratory Animals.

## Conflicts of Interest

The authors declare no conflicts of interest.

## Supporting information




**Supporting File 1**: advs78079‐sup‐0001‐SuppMat.docx.

**Supporting File 2**: advs78079‐sup‐0002‐SuppMat.pdf.

**Supporting File 3**: advs78079‐sup‐0003‐SuppMat.pdf.

## Data Availability

The data that supports the findings of this study are available from the corresponding author on reasonable request.
