# Supplementary material for: NCOA4 Accelerates Abdominal Aortic Aneurysm Formation by Regulating Macrophage Activation and Ferroptosis via STAT1–CH25H Axis
Source: Adv Sci (Weinh). 2026 Sep 29:e78079. Online ahead of print. doi: 10.1002/advs.78079 (PMC13624311; doi:10.1002/advs.78079)

Microscope images

Figure 1 E

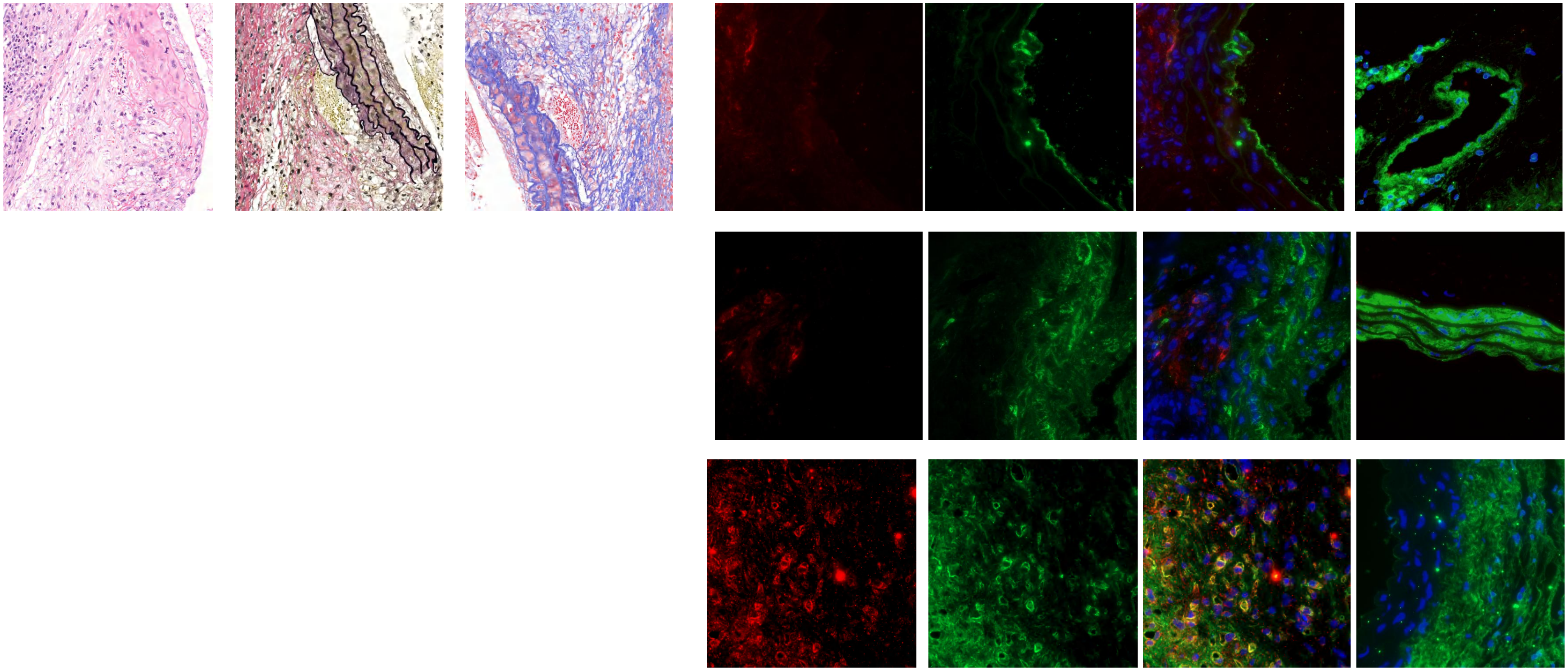

Figure 1 H

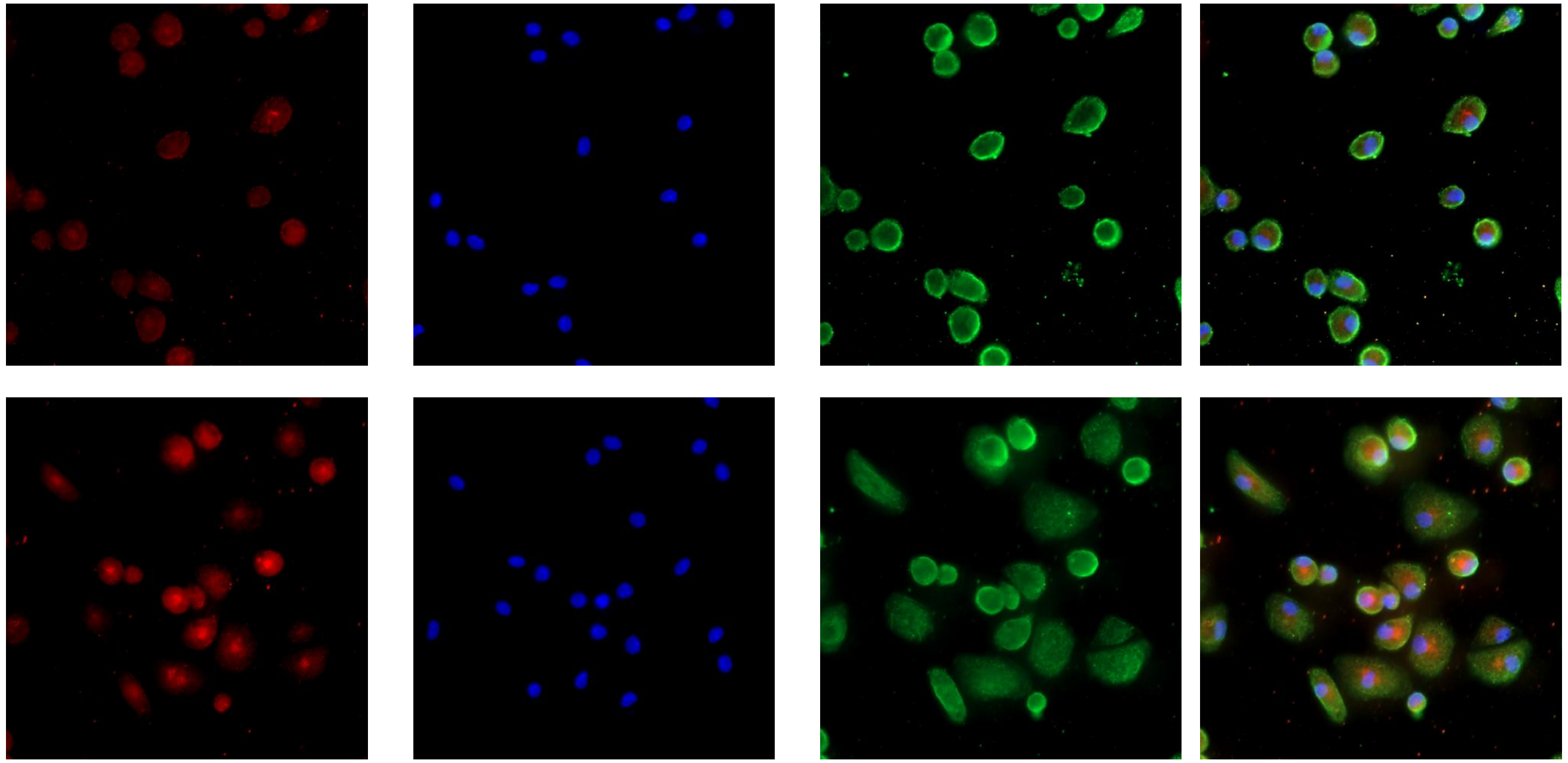

Figure 2E

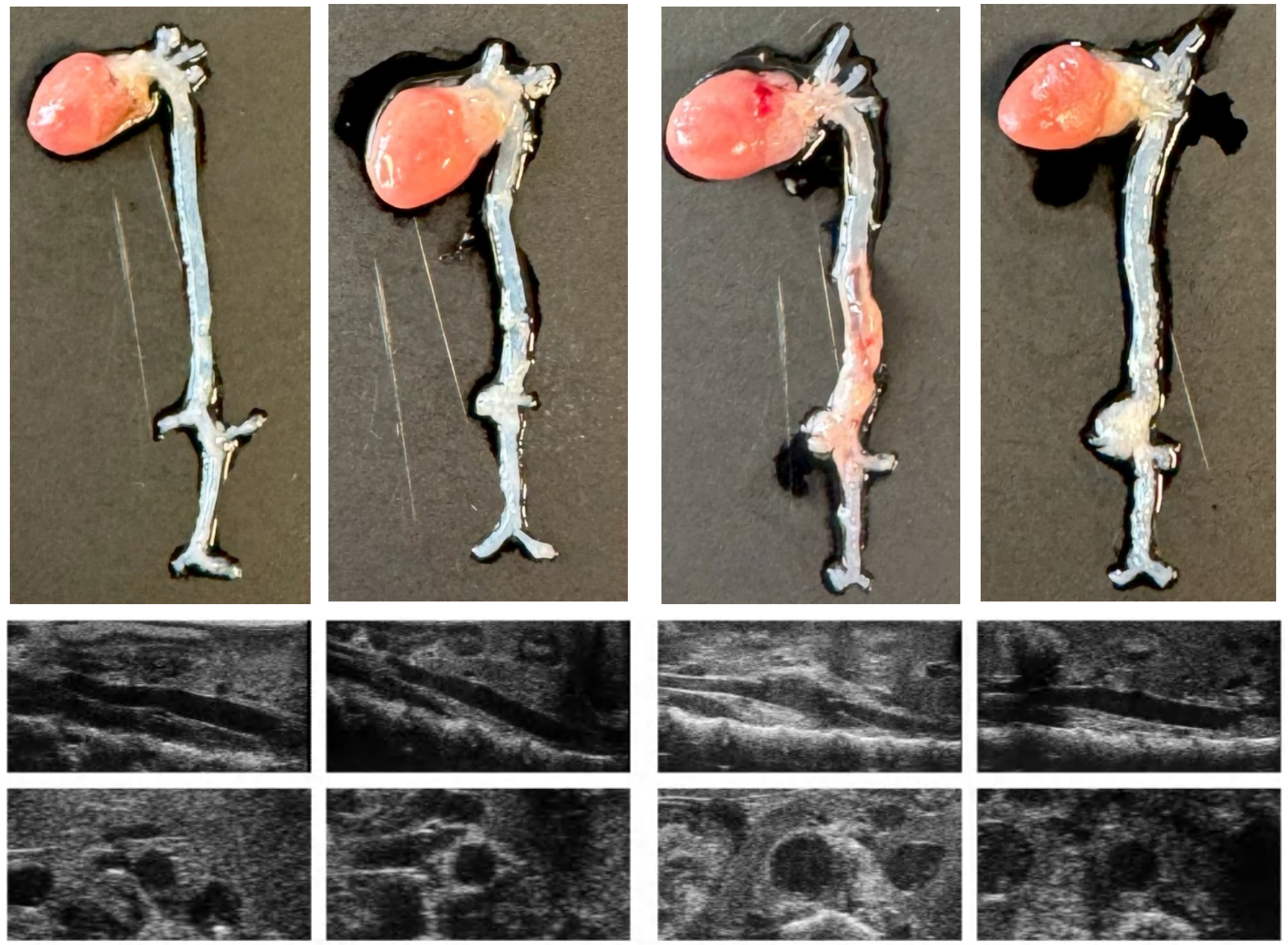

Figure 2F

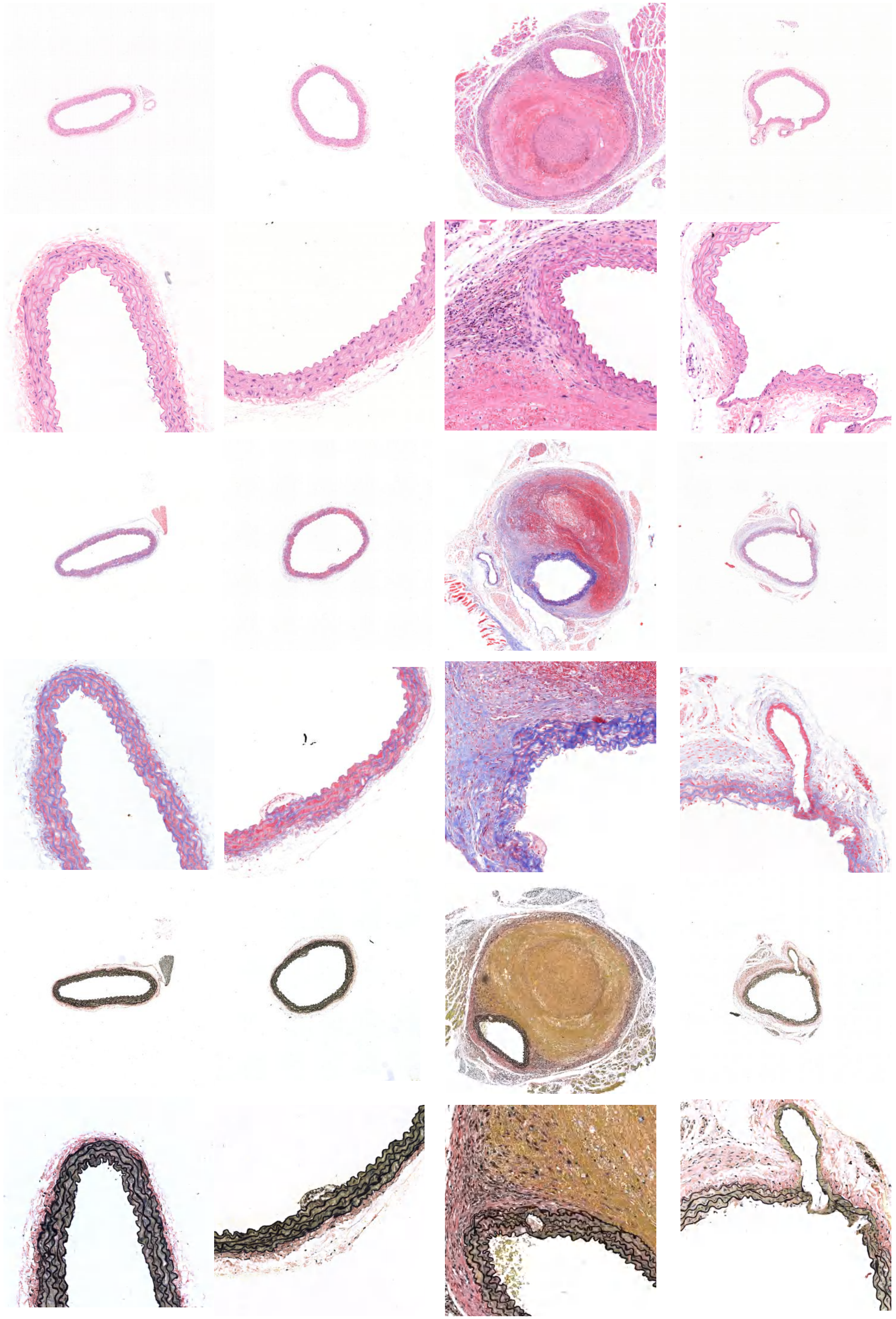

Figure 3D

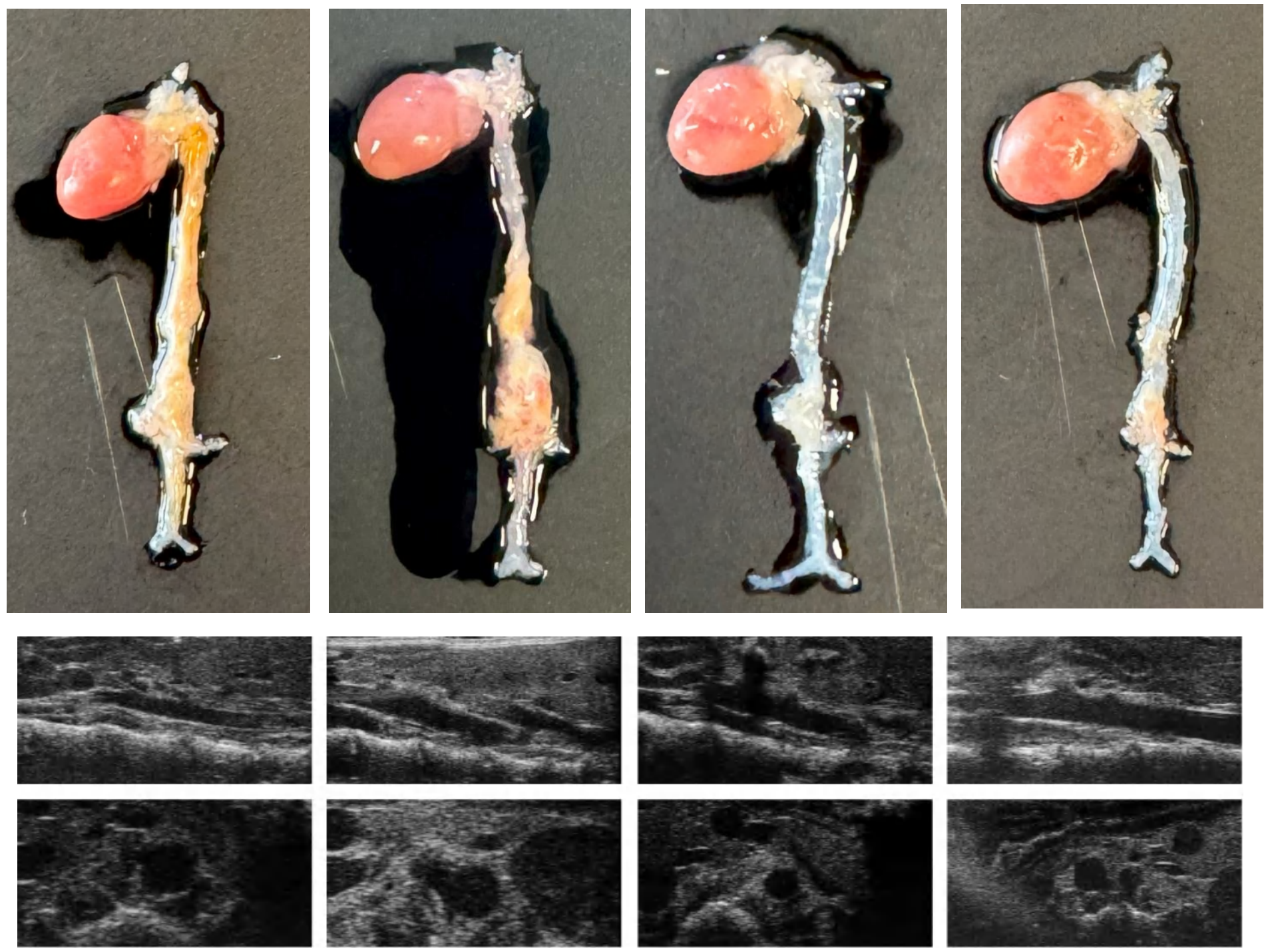

Figure 3H

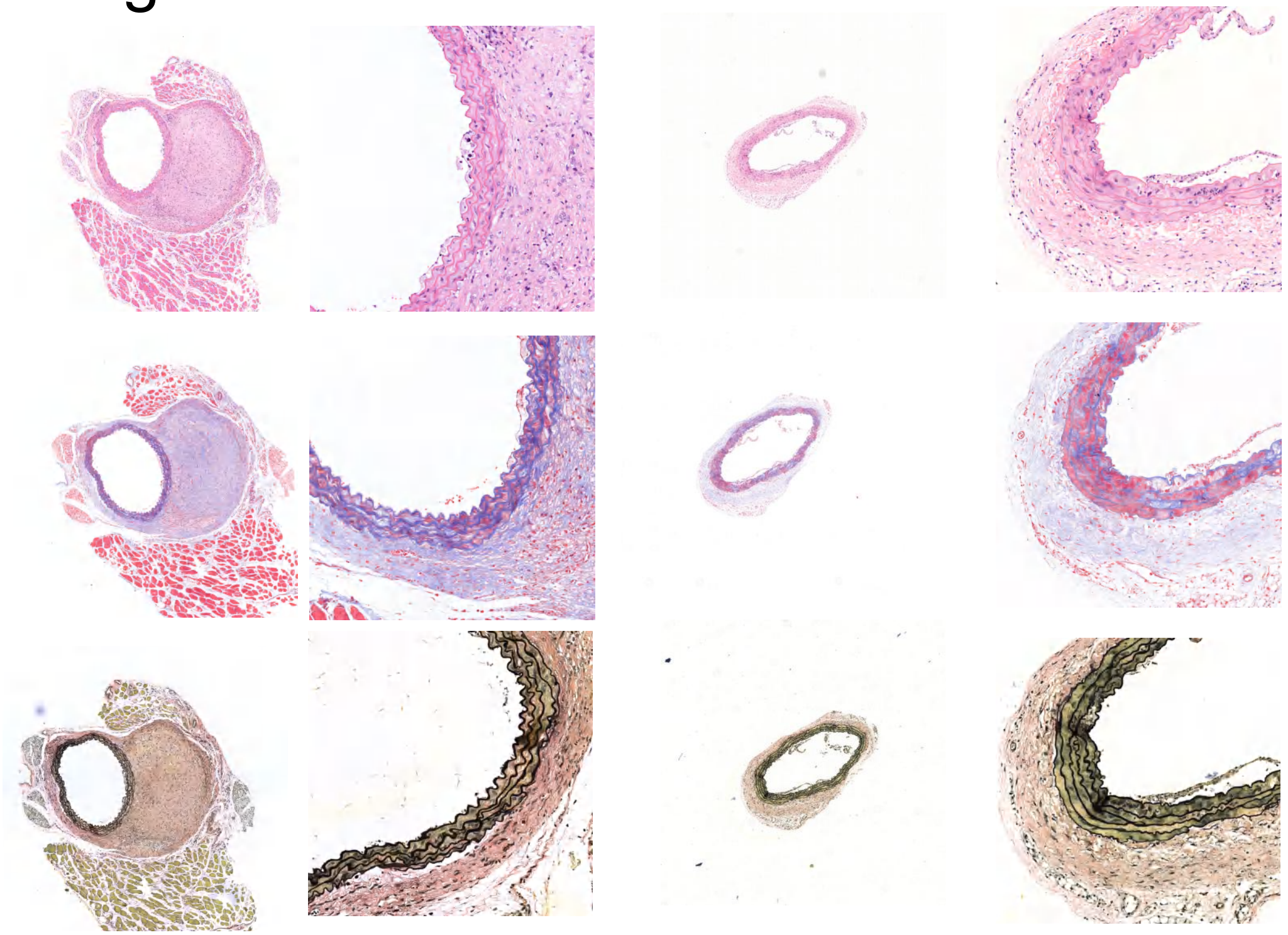

Figure 5G

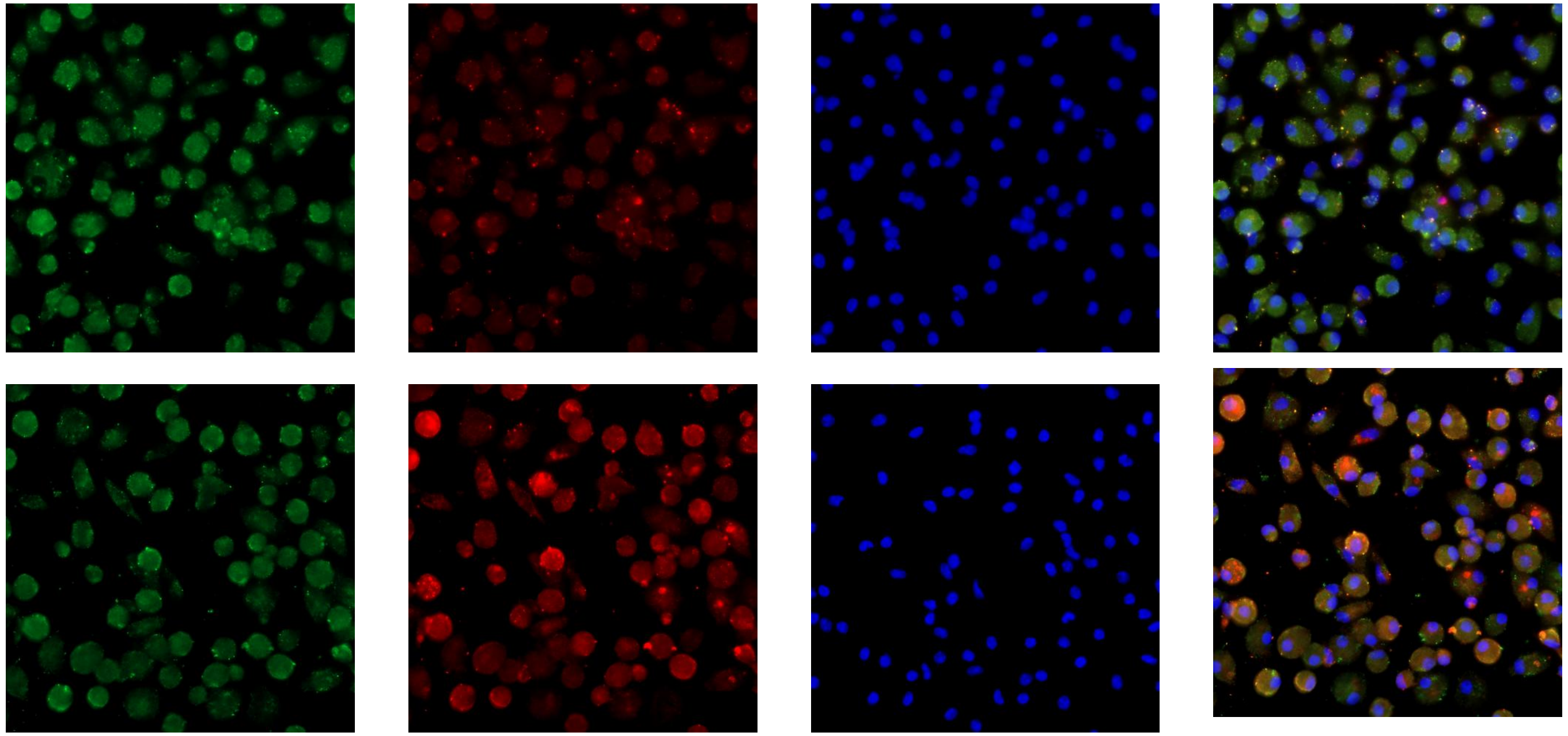

Figure 6C

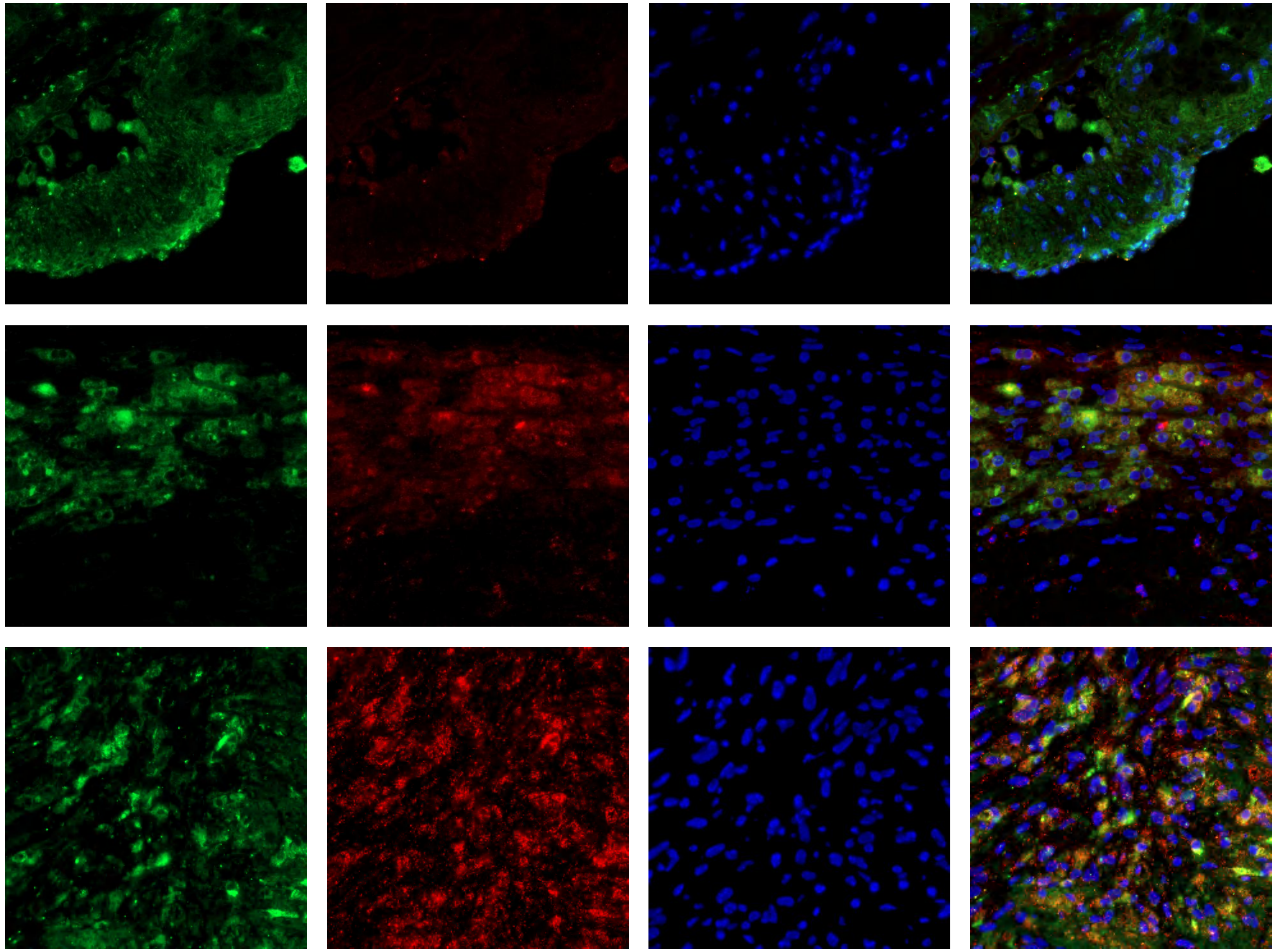

Figure 7B

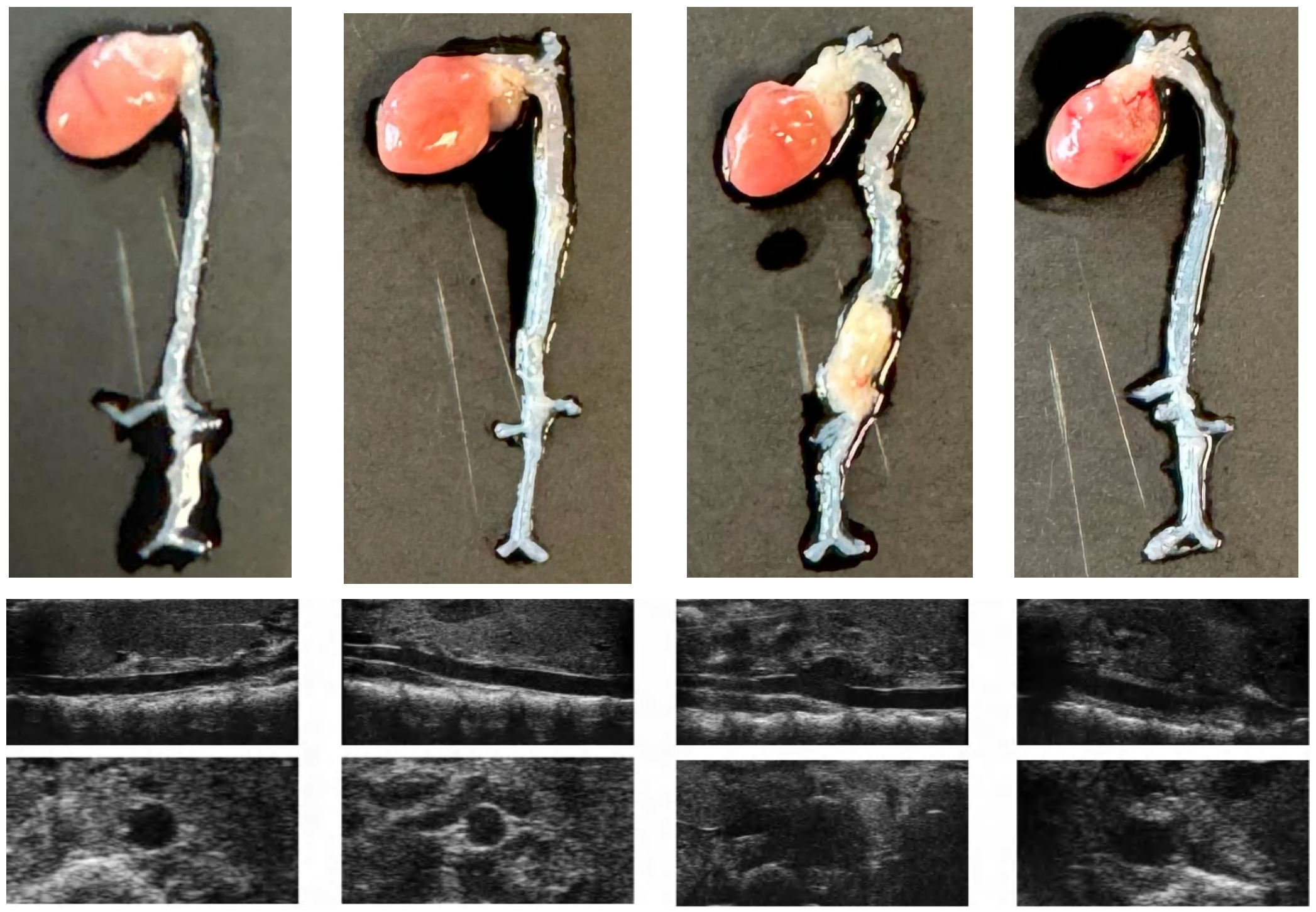

Figure 7F

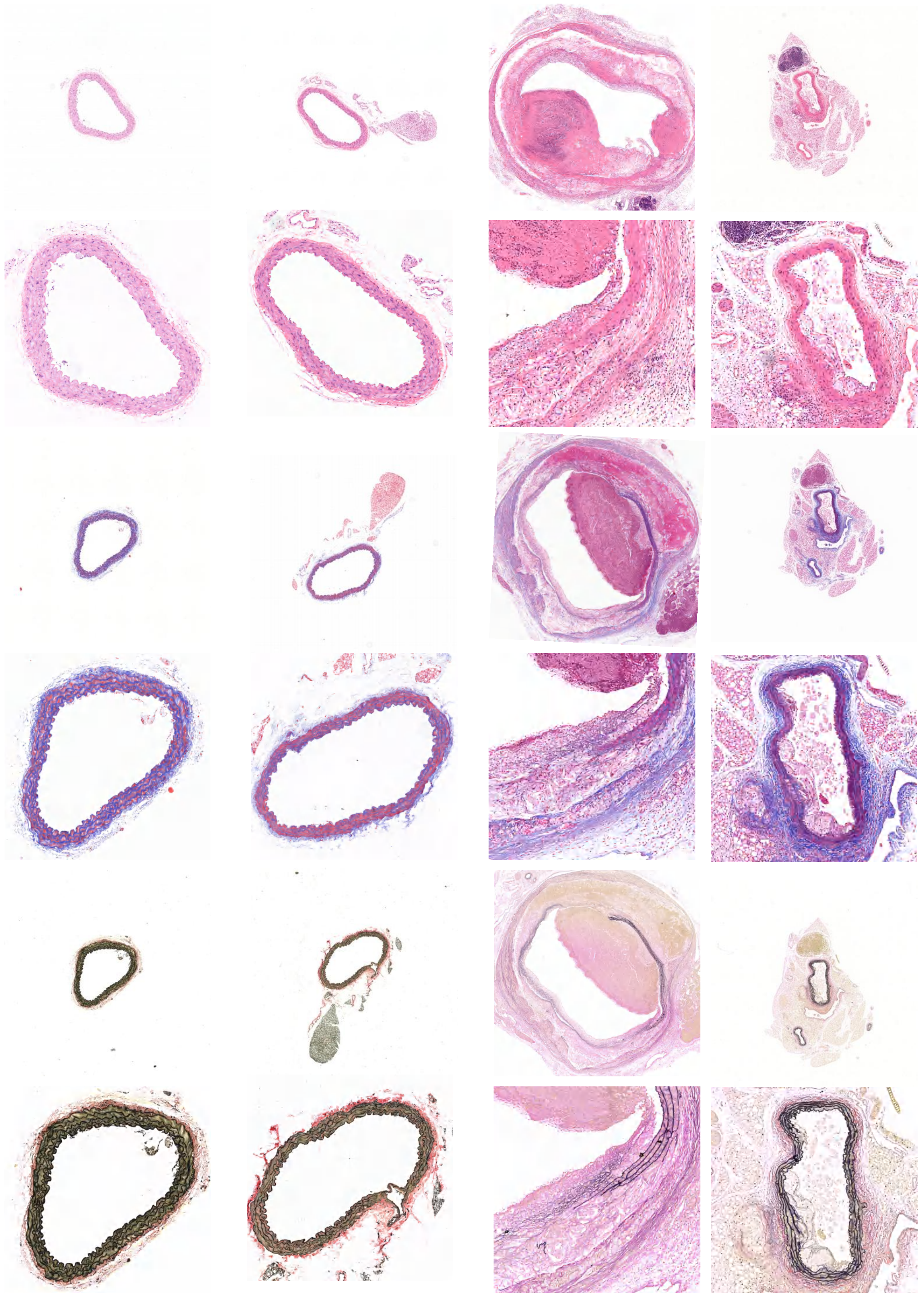

Figure S2G

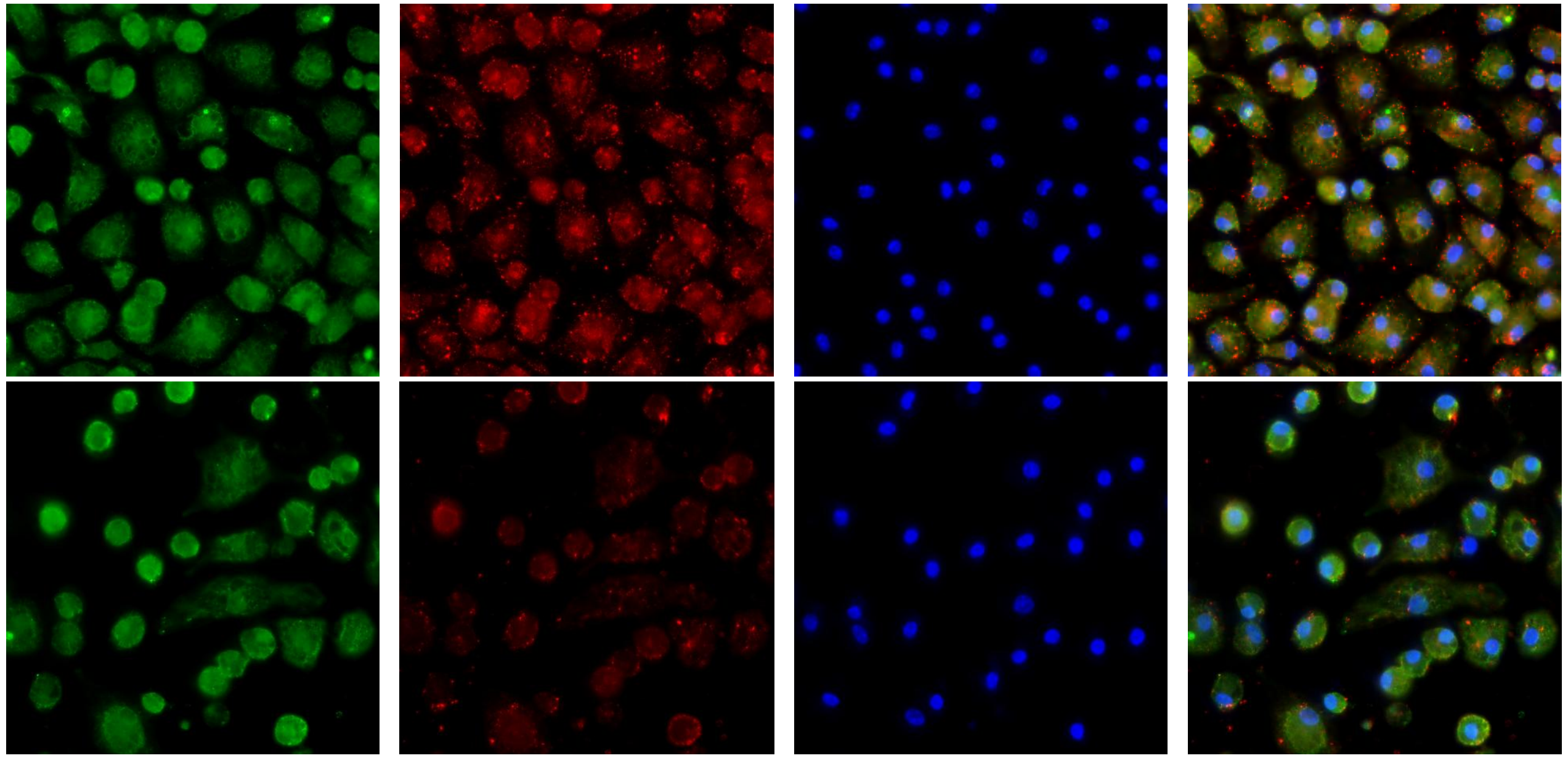

Figure S3A

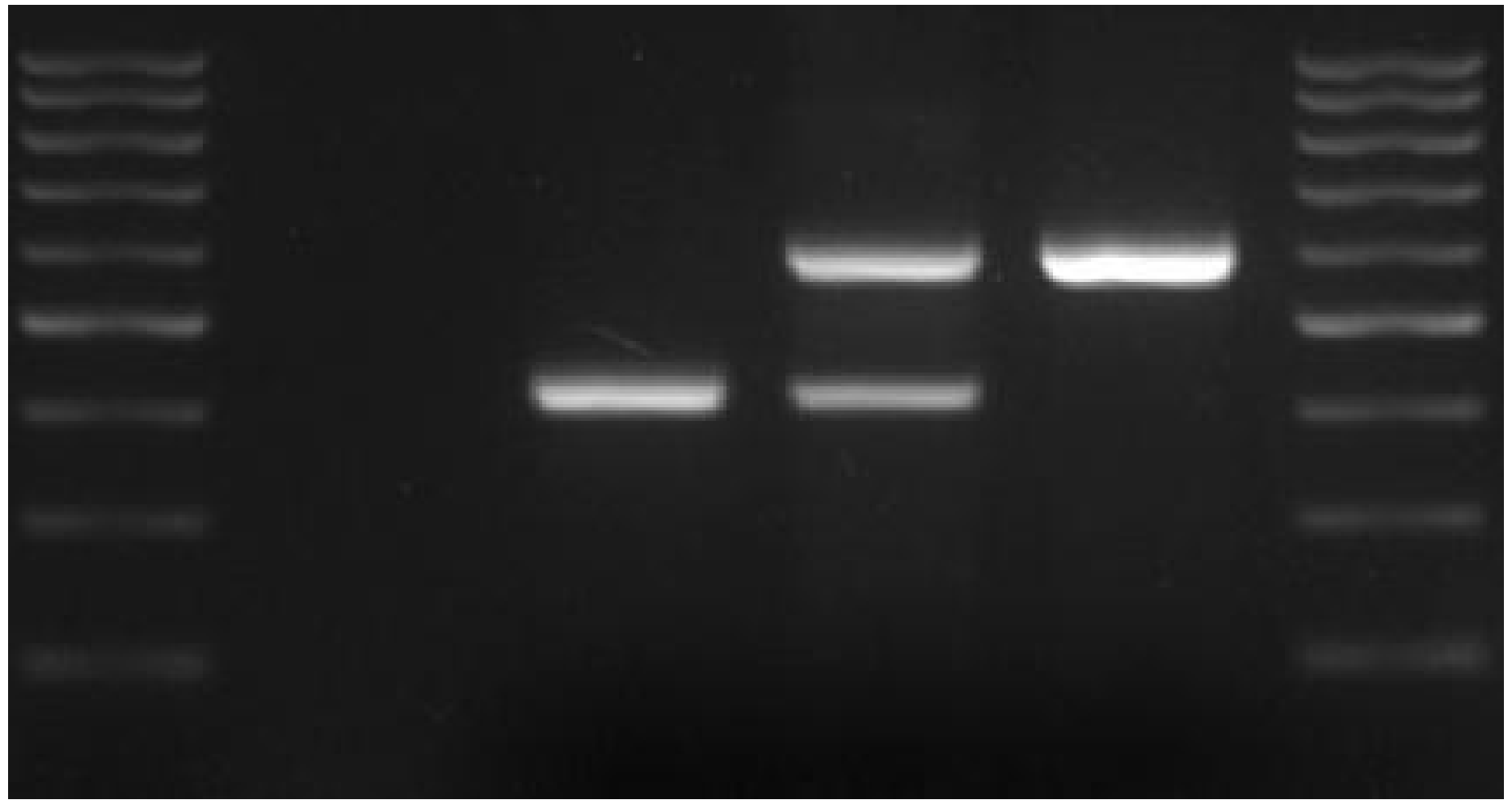

Figure S6B

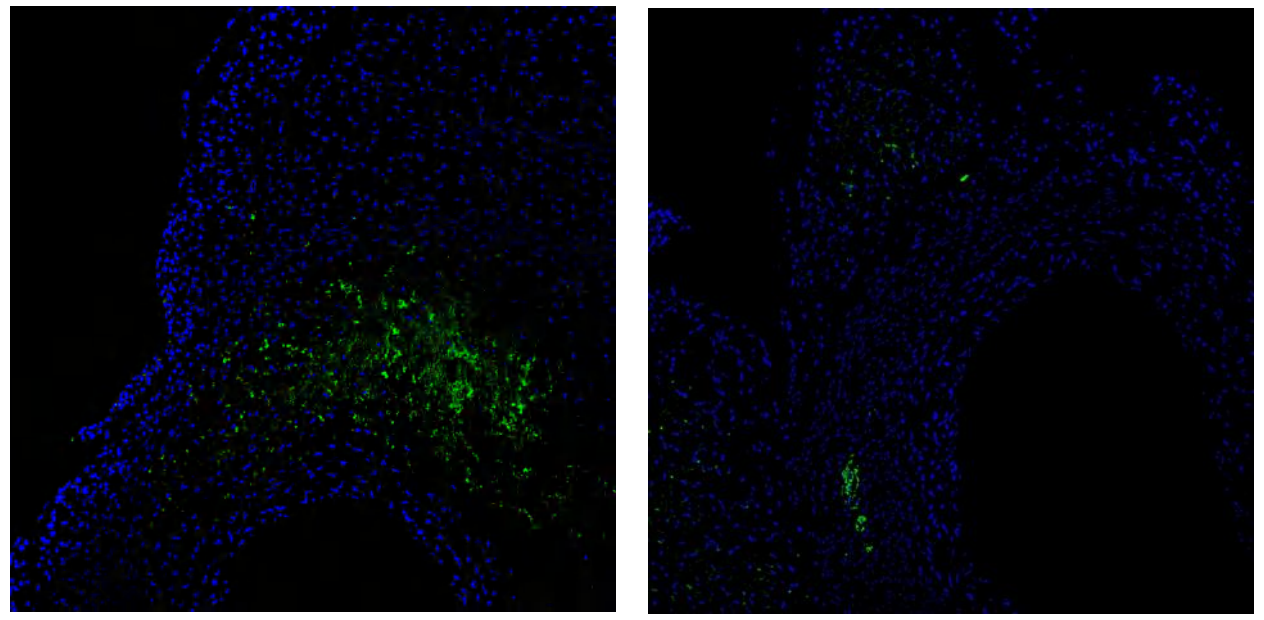

Figure S7A

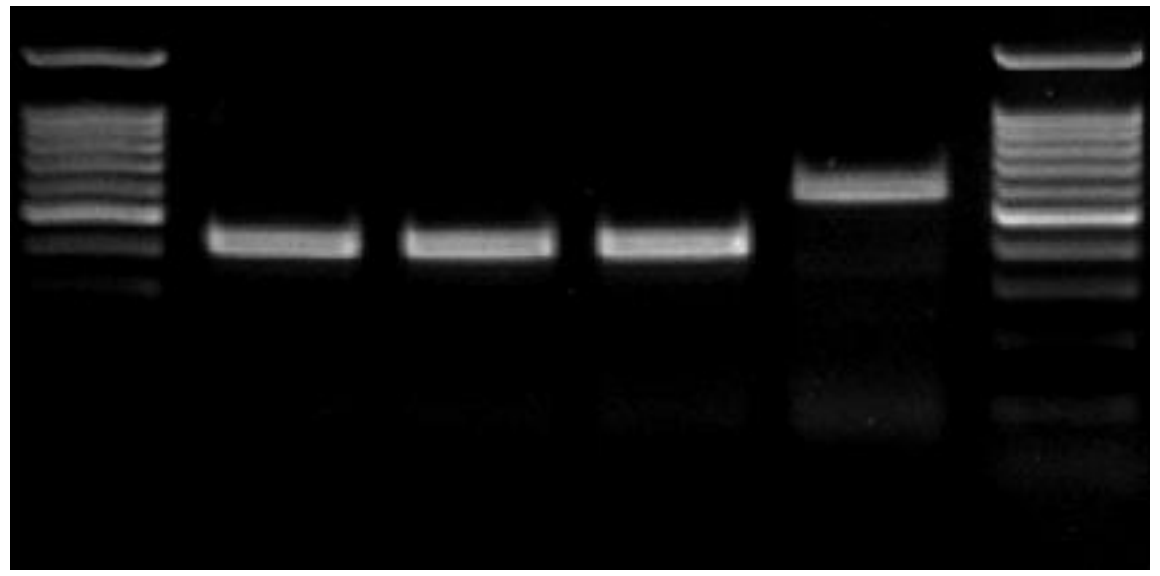

Figure S7C

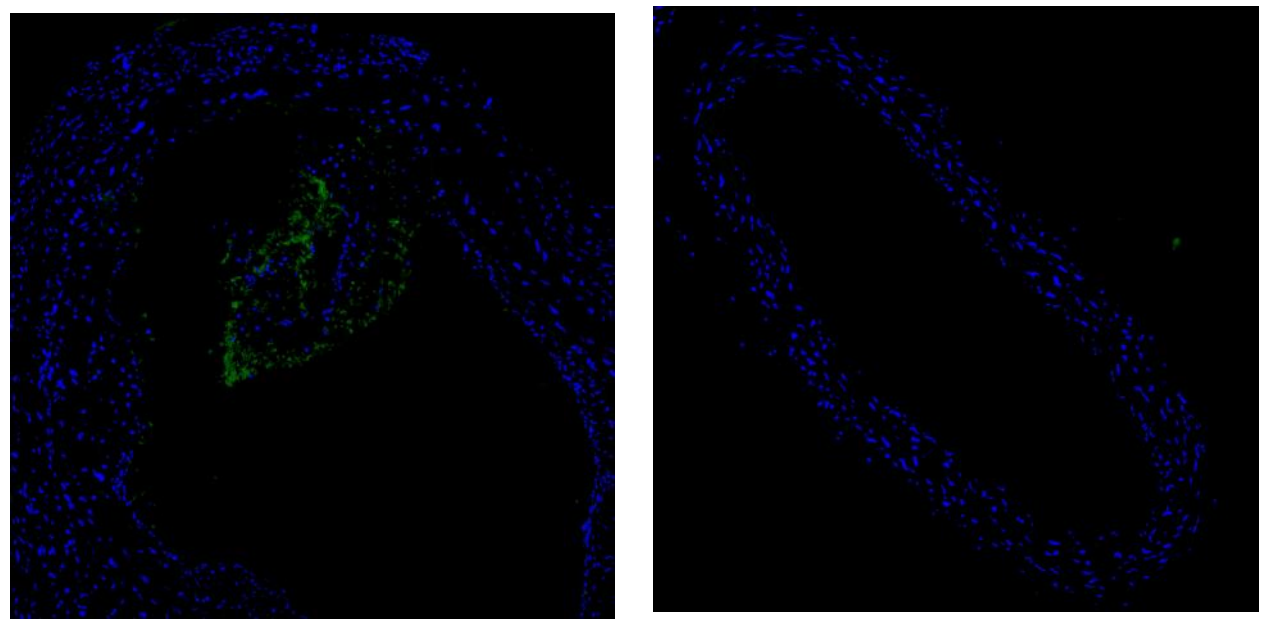

Figure S8B

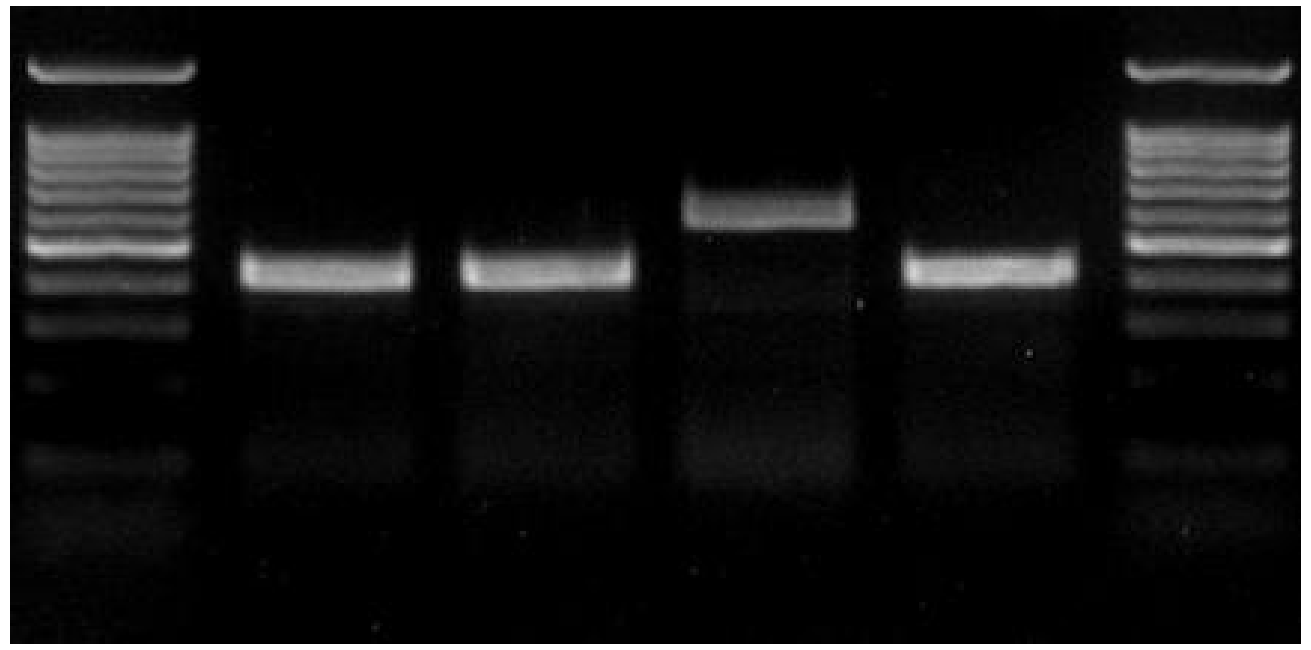

Figure S8F

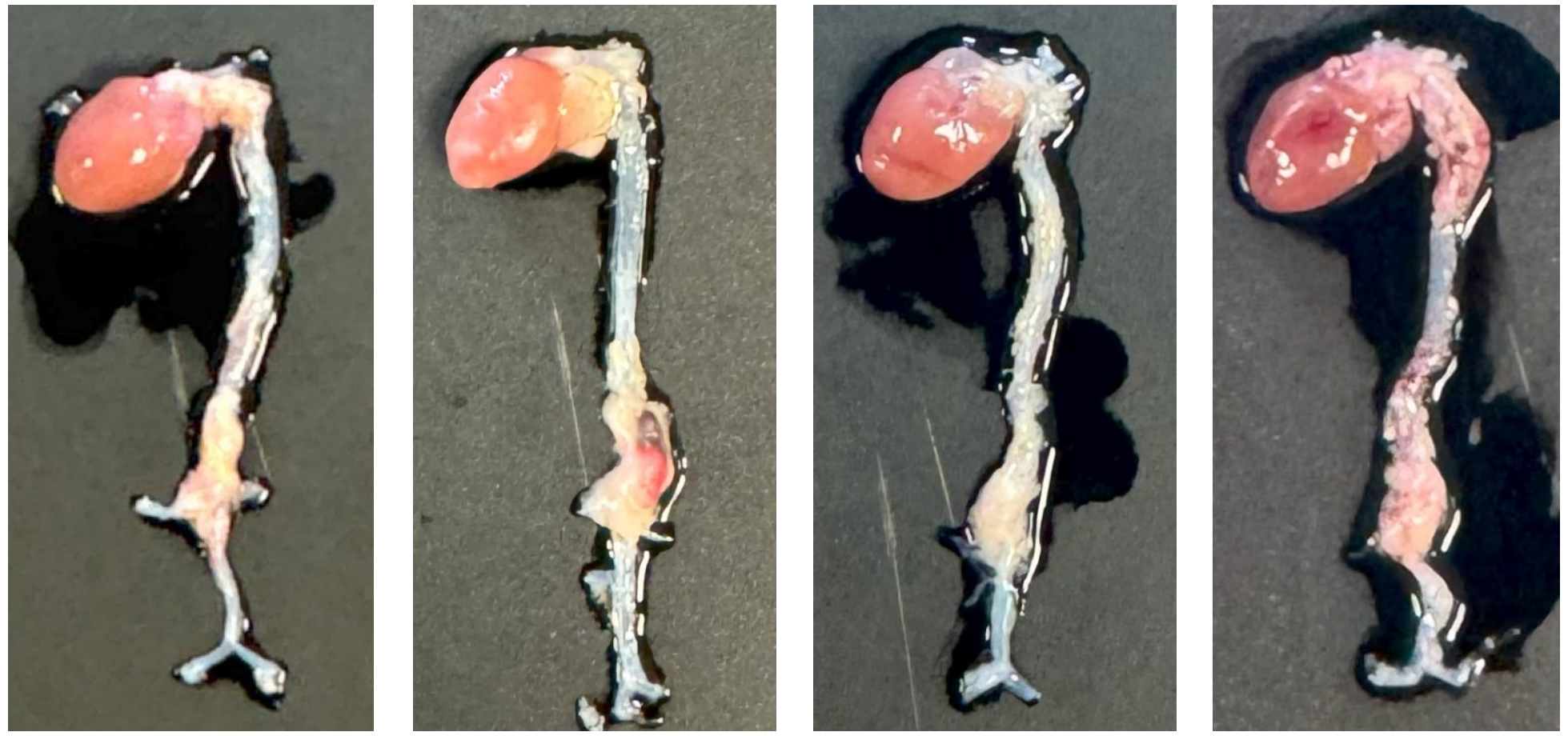

Figure S8J

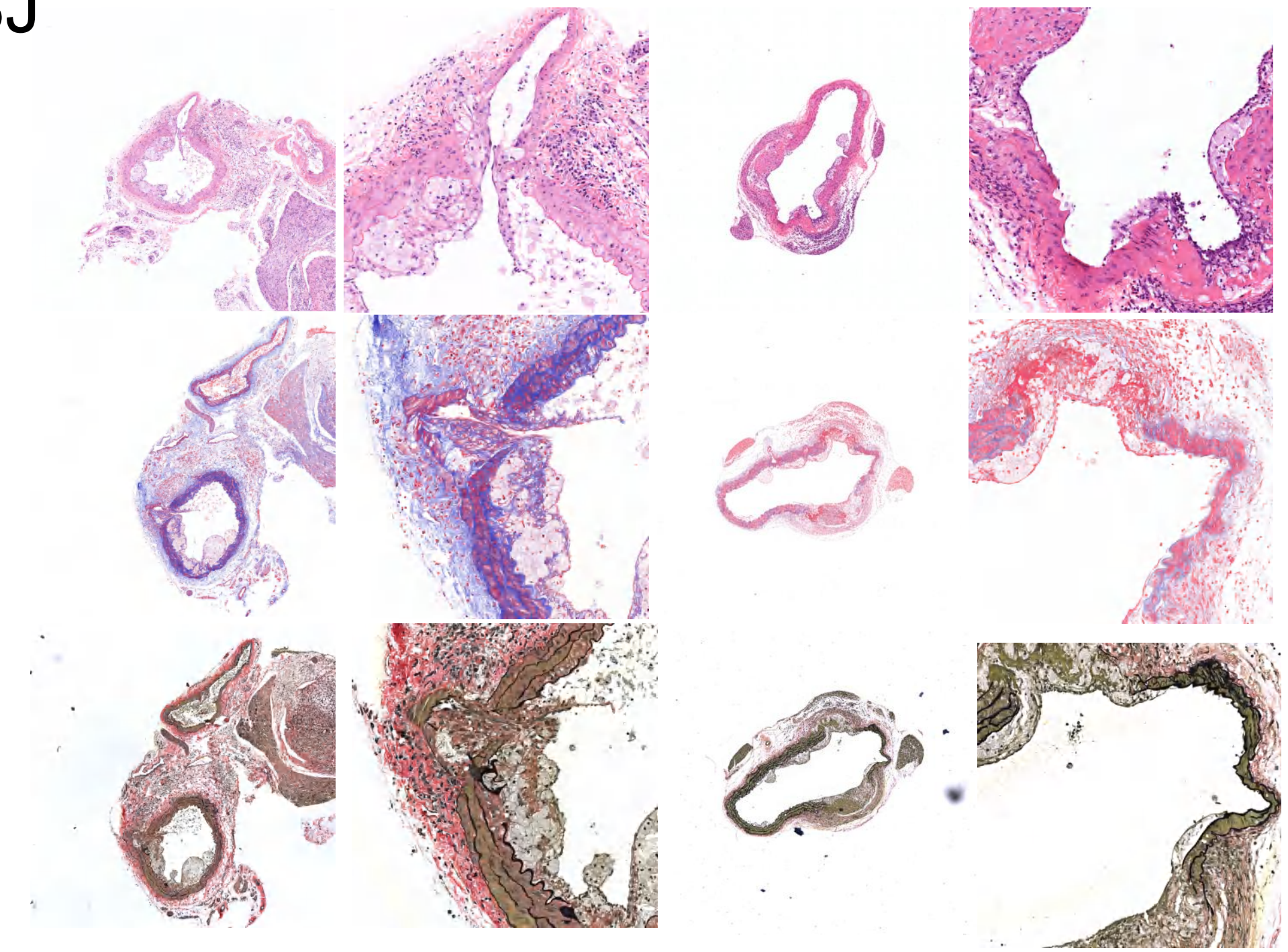

Figure S9B

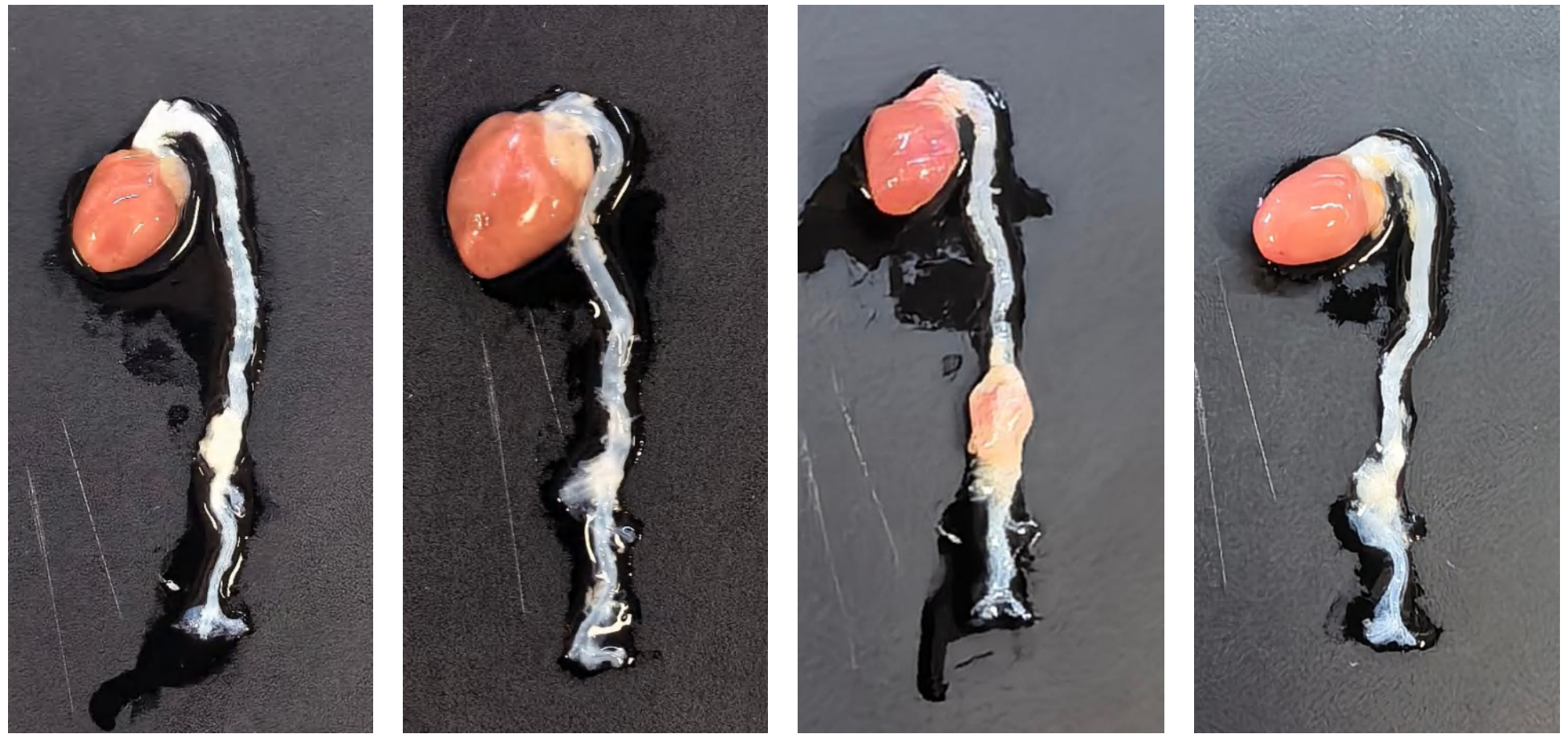

Figure S9E

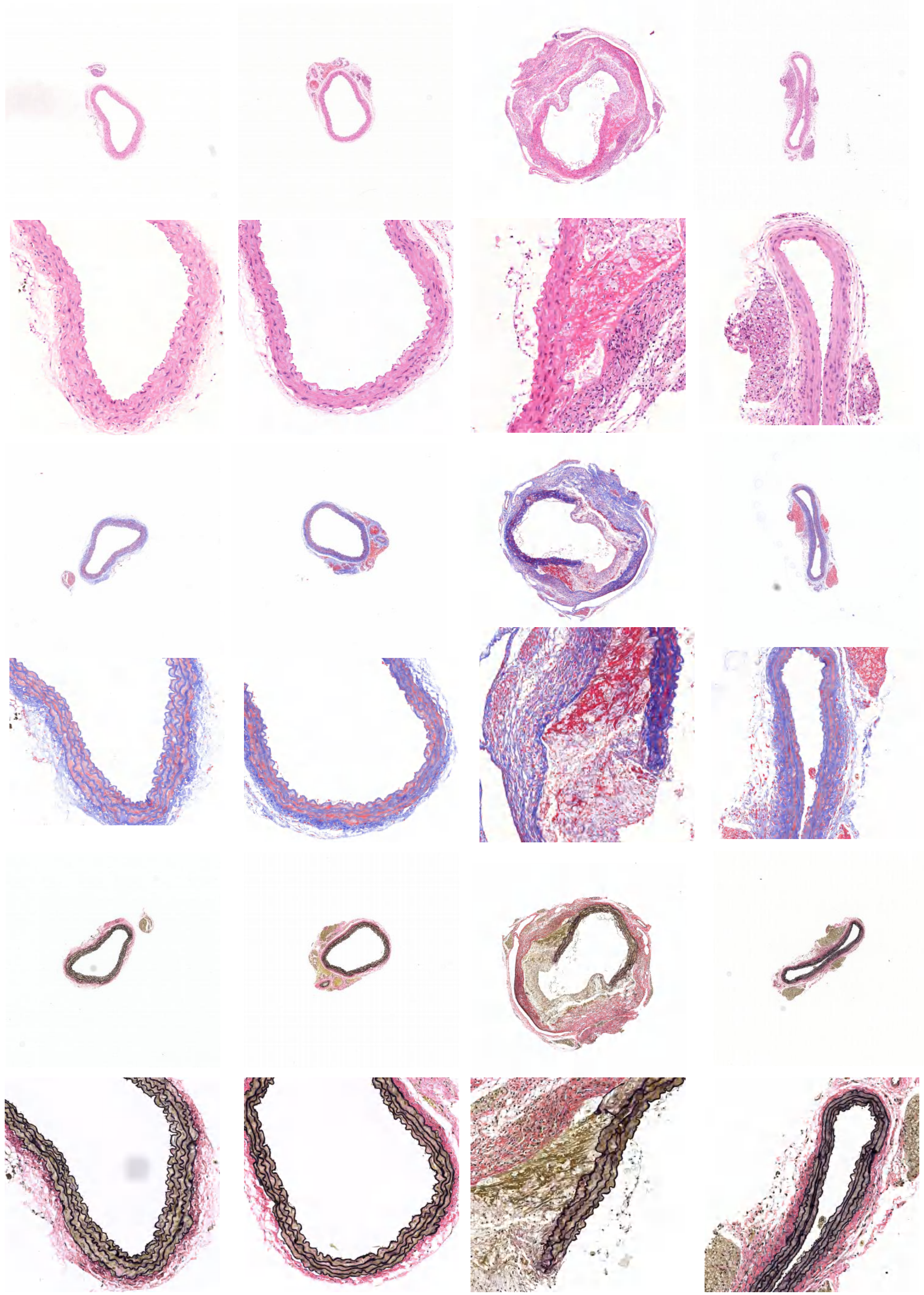

Figure S14C

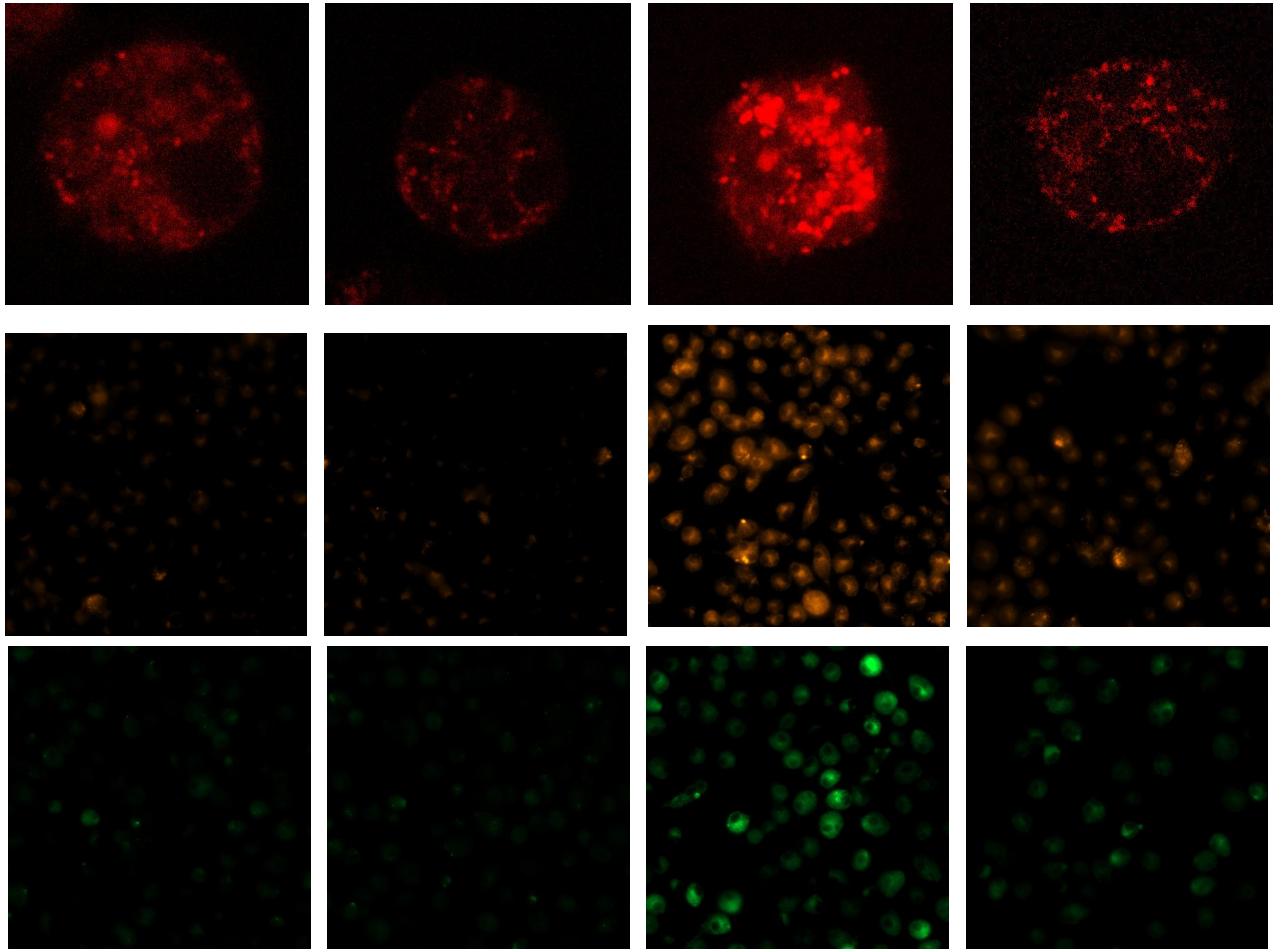

Figure S16B

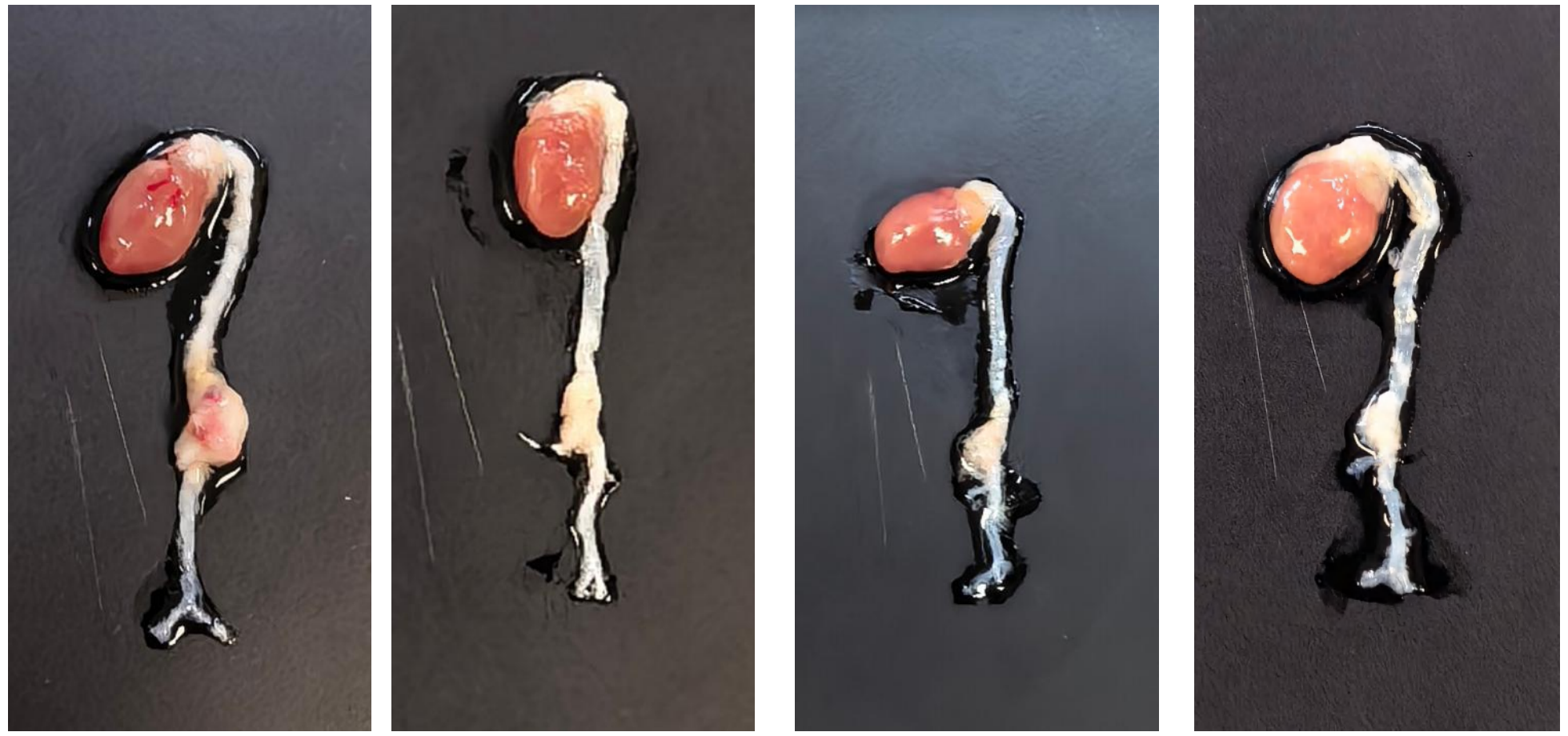

Figure S16D

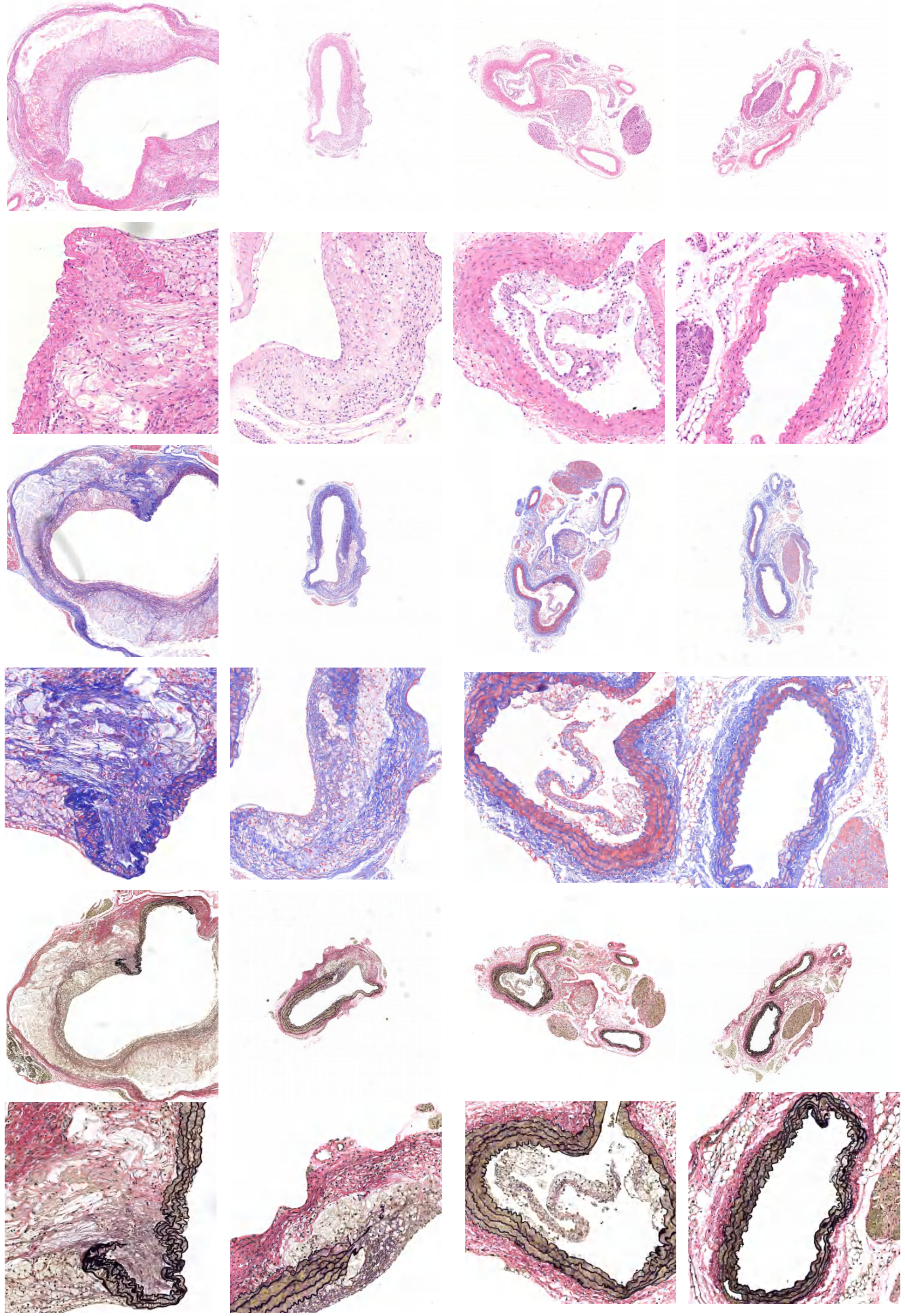

Figure S18A

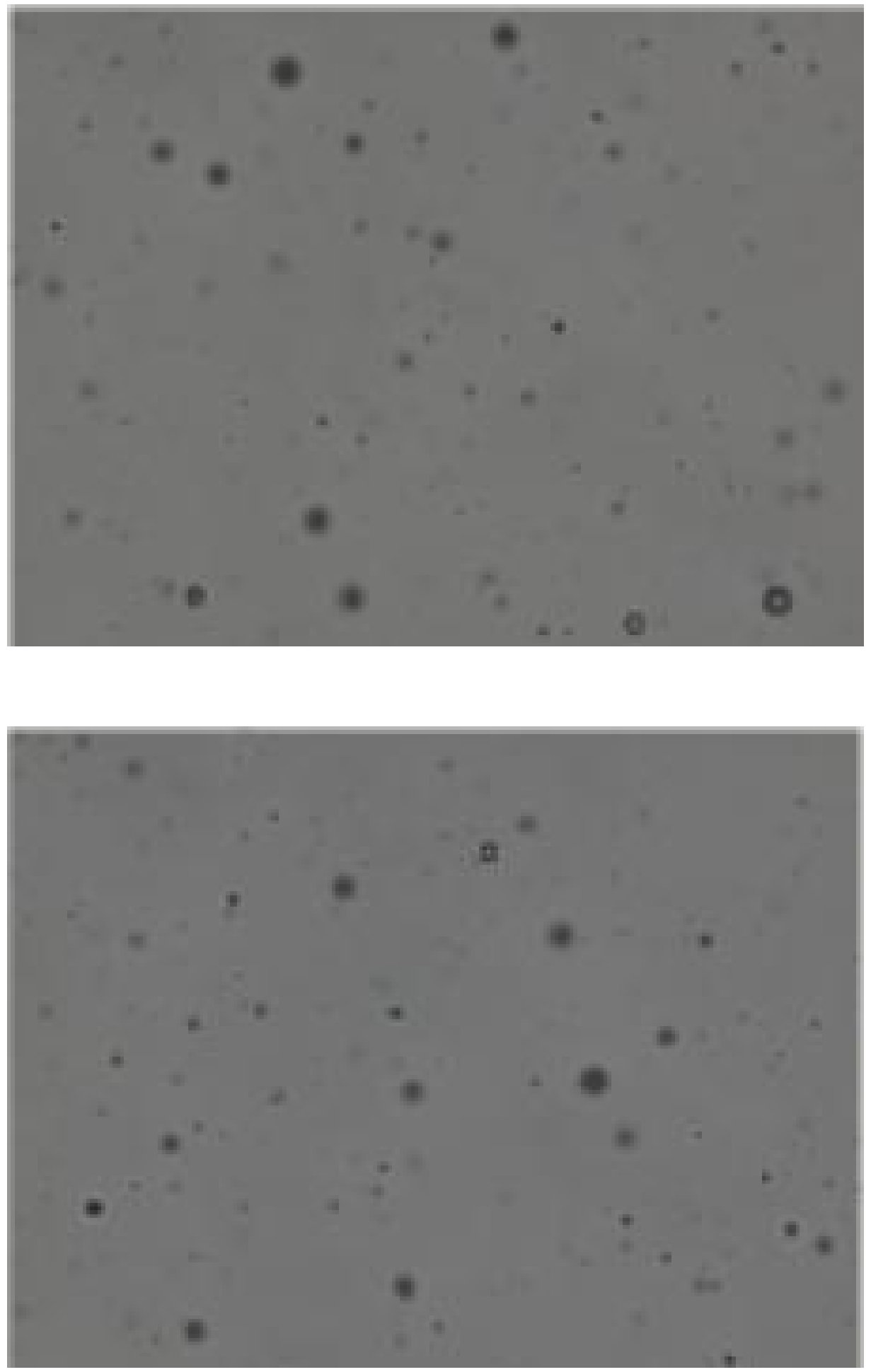

Figure S18E

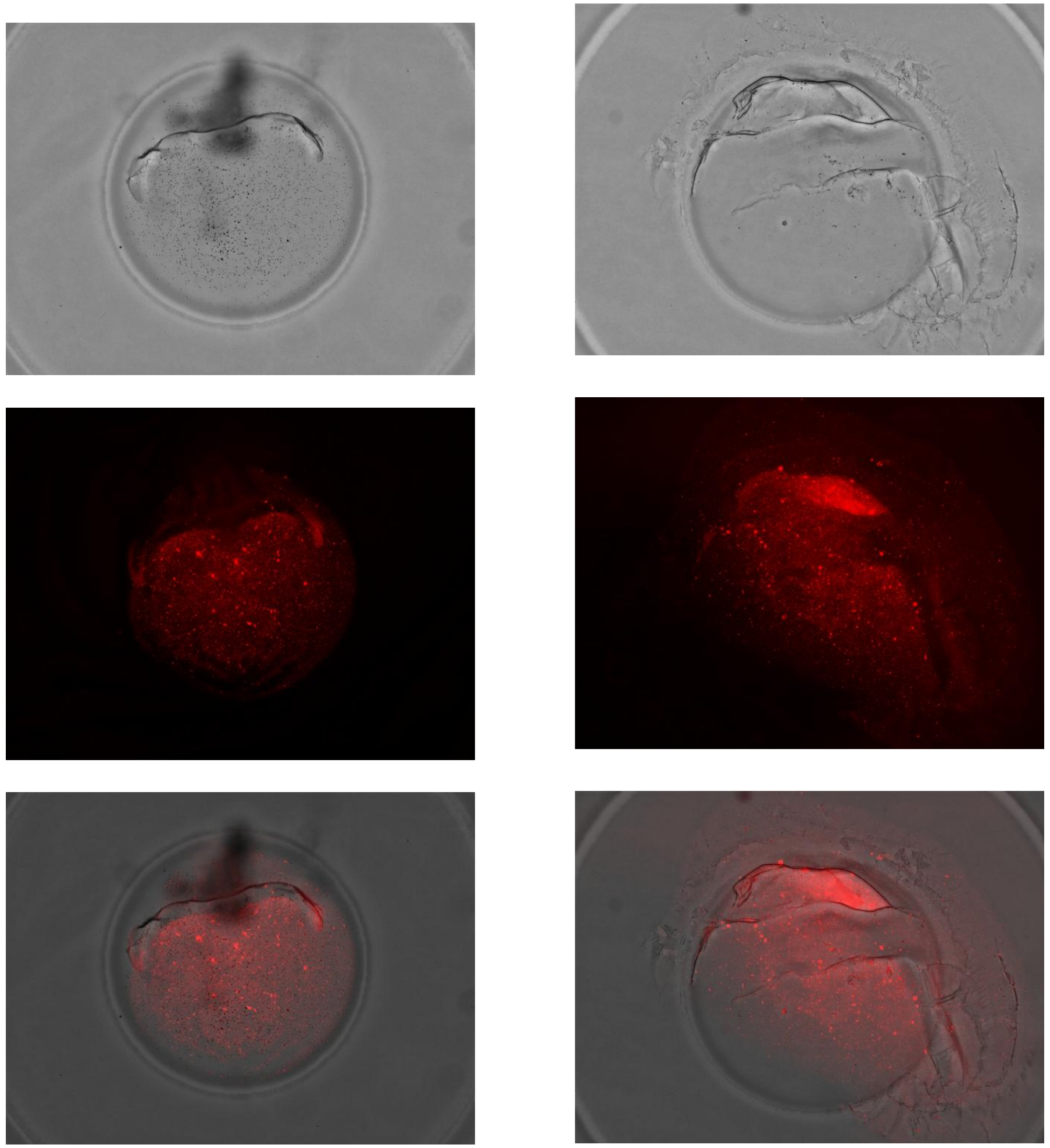

Figure S18F

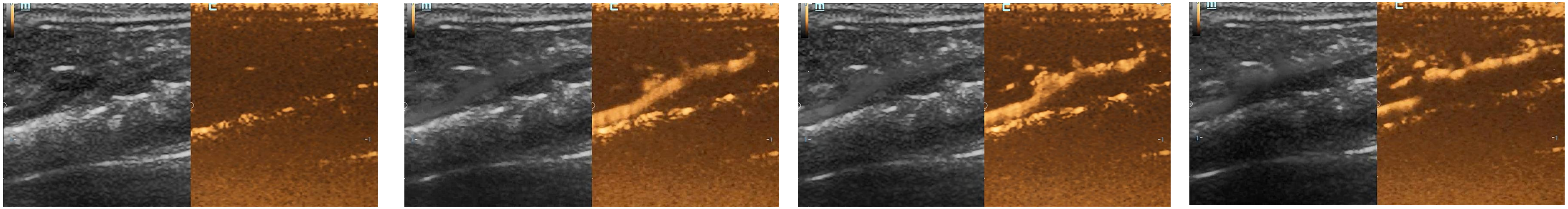

Figure S18G

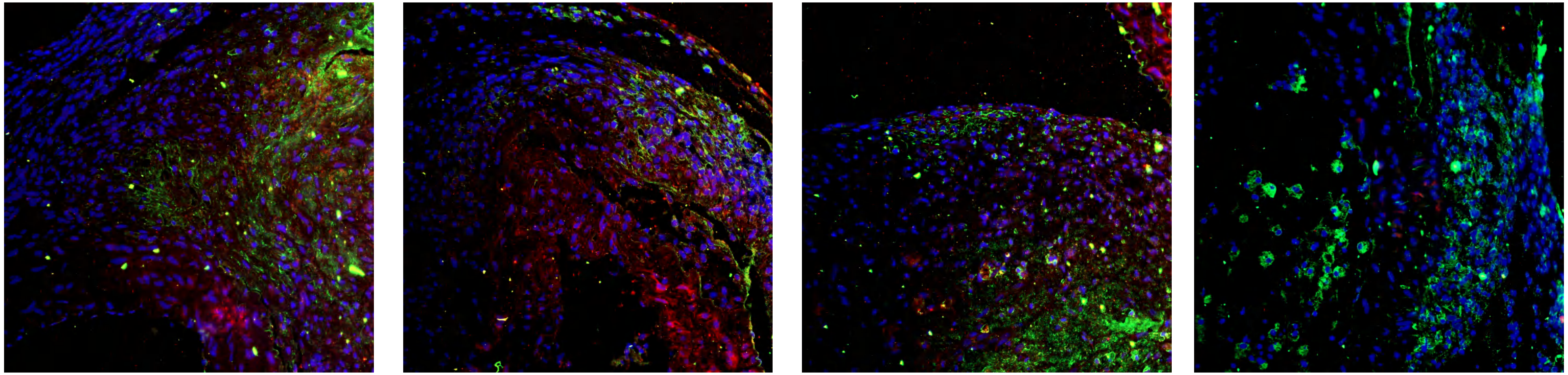

Figure S19C

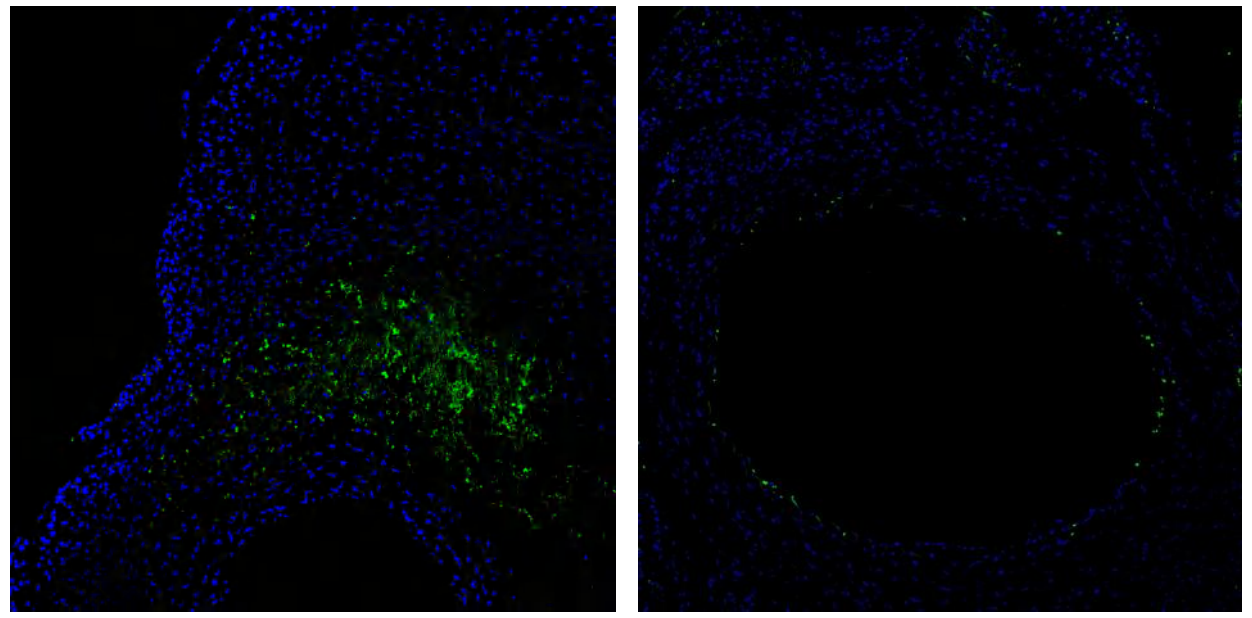

Figure S20B

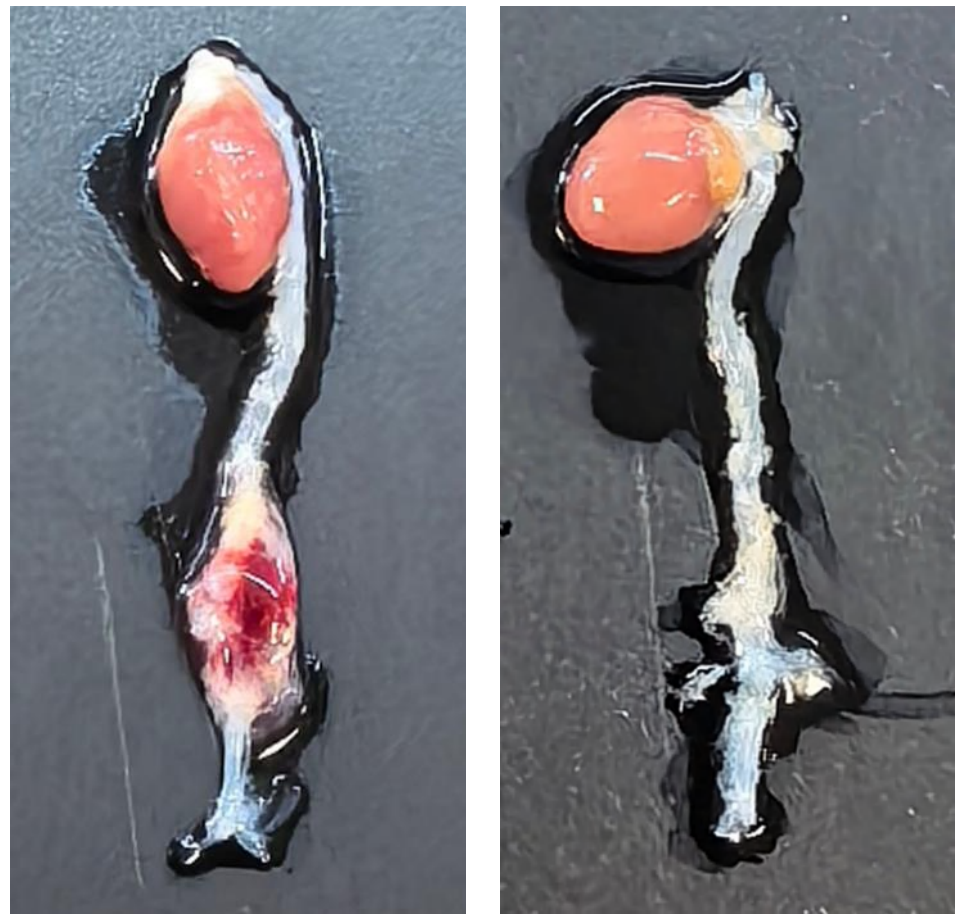

Figure S20D

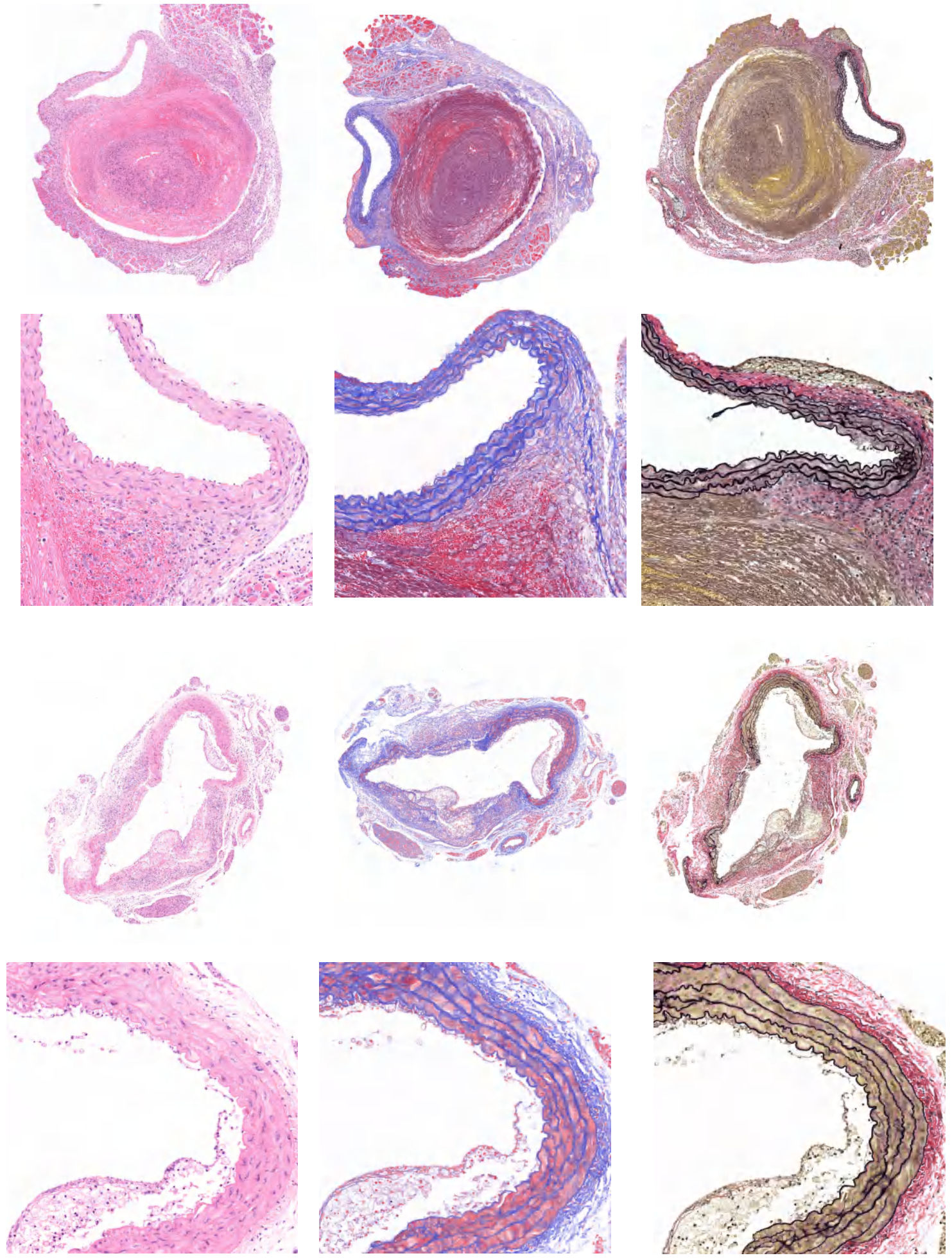

Figure S21A

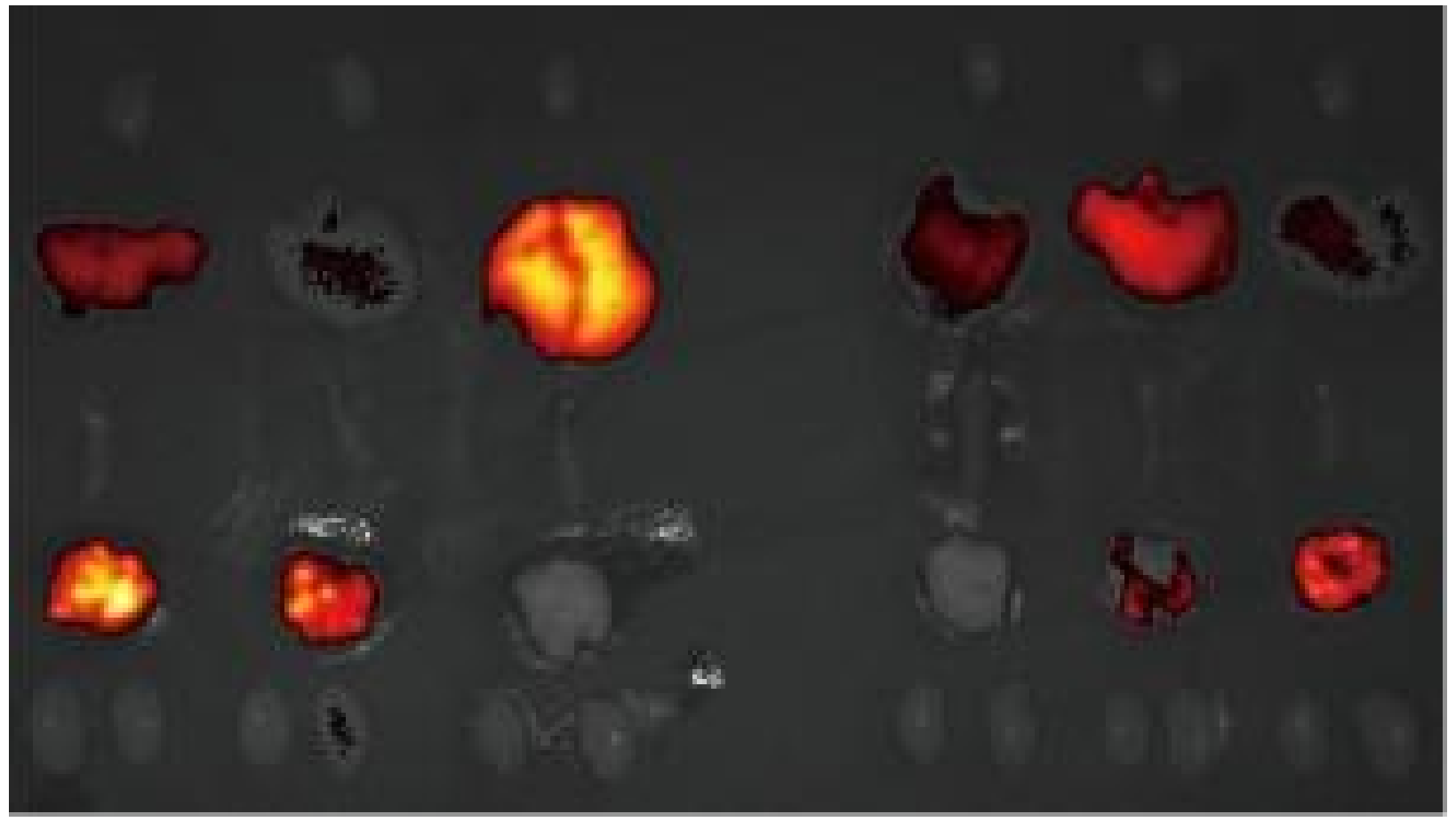

Figure S22A

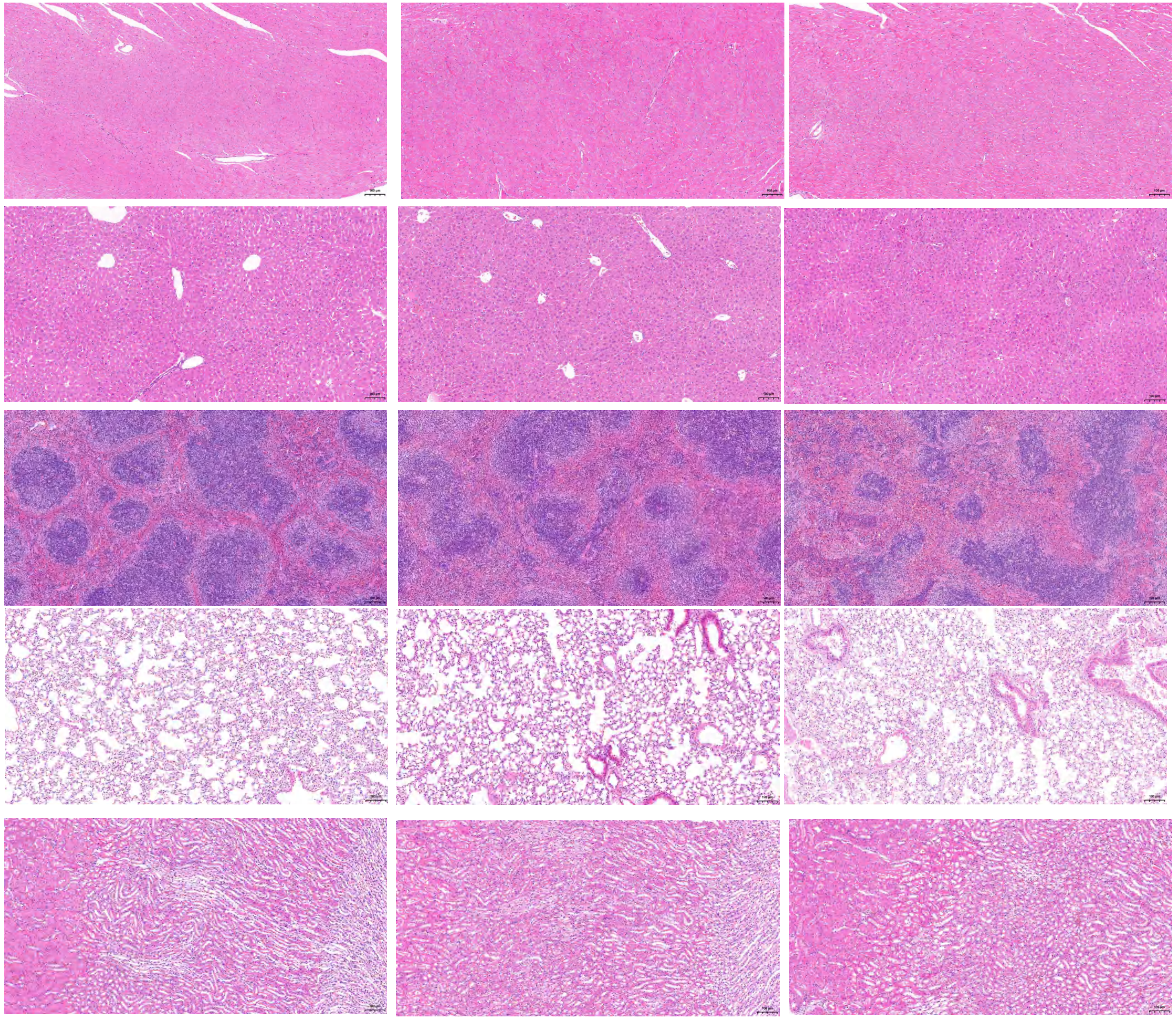

Figure S22B

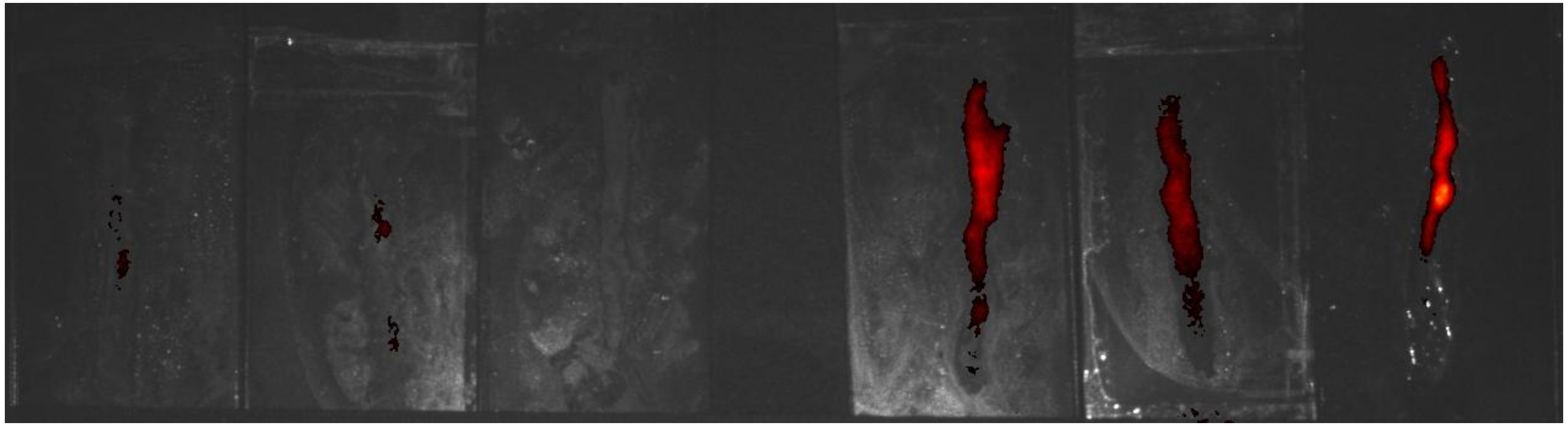

Figure S22C

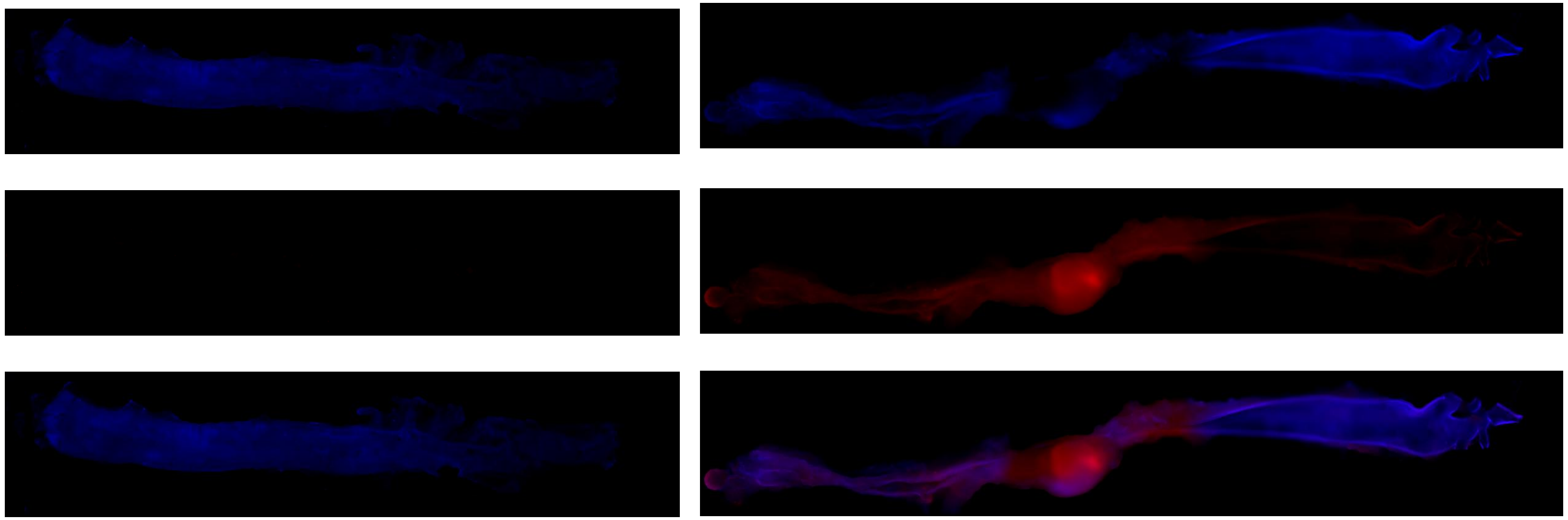

Uncropped Blot

Figure 1

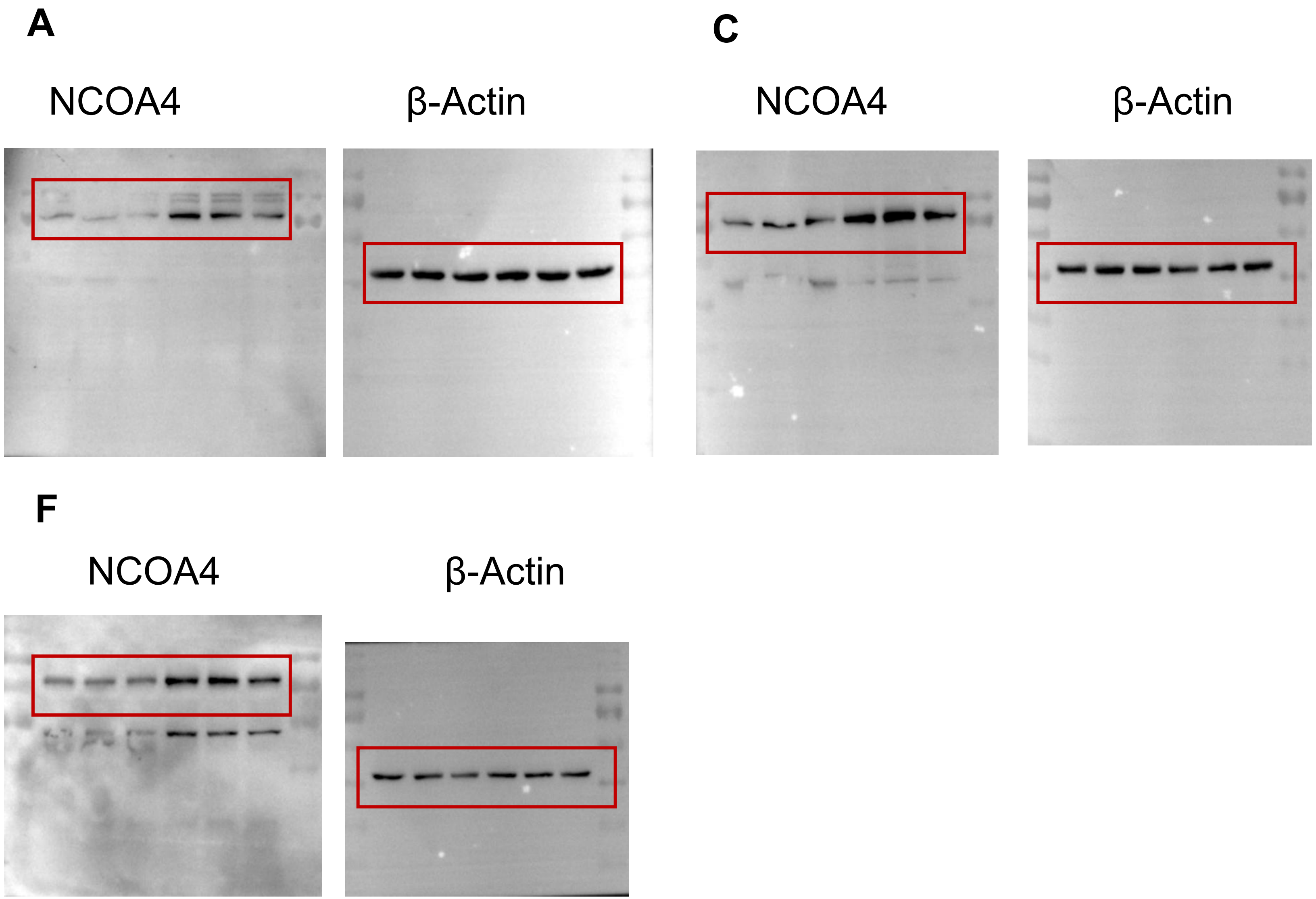

Figure 4

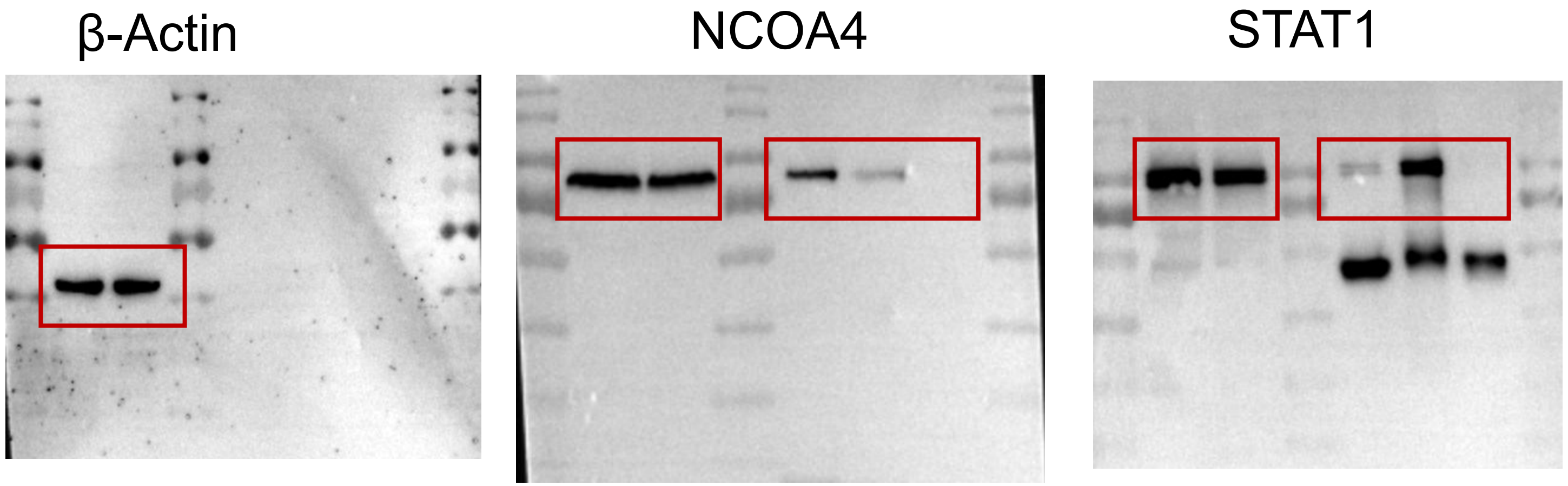

Figure 5

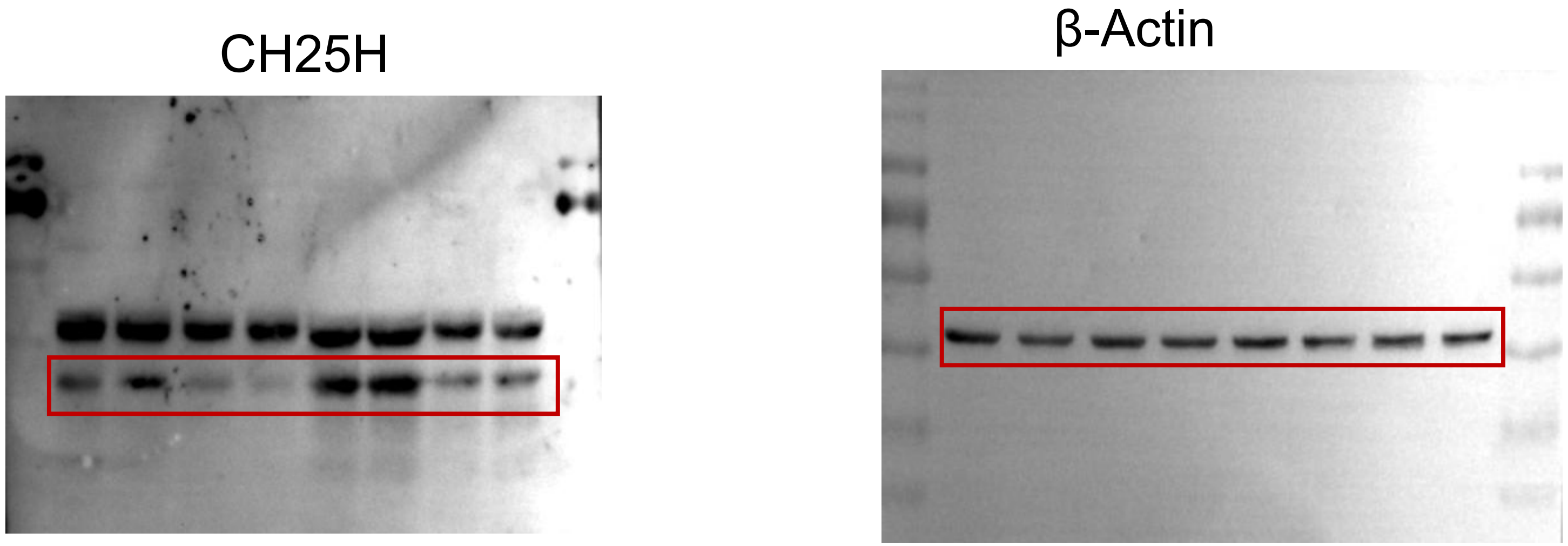

Figure 6

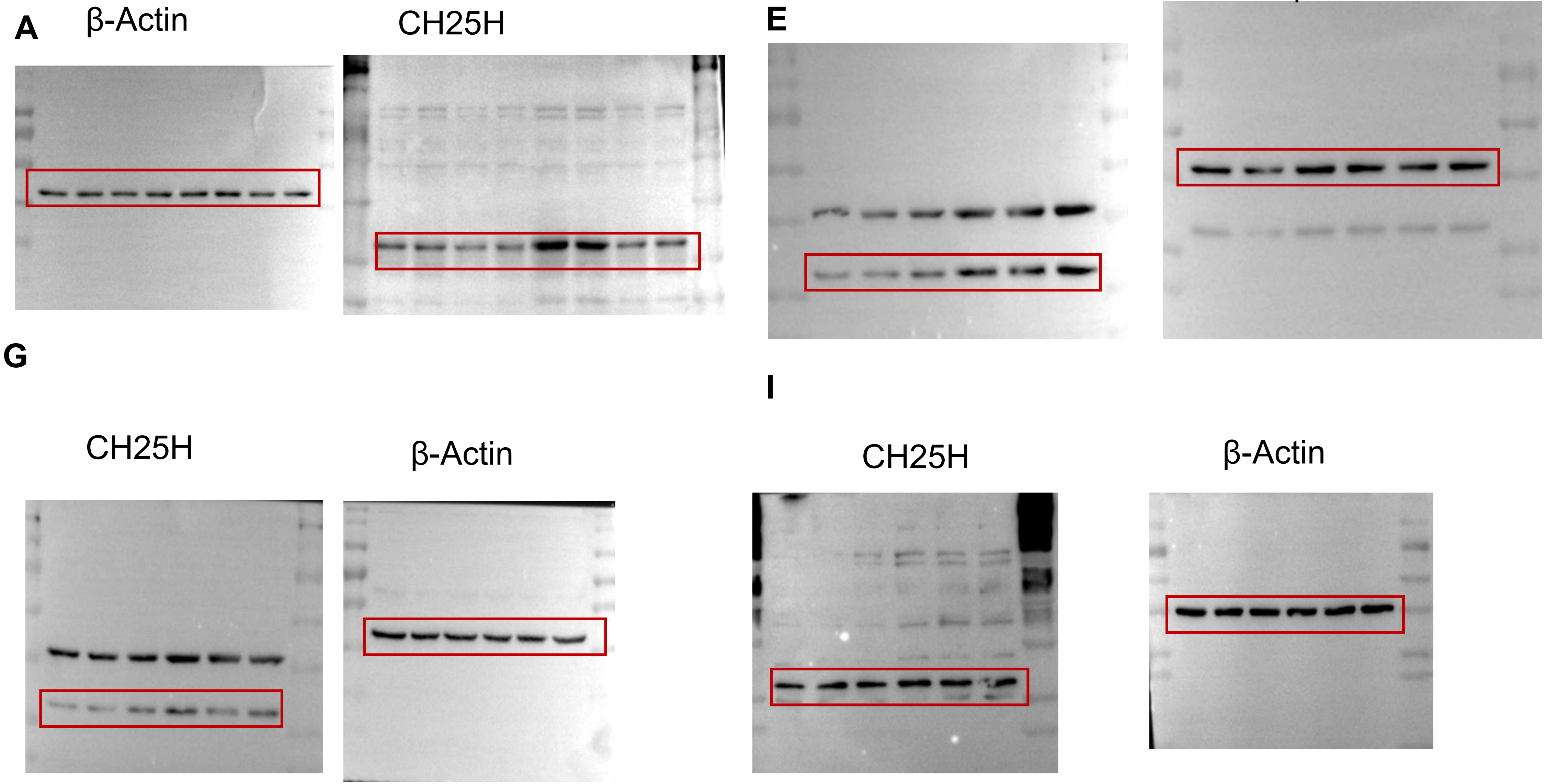

Figure S1

C

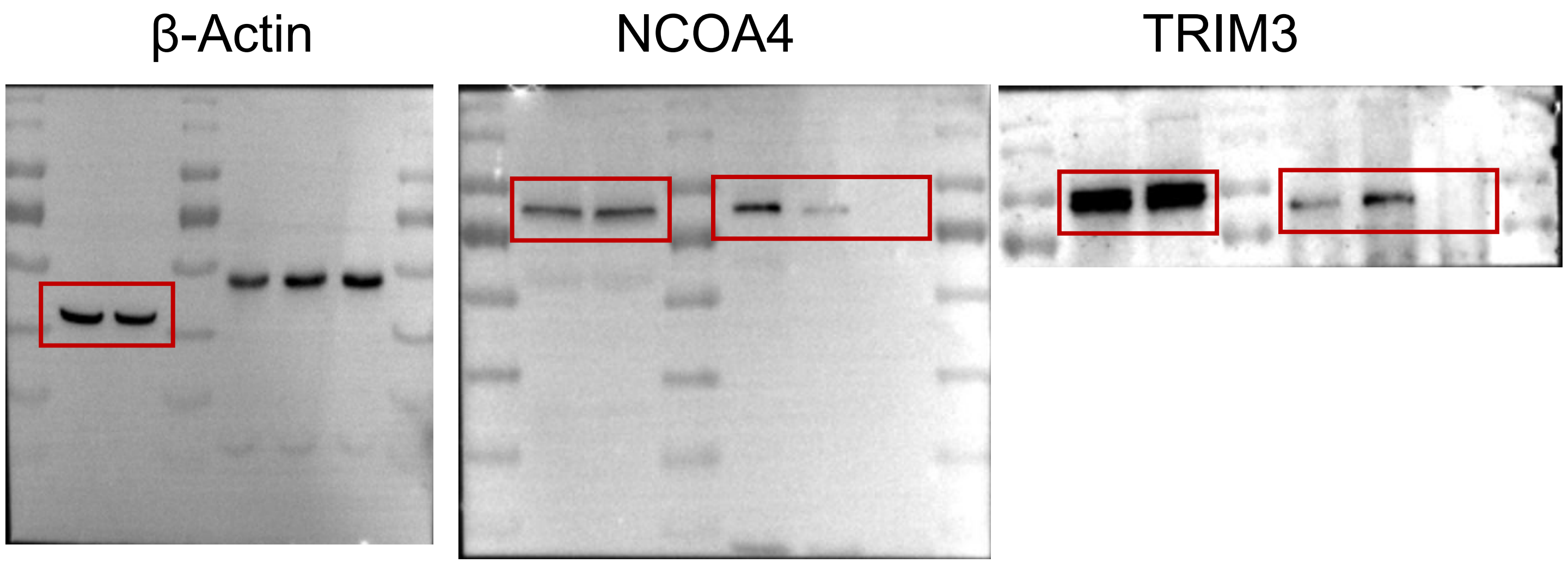

D

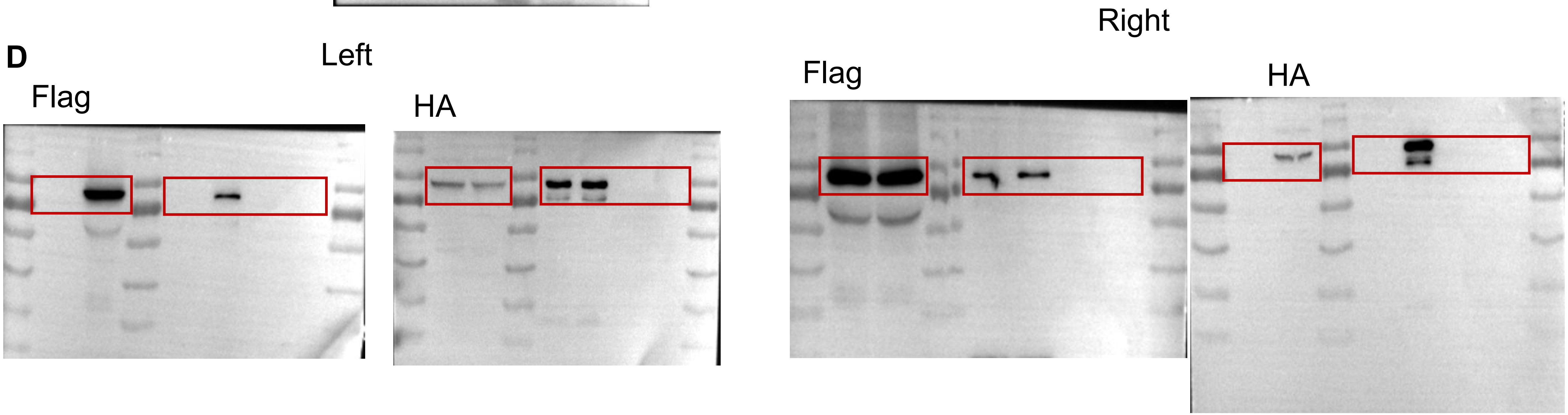

Figure S1

E

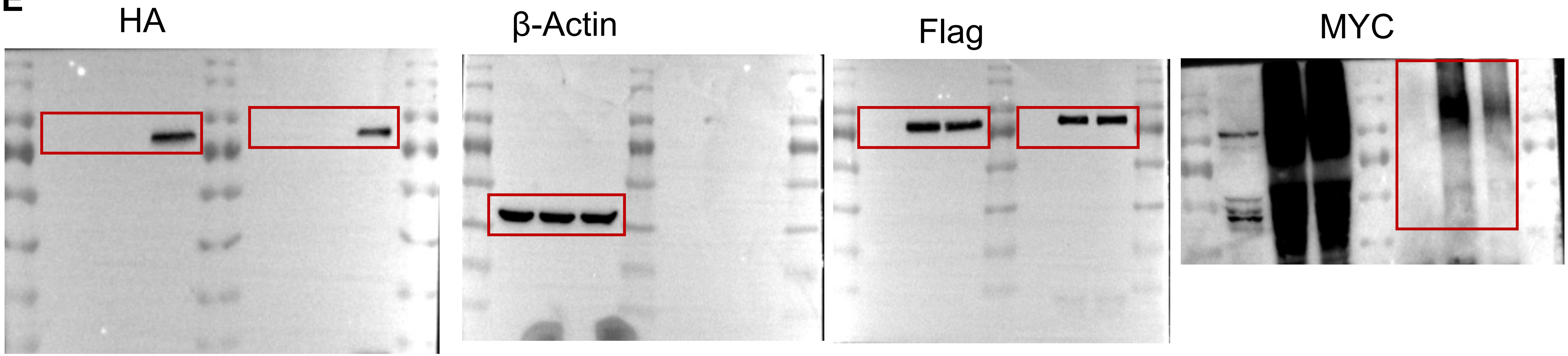

F

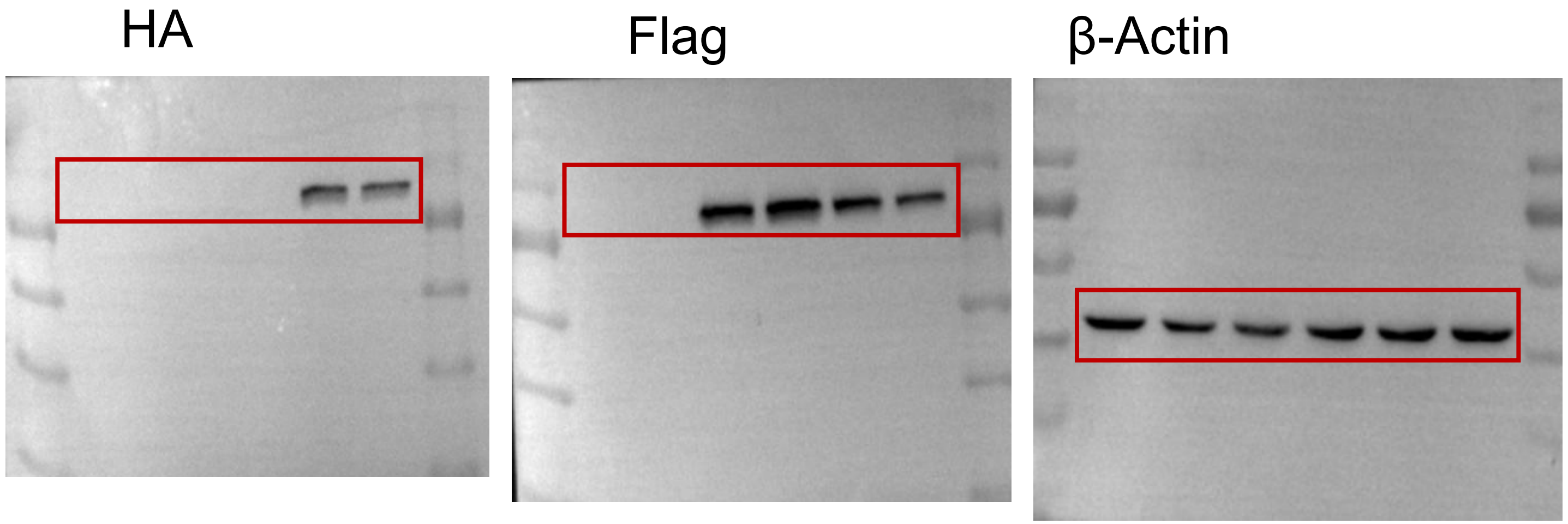

Figure S2

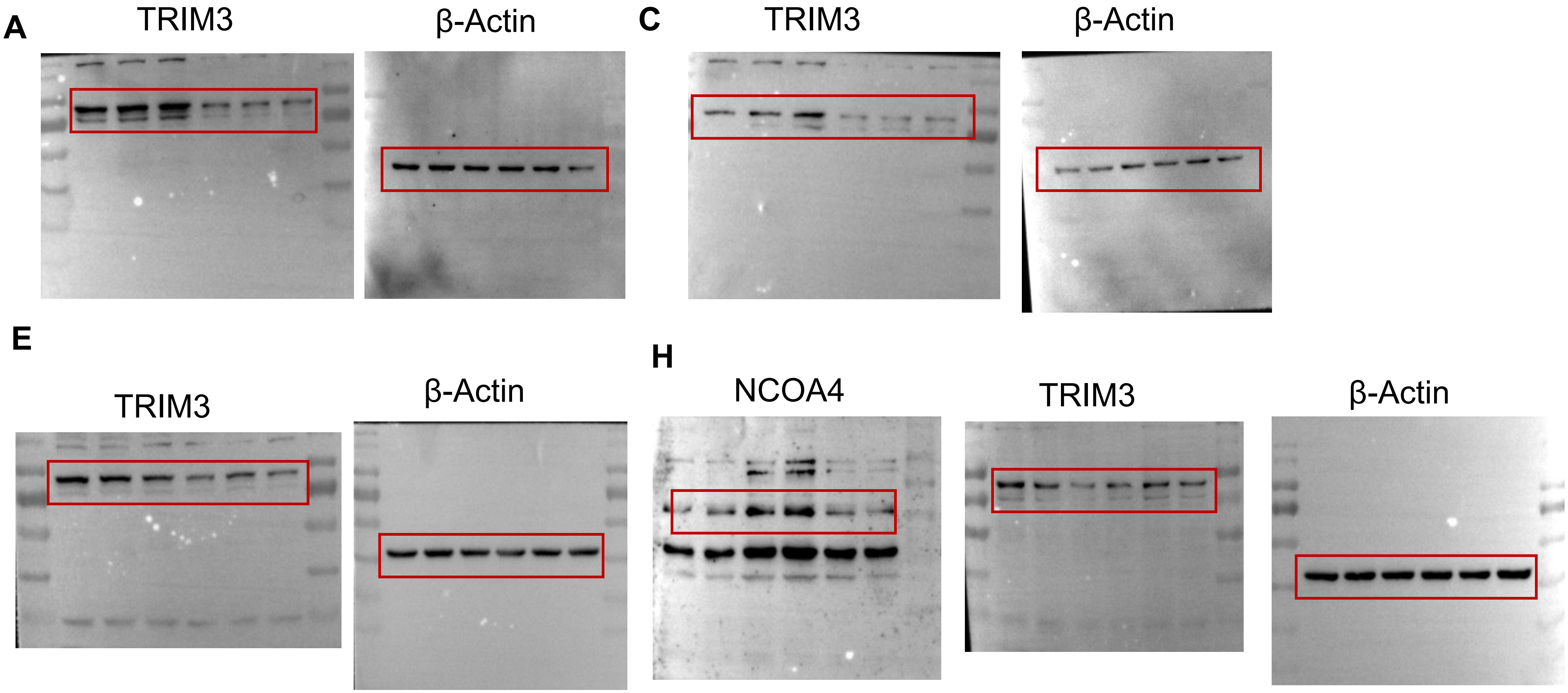
A
TRIM3
β-Actin
C
TRIM3
β-Actin
E
TRIM3
β-Actin
H
NCOA4
TRIM3
β-Actin

Figure S3

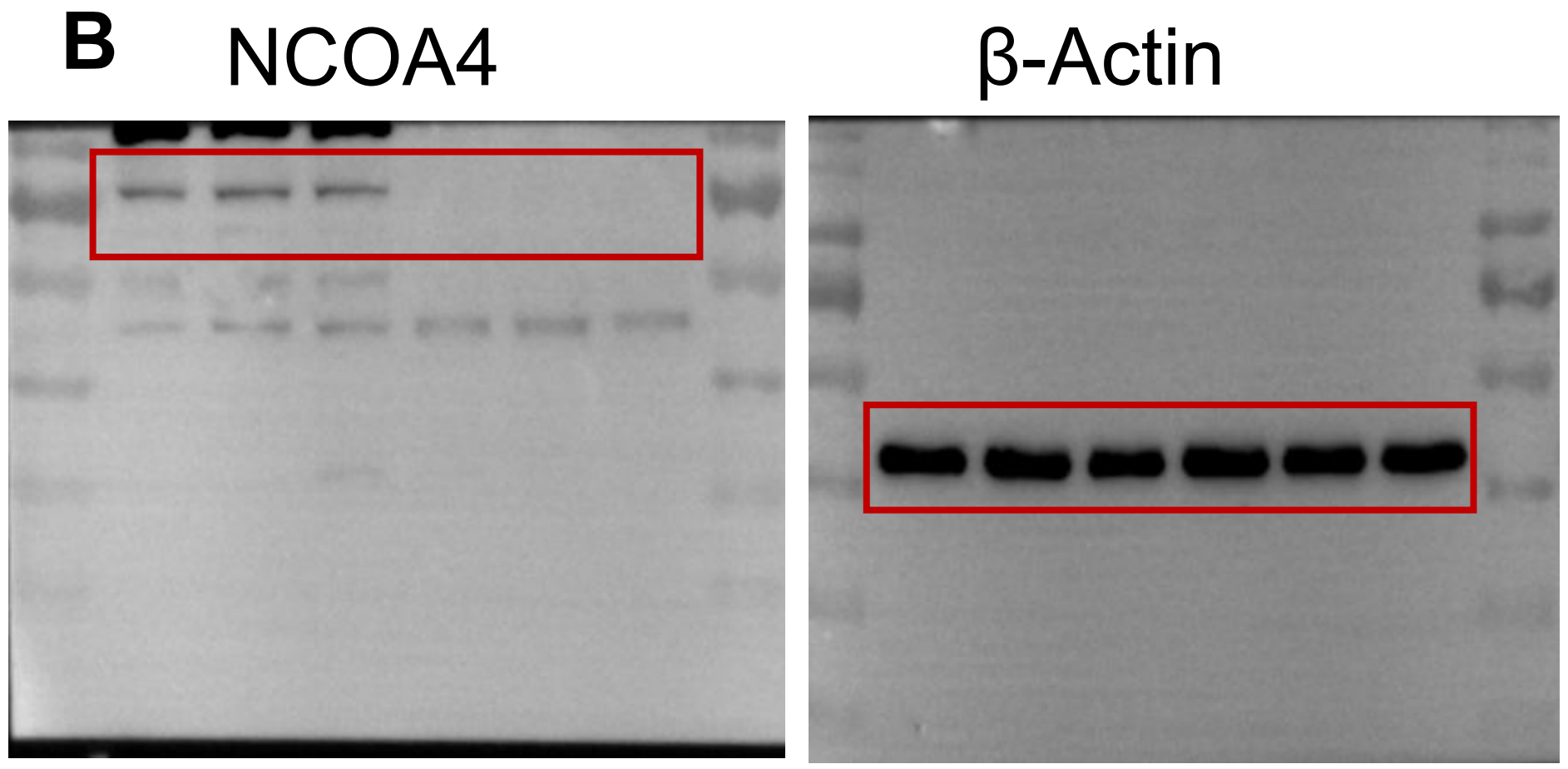

Figure S4

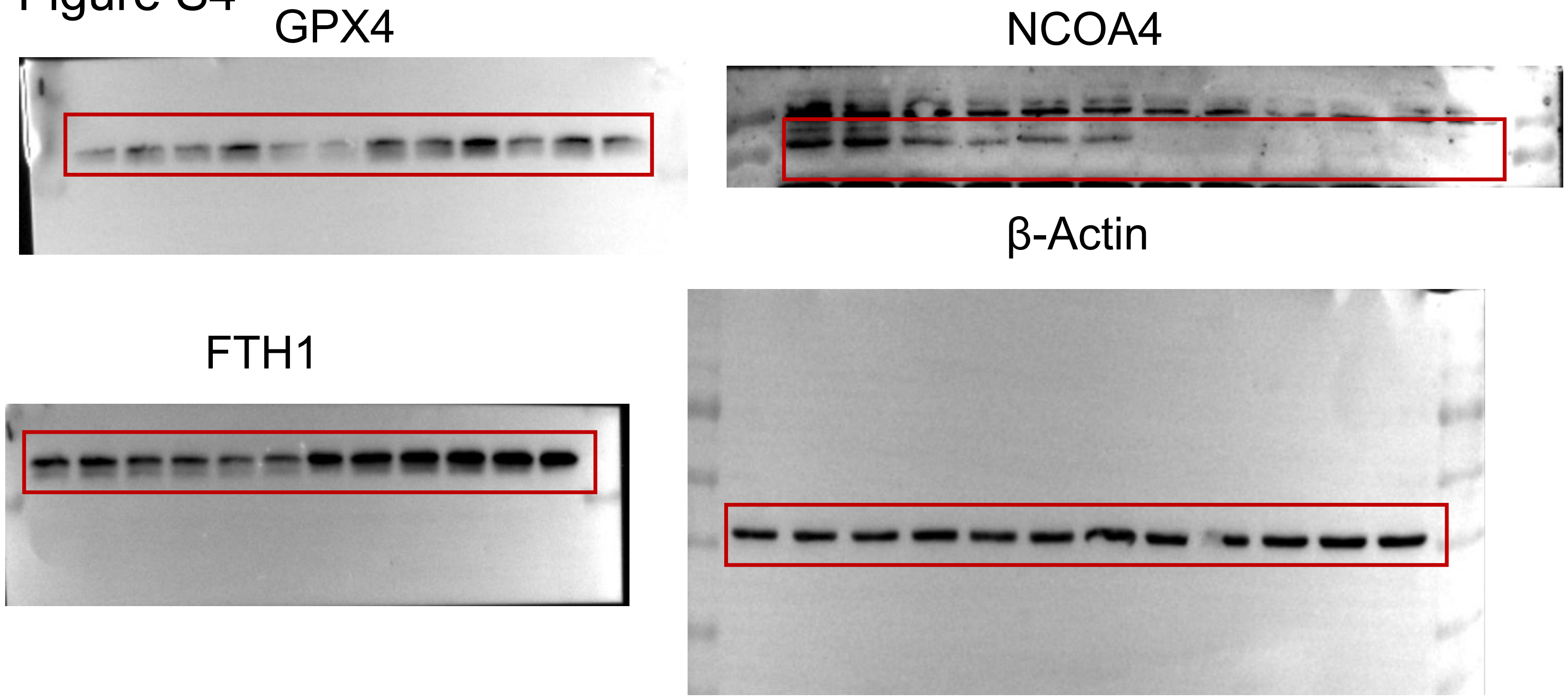

Figure S6

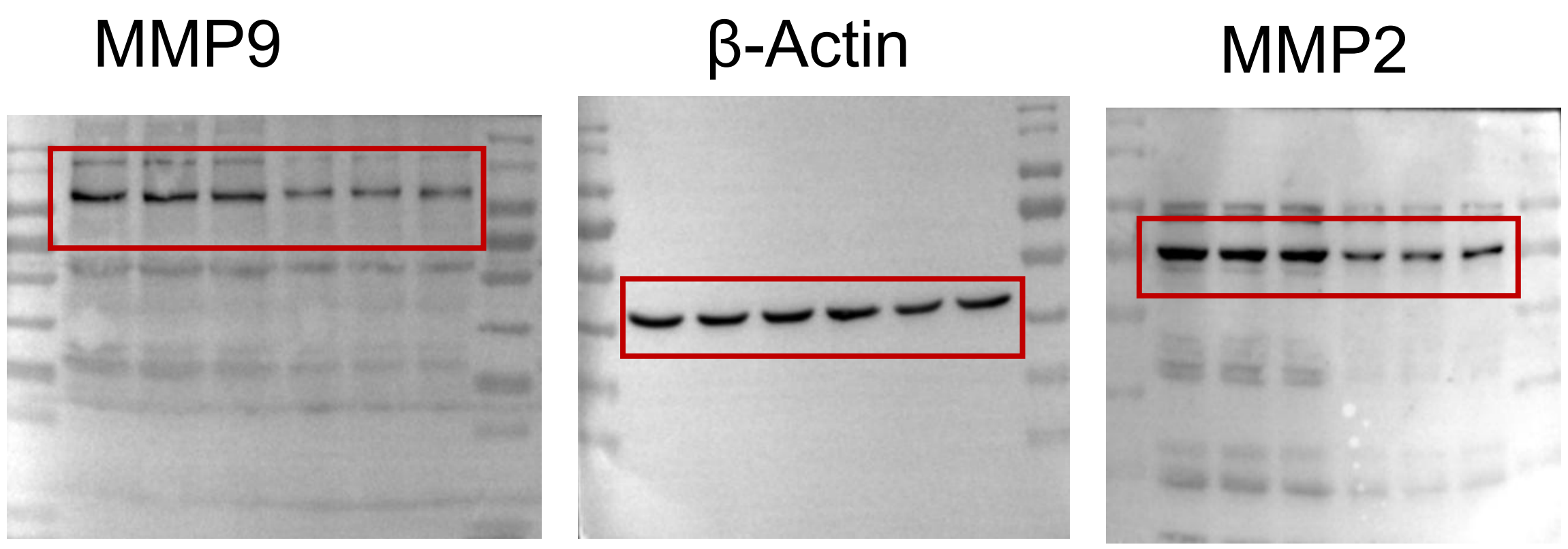

Figure S7

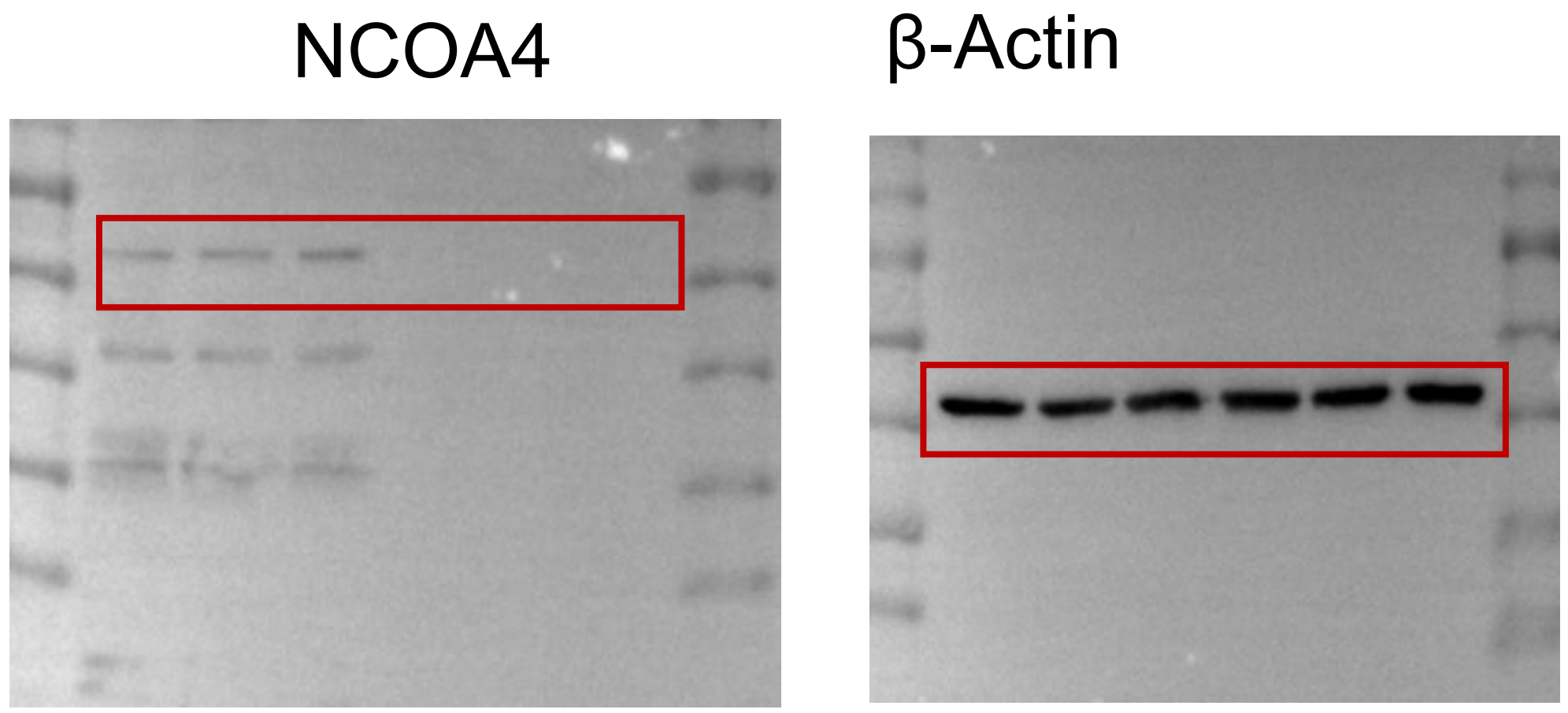

Figure S8

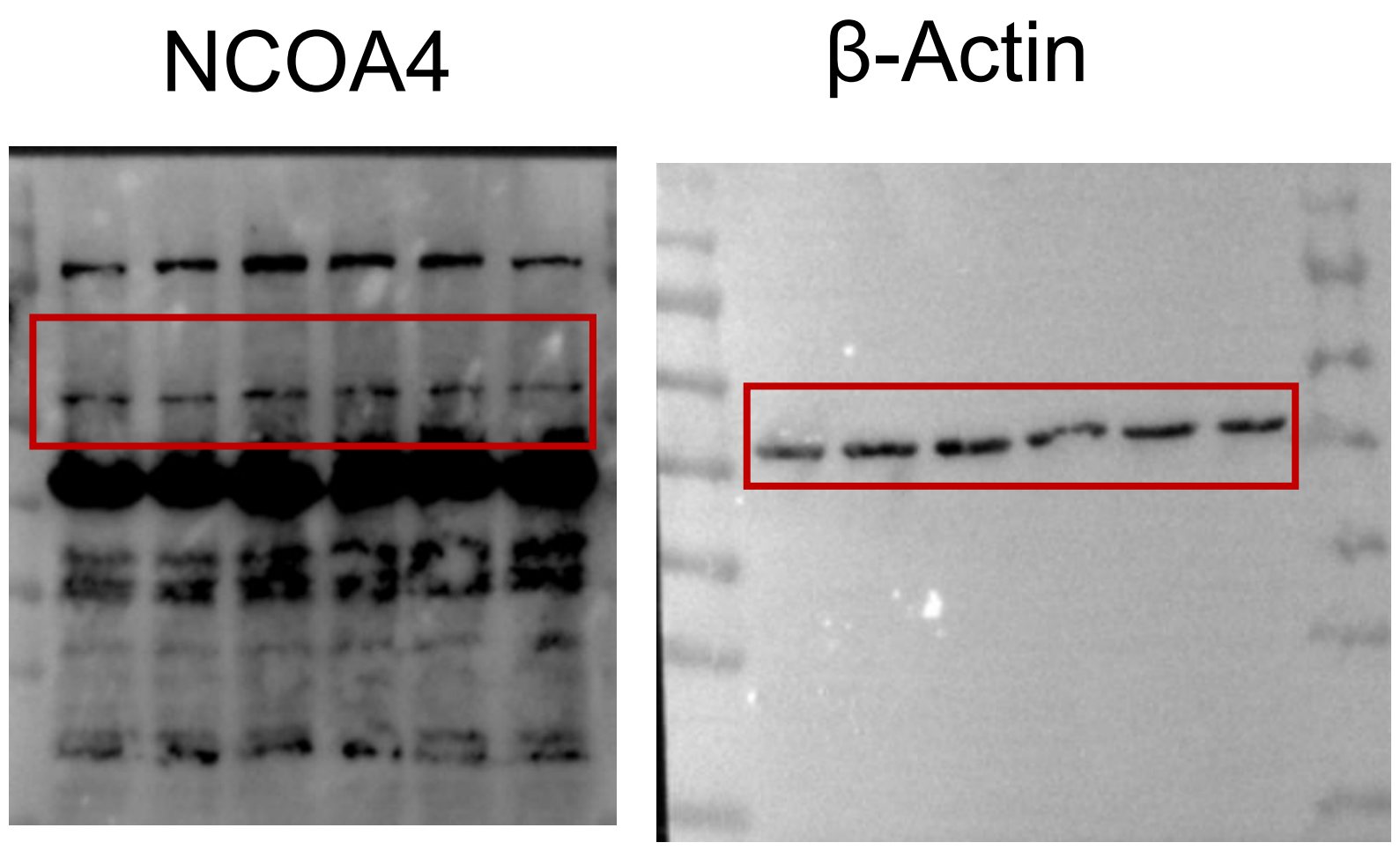

Figure S10

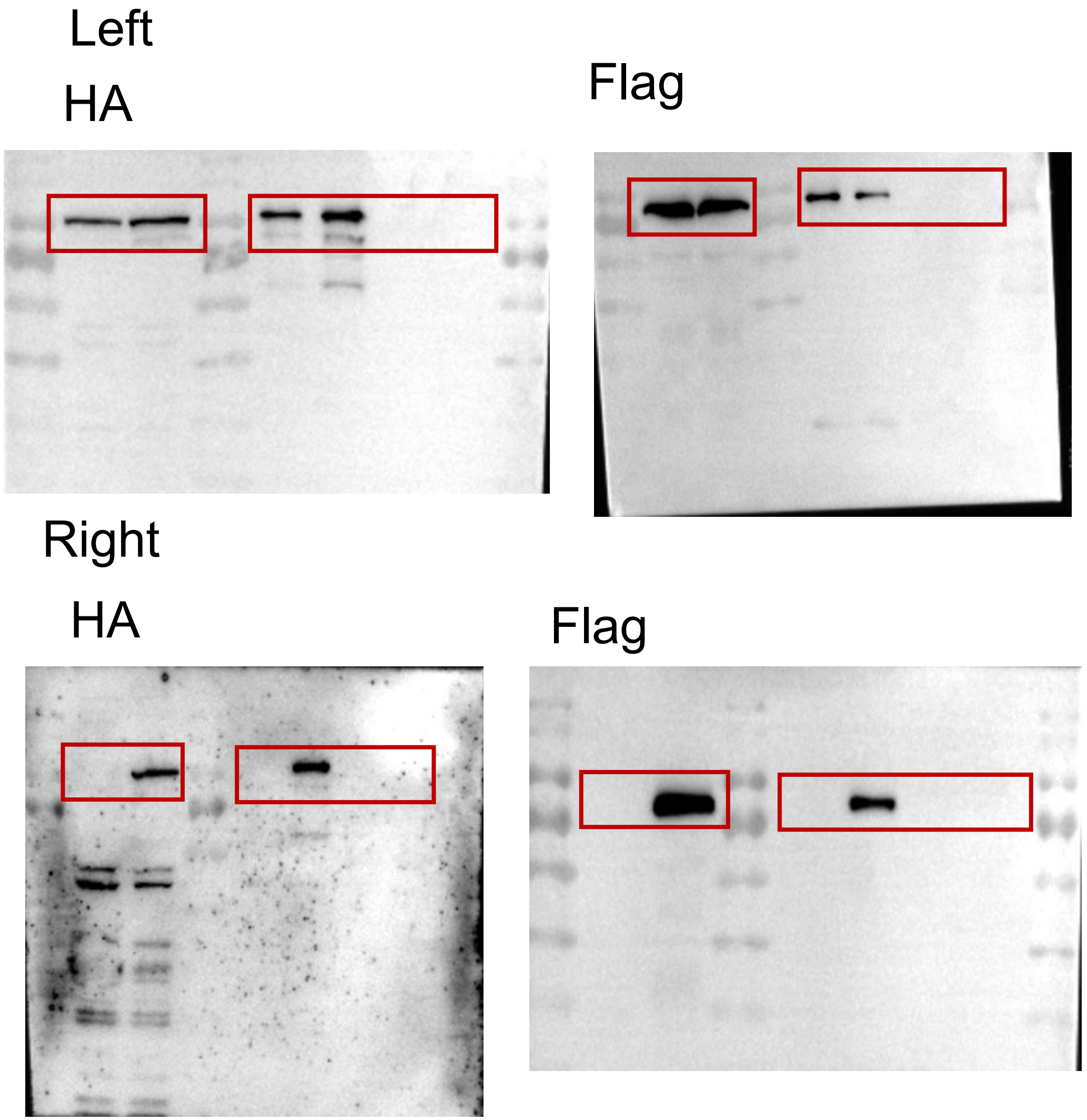

Figure S11

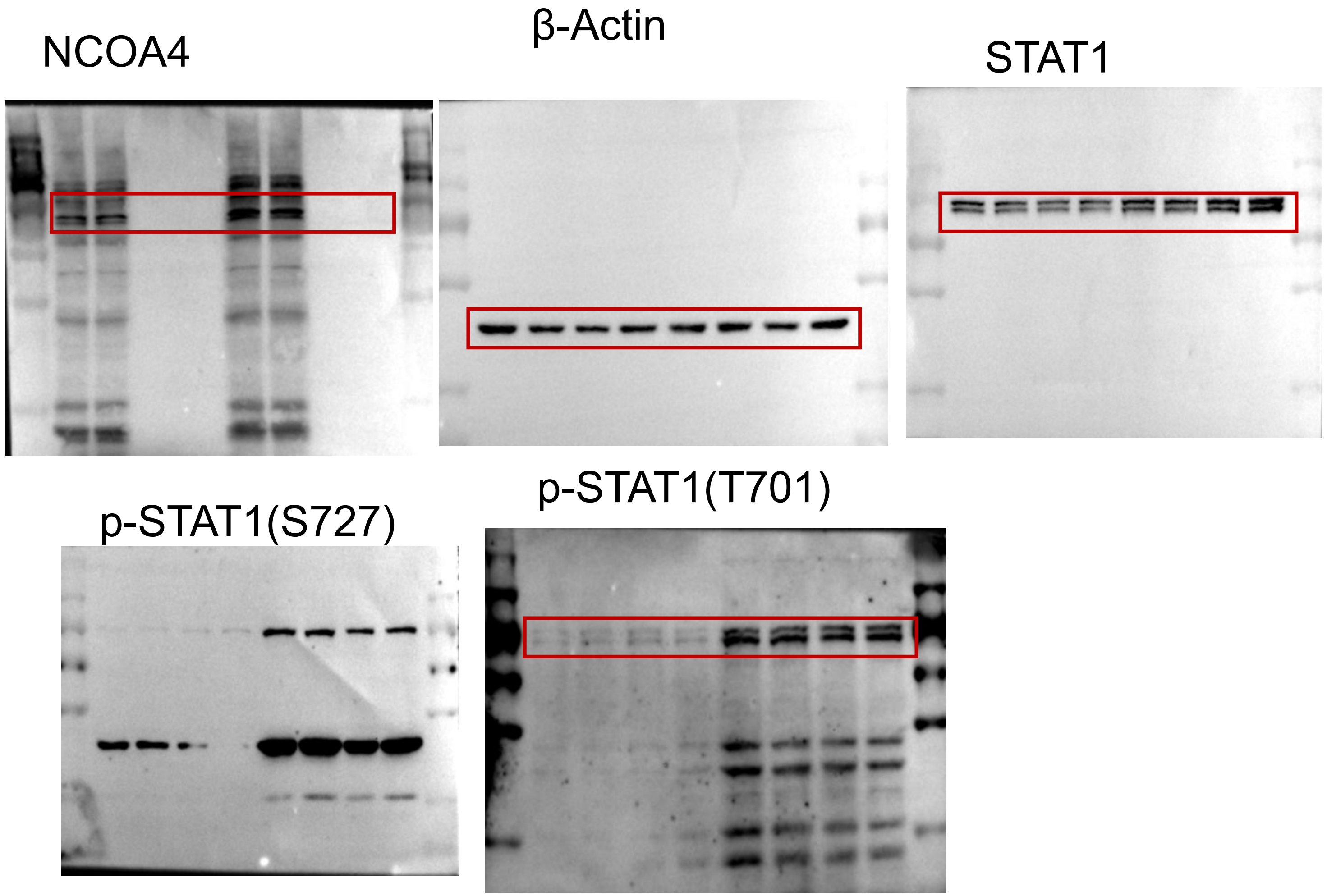

Figure S12

β-Actin

NCOA4

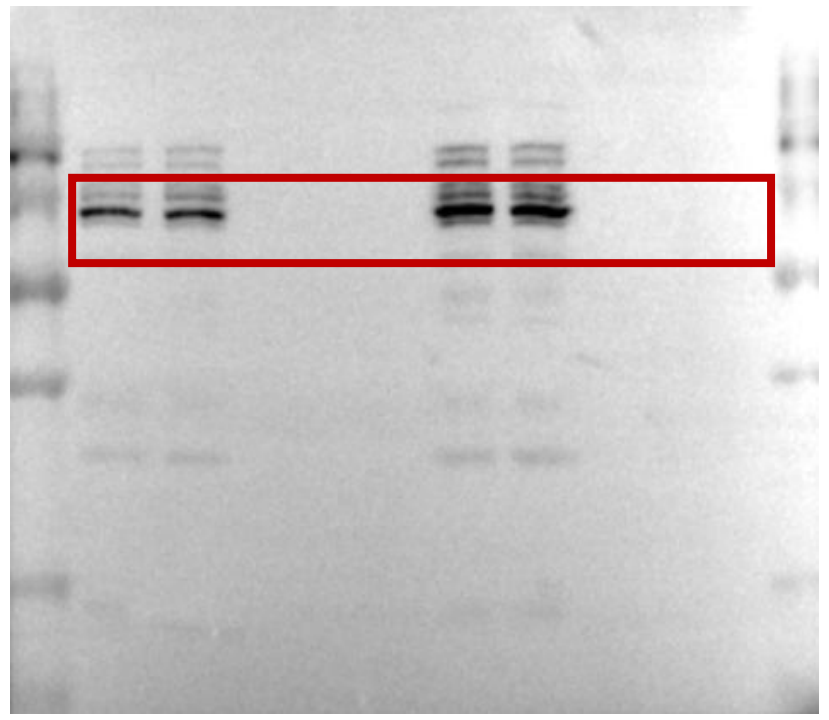

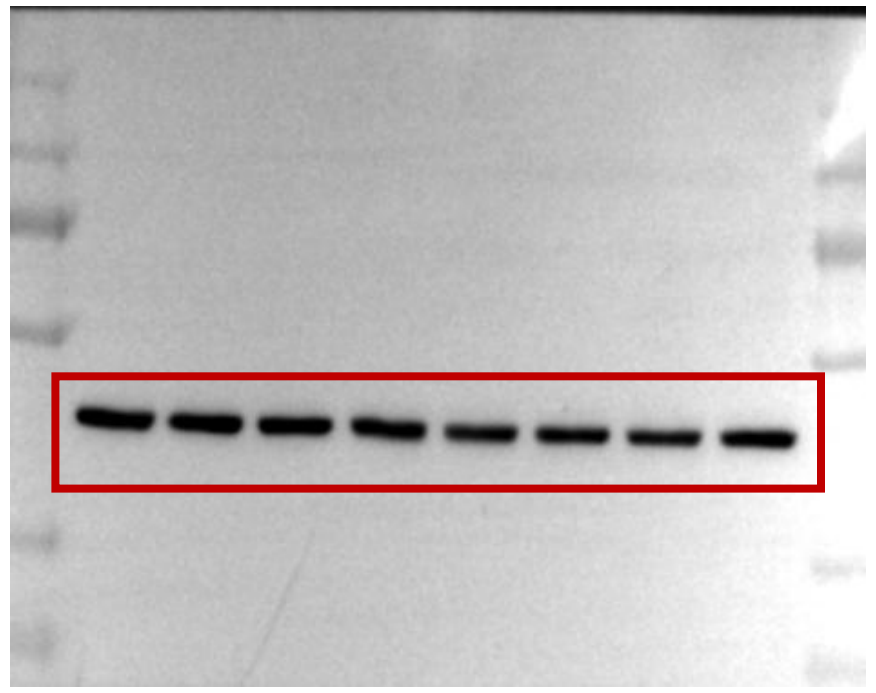

CH25H

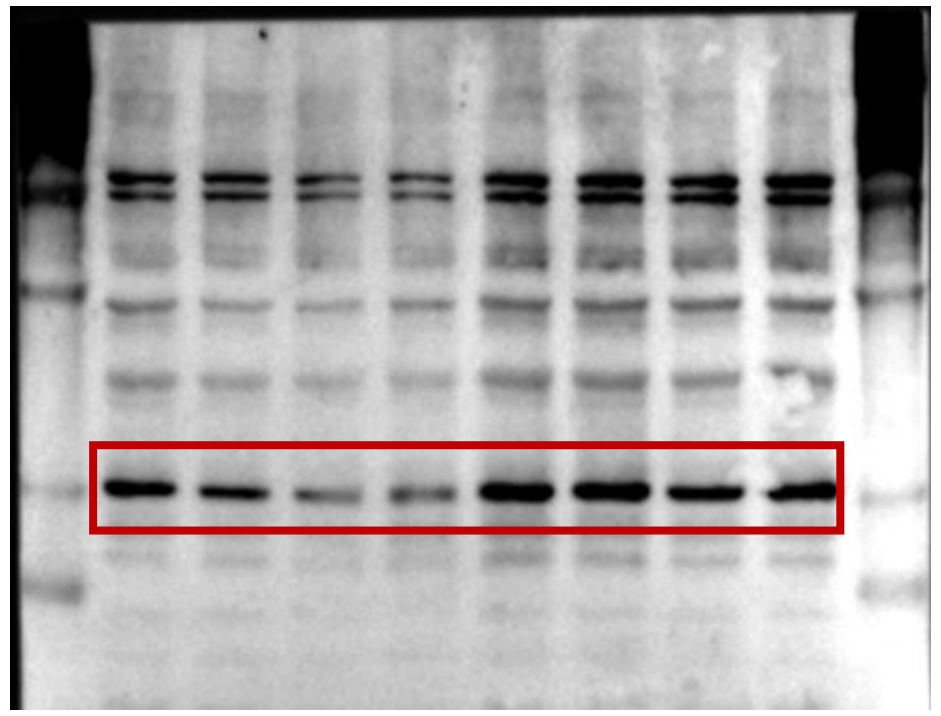

Figure S14

β-Actin

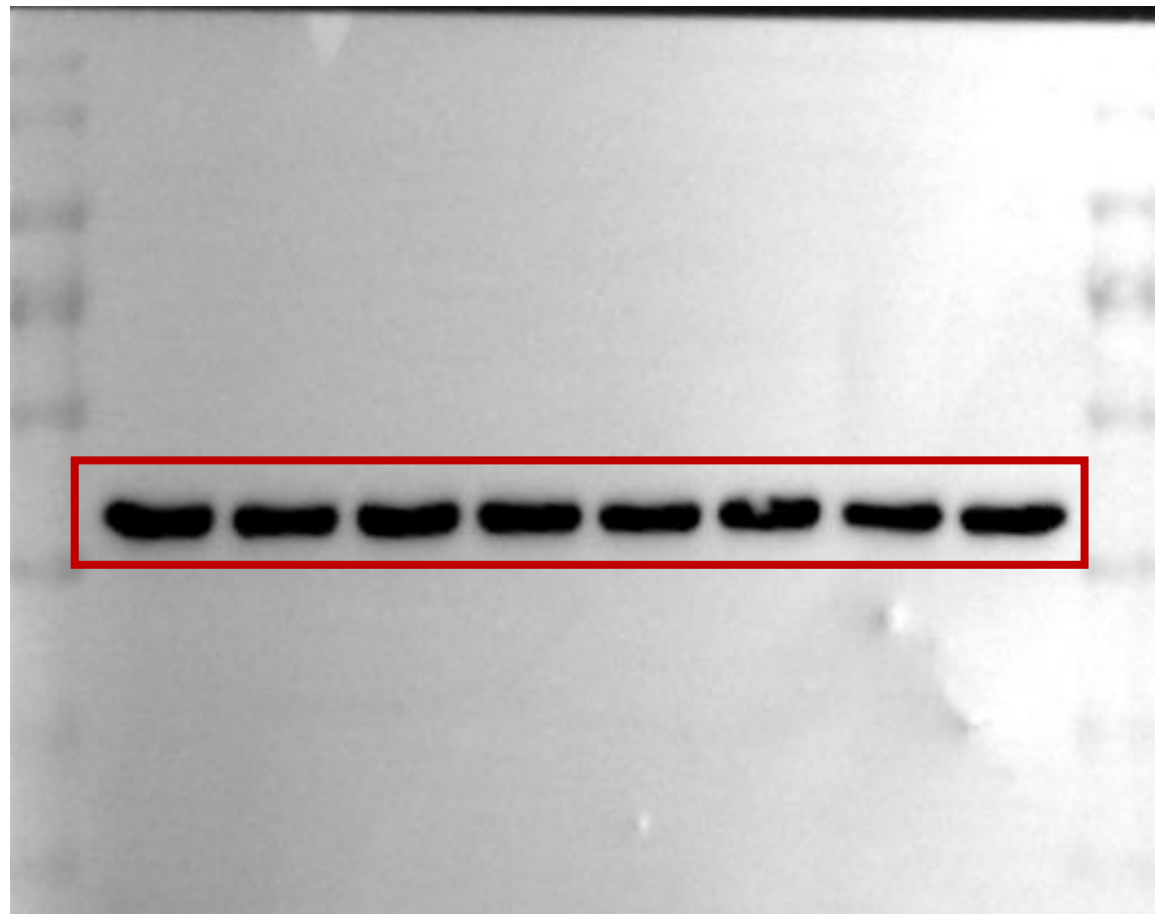

CH25H

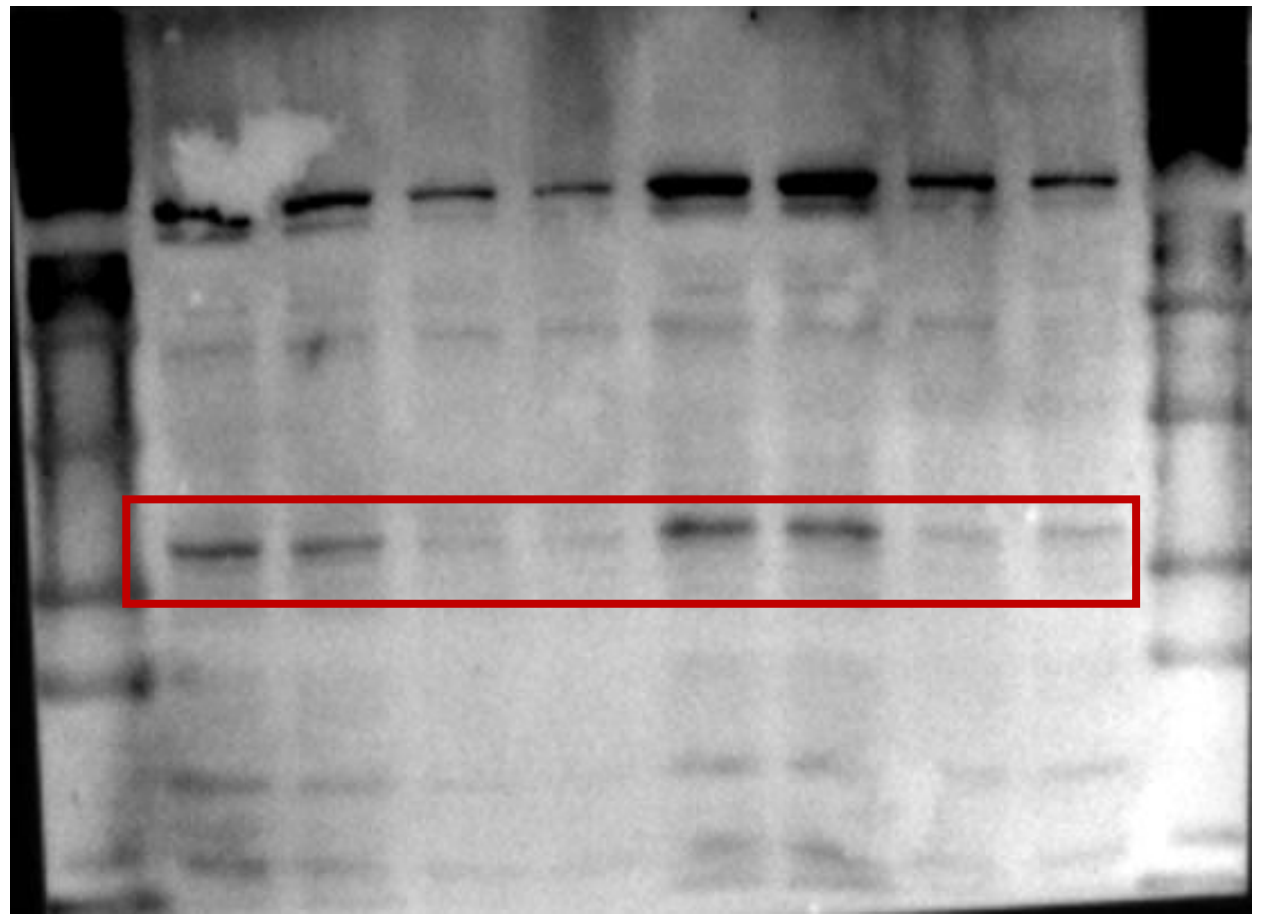

NRF2

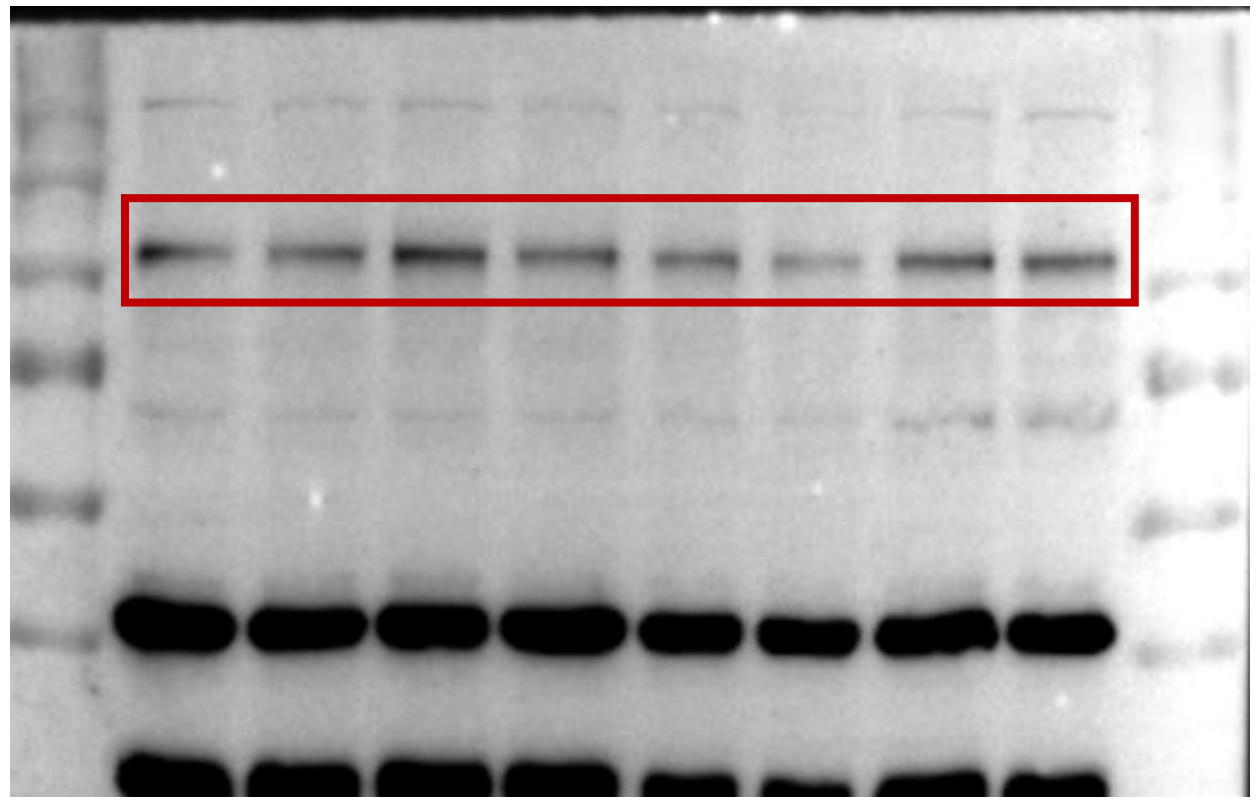

SLC7A11

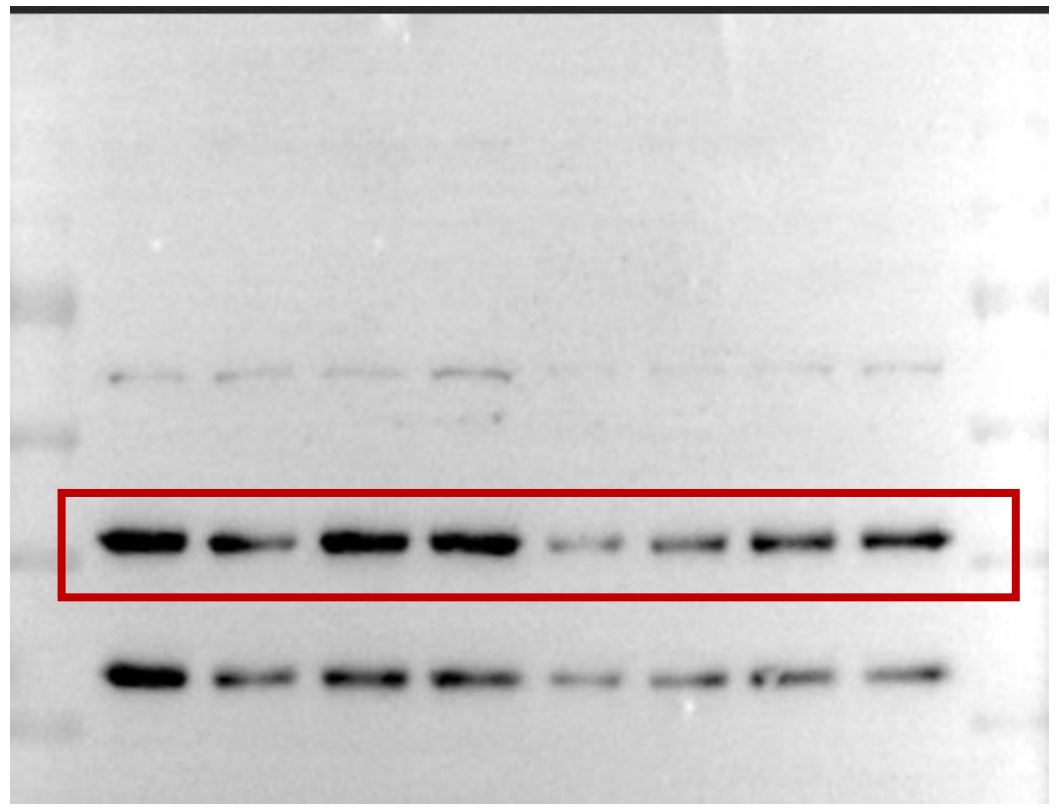

GPX4

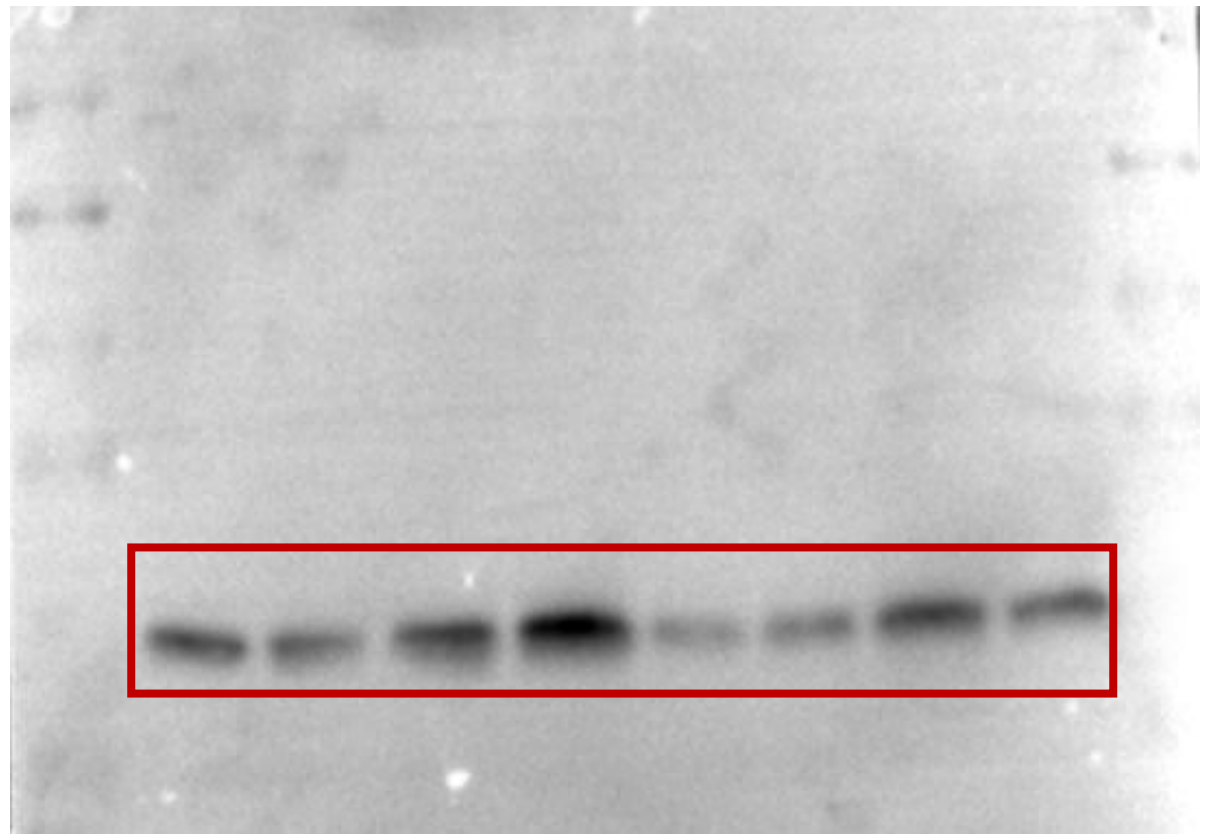

Figure S15A

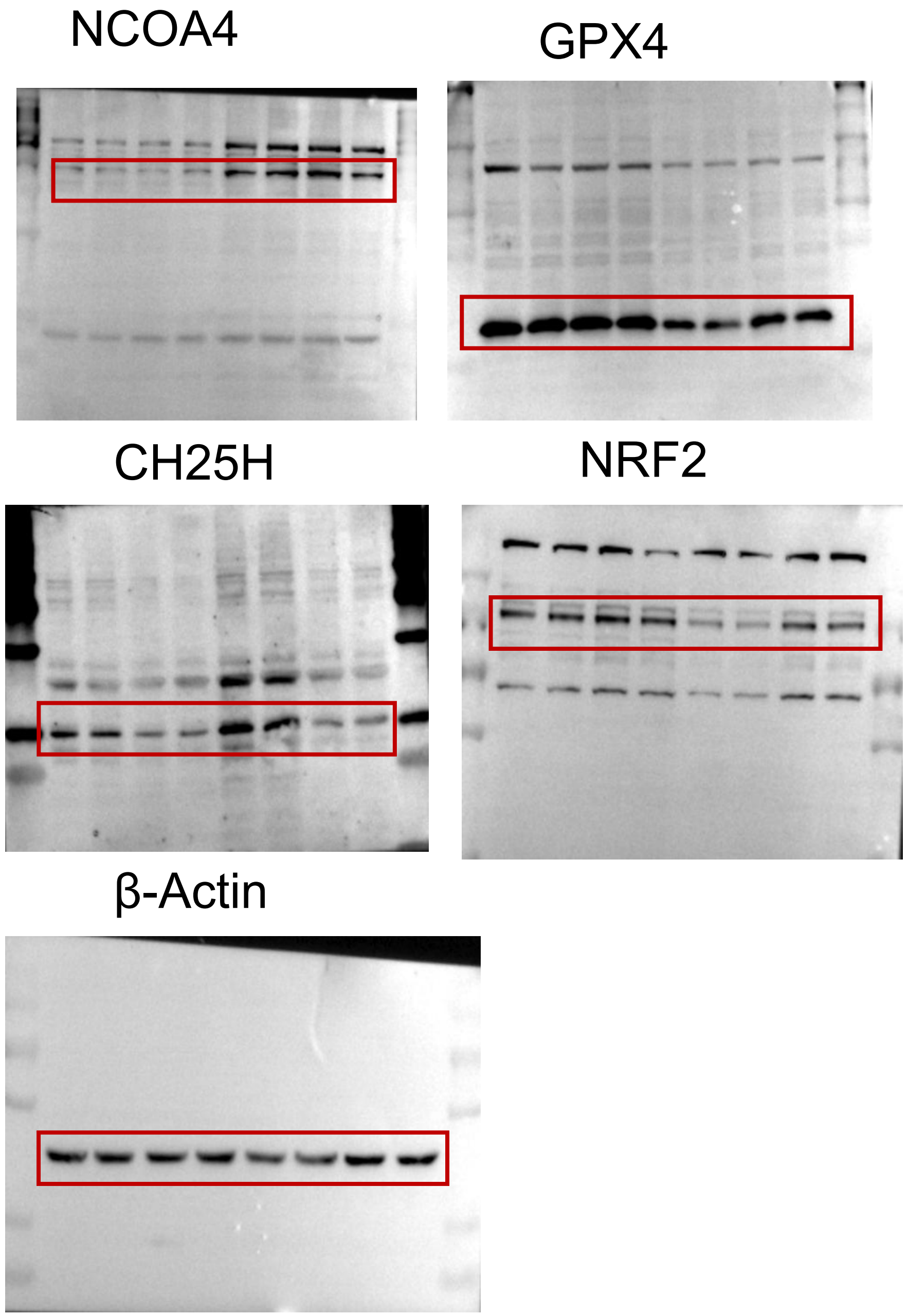

Figure S15B

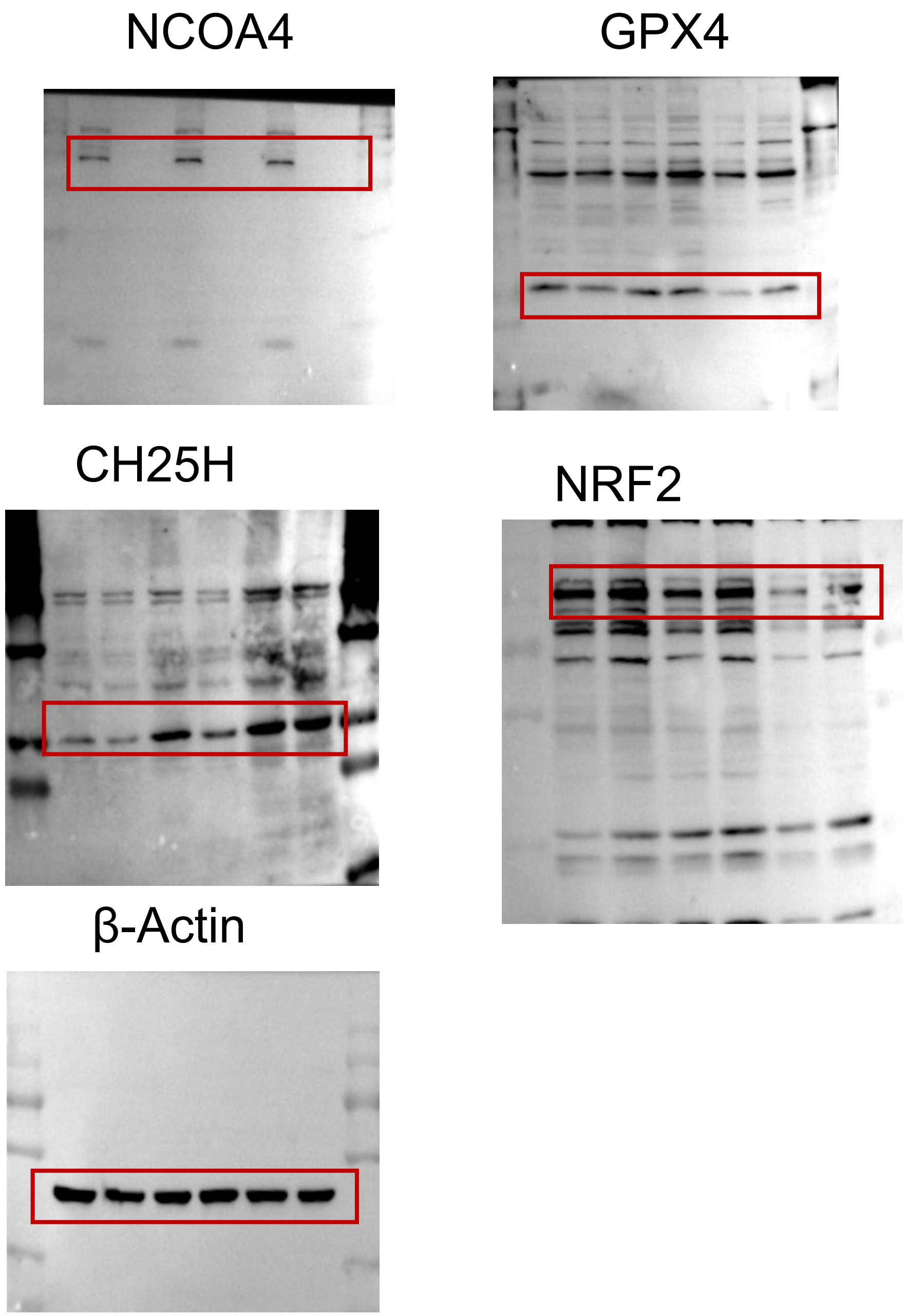

Supplement: Supplementary file 2 [file ADVS-9999-e78079-s003.pdf]
