# Supplementary material for: NCOA4 Accelerates Abdominal Aortic Aneurysm Formation by Regulating Macrophage Activation and Ferroptosis via STAT1–CH25H Axis
Source: Adv Sci (Weinh). 2026 Sep 29:e78079. Online ahead of print. doi: 10.1002/advs.78079 (PMC13624311; doi:10.1002/advs.78079)
Supplement: Supplementary file 3 [file ADVS-9999-e78079-s002.docx]

Supporting Information

NCOA4 Accelerates Abdominal Aortic Aneurysm Formation by Regulating Macrophage Activation and Ferroptosis via STAT1–CH25H Axis

Zhinan Wu, Yuxuan Qian, Wenyi Xu, Fei Liao, Xueliang Liu, Xiao Kuang, Guqing Luo, Xinjie He, Lixian Jiang*, Song Ding*

Zhinan Wu, Yuxuan Qian and Wenyi Xu contributed equally to this work.

**Supporting Figures**

**Figure S1**

**Figure S1.** TRIM3 mediated protein degradation of NCOA4. **A**, Scheme of IP/MS. **B**, KEGG enrichment of NCOA4 IP/MS result. **C**, Immunoprecipitation results of PMs. **D**, Immunoprecipitation results of HEK-293T transfected with TRIM3-HA plasmid and NCOA4-Flag plasmid. **E**, Immunoprecipitation results of HEK-293T transfected with TRIM3-HA plasmid, Ub-Myc plasmid and NCOA4-Flag plasmid. **F**, Western blot of HEK-293T transfected with TRIM3-HA plasmid and NCOA4-Flag plasmid and the quantification (n = 6 per group). Statistical analyses of **F** were analyzed by unpaired *t* test. *** indicates p < 0.001. IgG, immunoglobulin G; IP, immunoprecipitation; IP/MS, immunoprecipitation coupled with Mass Spectrometry; KEGG, Kyoto Encyclopedia Genes and Genomes; NCOA4, nuclear receptor coactivator 4; PM, peritoneal macrophage; TRIM3, tripartite motif containing 3; Ub, ubiquitin.

**Figure S2**

**Figure S2.** TRIM3 is Downregulated in Macrophages during AAA progression. **A**, Relative protein levels of TRIM3 in human aortic tissues (n = 6 per group). **B**, Relative mRNA levels of *TRIM3* in human aortic tissues (n = 6 per group). **C**, Relative protein levels of TRIM3 in mouse aortic tissues (n = 6 per group). **D**, Relative mRNA levels of *Trim3* in mouse aortic tissues (n = 6 per group). **E**, Western blot of PMs and the quantification (n = 6 per group). **F**, Relative mRNA levels of *Trim3* in PMs (n = 6 per group). **G**, Representative images of IF staining of PMs and the quantification (n = 6 per group). Scale bar indicates 50 μm. **H**, Relative protein levels and the quantification (n = 6 per group). I, Relative mRNA levels of *Trim3* in indicated groups (n = 6 per group). Data are expressed as mean ± SEM. Statistical analyses of **A** to **G** were analyzed by unpaired *t* test. Statistical analyses of **H** and **I** were analyzed by one-way analysis of variance (ANOVA) following Tukey’s multiple comparisons. * indicates p < 0.05; **, p < 0.01, ***, p < 0.001. Ang II, angiotensin II; CD68, cluster of differentiation 68; DAPI, 4',6-Diamidino-2'-phenylindole; MFI, mean Fluorescence Intensity; NAC, N-acetyl-l-cysteine; PM, peritoneal macrophage; TRIM3, tripartite motif containing 3.

**Figure S3**

**Figure S3.** Validation of *Ncoa4* Knockout. **A**, Validation of the effective inactivation of NCOA4 in the global *Ncoa4* knockout strain. PCR analysis was performed for the confirmation of successful gene editing. The 426 bp band represents wild type while the 599 bp represents the mutation. Primers for genotyping were listed in Table S3. **B**, Relative protein levels of NCOA4 in PMs from *Ncoa4*^+/+^ mice and *Ncoa4*^-/-^ mice (n = 6 per group). **C**, Relative mRNA levels of *Ncoa4* in PMs from *Ncoa4*^+/+^ mice and *Ncoa4*^-/-^ mice (n = 6 per group). Statistical analyses of **B** and **C** were analyzed by unpaired *t* test. Data are expressed as mean ± SEM. ** indicates p < 0.01; ****, p < 0.0001. HE, heterozygous; HO, homozygous; NCOA4, nuclear receptor coactivator 4; PCR, polymerase chain reaction; WT, wild type.

**Figure S4**

**Figure S4.** Expression of Ferroptosis related Protein. Relative protein levels in PMs from *Ncoa4*^+/+^ mice and *Ncoa4*^-/-^ mice (n = 6 per group). Statistical analyses were analyzed by unpaired *t* test. Data are expressed as mean ± SEM. *** indicates p < 0.001; ns, no significance. FTH1, ferritin heavy chain 1; GPX4, glutathione peroxidase 4.

**Figure S5**

**Figure S5.** Metabolic Profiles in *Ncoa4*^+/+^ Mice and *Ncoa4*^-/-^ Mice. **A**, Blood pressure of *Ncoa4*^+/+^ mice and *Ncoa4*^-/-^ mice infused with normal saline or Ang II (n = 8 per group). **B**, Lipid profile of *Ncoa4*^+/+^ mice and *Ncoa4*^-/-^ mice infused with normal saline or Ang II (n = 8 per group). Statistical analyses of **A** and **B** were analyzed by two-way ANOVA following Tukey’s multiple comparisons. Data are expressed as mean ± SEM. ns indicates no significance; ****, p < 0.0001. Ang II, angiotensin II; HDL-C, high-density lipoprotein cholesterol; LDL-C, low-density lipoprotein cholesterol; SBP, systolic blood pressure; TC, total cholesterol; TG, triglyceride.

**Figure S6**

**Figure S6.** *Ncoa4* Knockout Ameliorated Inflammation and ECM Degradation. **A**, Relative mRNA levels of *Il1b, Il6, Tnf* and *Ptgs2* in aortic tissues of *Ncoa4*^+/+^ mice and *Ncoa4*^-/-^ mice (n = 6 per group). **B**, Representative images of IF staining of infiltrated macrophages within aortic wall (marked with CD68, white arrow indicated infiltrated macrophages) and the quantification (n = 6 per group). Scale bar indicates 100 μm. **C**, Relative protein levels of MMP2 and MMP9 in aortic tissues of *Ncoa4*^+/+^ mice and *Ncoa4*^-/-^ mice (n = 6 per group). Data are expressed as mean ± SEM. Statistical analyses of **A**-**C** were performed by unpaired *t* test. ** indicates p < 0.01. MMP2, matrix metalloproteinase 2; MMP9, matrix metalloproteinase 9.

**Figure S7**

**Figure S7.** Validation of Bone Marrow Transplantation. **A**, PCR analysis was performed for validation of the effective bone marrow transplantation. The 426 bp band represents wild type while the 599 bp represents the mutation. **B**, Relative protein levels of NCOA4 in PMs from *Ncoa4*^+/+^→*Ncoa4*^+/+^ mice and *Ncoa4*^-/-^→*Ncoa4*^+/+^ mice (n = 6 per group). C, Representative images of IF staining of infiltrated macrophages within aortic wall (marked with CD68, white arrow indicated infiltrated macrophages) and the quantification (n = 6 per group). Scale bar indicates 100 μm. Statistical analyses were analyzed by unpaired *t* test. Data are expressed as mean ± SEM. ** indicates p < 0.01. Mut, mutant; NCOA4, nuclear receptor coactivator 4; PCR, polymerase chain reaction; PM, peritoneal macrophage; WT, wild type.

**Figure S8**

**Figure S8.** Deficiency of NCOA4 in Stromal-Derived Cells Is Insufficient to Promote AAA. **A**, Diagram of experiments procedure: Bone marrow cells from *Ncoa4*^+/+^ mice were transplanted to recipient *Ncoa4*^-/-^ mice and *Ncoa4*^+/+^ mice. After 2 weeks’ recovery, *Ncoa4*^-/-^ mice and *Ncoa4*^+/+^mice were injected with rAAV8/D377Y-mPCSK9 and then subjected to mini-osmotic pump implantation infused with Ang II (1000 ng/kg/min) for 28 days. All mice were fed with Paigen diet during the procedure. **B**, PCR analysis was performed for validation of the effective bone marrow transplantation. The 426 bp band represents wild type while the 599 bp represents the mutation. **C**, Relative protein levels of NCOA4 in PMs from *Ncoa4*^+/+^→*Ncoa4*^+/+^ mice and *Ncoa4*^+/+^→*Ncoa4*^-/-^ mice (n = 6 per group). **D**, Blood pressure of *Ncoa4*^+/+^→*Ncoa4*^+/+^ mice and *Ncoa4*^+/+^→*Ncoa4*^-/-^ mice infused Ang II (n = 8 per group). **E**, Lipid profile of *Ncoa4*^+/+^→*Ncoa4*^+/+^ mice and *Ncoa4*^+/+^→*Ncoa4*^-/-^ mice infused Ang II (n = 8 per group). Statistical analyses of **C** to **E** were analyzed by unpaired *t* test. Data are expressed as mean ± SEM. **F**, Representative images of macroscopic features of abdominal aortas in indicated groups, Scale bar indicates 4 mm. **G**, Survival curves in indicated groups (n = 20 for each group). Survival rates were analyzed by the Kaplan–Meier method and compared using log-rank tests. (In *Ncoa4*^+/+^→*Ncoa4*^+/+^ group, 4 mice died with isolated abdominal aortic rupture; In *Ncoa4*^+/+^→*Ncoa4*^-/-^ group, 2 mouse died with isolated abdominal aortic rupture, 1 mouse died with combined thoracic/abdominal aortic rupture prior to the termination) **H**, Incidence of AAA induced by Ang II in indicated groups (n = 16-17 per group). Data were analyzed by a Fisher exact test. **I**, Quantification of the maximal diameter of suprarenal abdominal aortas (n = 16-17 per group). Data was expressed as the mean ± SEM and analyzed by unpaired *t* test. **J**, Representative images of HE, Masson and EVG staining of crossed-sections of abdominal aortas. **K**, Grade of elastin degradation (n = 10 per group). Data was expressed as median with interquartile range and analyzed by Nonparametric Kruskal-Wallis test with Dunn’s post-hoc test. **L**, Quantitative analysis of collagen deposition (n = 10 per group). Data was expressed as the mean ± SEM and analyzed by unpaired *t* test. ns indicates no significance. AAA, abdominal aortic aneurysm; Ang II, angiotensin II; BMT, bone marrow transplant; EVG, elastic Van Gieson staining; HDL-C, high-density lipoprotein cholesterol; HE, hematoxylin-eosin staining; LDL-C, low-density lipoprotein cholesterol; SBP, systolic blood pressure; TC, total cholesterol; TG, triglyceride.

**Figure S9**

**Figure S9. Macrophage Specific NCOA4 Deficiency Attenuated AAA Progression.** **A**, Diagram of experiments procedure:6-week-old *ApoE*^-/-^ mice were randomly subjected to 2 groups and injected with AAV-Sh*Ncoa4* or AAV-Sh*Ctrl.* 2 weeks later, the mice subjected to mini-osmotic pump implantation. All mice were fed with Western diet during the procedure. **B**, Representative macroscopic images of abdominal aortas in indicated groups. Scale bars indicate 4 mm. **C**, Incidence of AAA induced by Ang II in indicated groups (n = 8-16 per group). Data were analyzed by a Fisher exact test. **D**, Quantification of the maximal diameter of suprarenal abdominal aortas (n = 8-16 per group). **E**, Representative images of HE, Masson and EVG staining of crossed-sections of abdominal aortas, Scale bar indicates 100 μm. **F**, Grade of elastin degradation (n = 8 per group). Data was analyzed by Nonparametric Kruskal-Wallis test with Dunn’s post-hoc test. **G**, Quantitative analysis of collagen deposition (n = 8 per group). Data from **D** and **G** were expressed as the mean ± SEM and analyzed by two-way ANOVA following Tukey’s multiple comparisons. ns indicates no significance, * indicates p < 0.05, ** indicates p < 0.01, *** indicates p < 0.001, **** indicates p < 0.0001. AAA, abdominal aortic aneurysm; Ang II, angiotensin II; EVG, elastic Van Gieson staining; HE, hematoxylin-eosin staining.

**Figure S10**

**Figure S10.** NCOA4/STAT1 Complex Synergistically Modulates Macrophage Activation. **A**, Heatmap of DEGs in PMs from *Ncoa4*^+/+^ mice and *Ncoa4*^-/-^ mice (n = 4 per group). **B**, Co-immunoprecipitation results of HEK-293T transfected with *Stat1*-HA plasmid and *Ncoa4*-Flag plasmid. DEGs, differentially expressed genes; IgG, immunoglobulin G; IP, immunoprecipitation; IP/MS, immunoprecipitation coupled with Mass Spectrometry; KEGG, Kyoto Encyclopedia Genes and Genomes; NCOA4, nuclear receptor coactivator 4; PM, peritoneal macrophage; STAT1, signal transducer and activator of transcription 1.

**Figure S11**

**Figure S11.** NCOA4 deficiency suppressed STAT1 transcriptional activity. Expression levels of NCOA4, STAT1, p-STAT1-T701 and p-STAT1-S727 in PMs (n = 6 per group). Data was expressed as the mean ± SEM and analyzed by two-way ANOVA following Tukey’s multiple comparisons. ns indicates no significance; *, p < 0.05; **, p < 0.01; ***, p < 0.001; ****; p < 0.0001. Ang II, angiotensin II; NCOA4, nuclear receptor coactivator 4; PBS, Phosphate-Buffered Saline; PM, peritoneal macrophage; STAT1, signal transducer and activator of transcription 1; p-STAT1-T701, STAT1 phosphorylation at tyrosine 701; p-STAT1-S727, STAT1 phosphorylation at serine 727.

**Figure S12**

**Figure S12.** NCOA4 deficiency BMDM showed less CH25H expression. Expression levels of NCOA4 and CH25H in BMDMs (n = 6 per group). Data was expressed as the mean ± SEM and analyzed by two-way ANOVA following Tukey’s multiple comparisons. ****; p < 0.0001. Ang II, angiotensin II; Bone Marrow-Derived Macrophages, BMDMs; CH25H, cholesterol 25-hydroxylase; NCOA4, nuclear receptor coactivator 4; PBS, Phosphate-Buffered Saline.

**Figure S13**

**Figure S13.** *Ch25h* Knockdown Prevented Ang II induced Macrophage Inflammation. **A**, 25-HC levels in culture medium of PMs in indicated groups (n = 6 per group). **B**, Relative mRNA levels of *Il1b, Il6, Tnf* and *Ptgs2* in PMs in indicated groups (n = 6 per group). Data of **A** and **B** was expressed as the mean ± SEM and analyzed by two-way ANOVA following Tukey’s multiple comparisons. ns indicates no significance; *, p < 0.05, ***, p < 0.001; ****, p < 0.0001. 25-HC, 25-Hydroxycholesterol; Ang II, angiotensin II; PBS, Phosphate-Buffered Saline; PM, peritoneal macrophage.

**Figure S14**

**Figure S14.** *Ch25h* Knockdown Prevented Ang II induced Macrophage Ferroptosis. **A**, Ferroptosis related protein levels of PMs in indicated groups (n = 6 per group). **B**, Biochemical tests of PMs in indicated groups (n = 6 per group). **C**, Mitotracker Red, FerroOrrange and MitoPeDPP staining of PMs in indicated groups (n = 6 per group). Data of **A** to **C** was expressed as the mean ± SEM and analyzed by two-way ANOVA following Tukey’s multiple comparisons. ns indicates no significance; *, p < 0.05; **, p < 0.01; ***, p < 0.001; ****; p < 0.0001. FTH1, ferritin heavy chain 1; GPX4, glutathione peroxidase 4; GSH, reduced glutathione; GSSG, oxidized glutathione disulfide; MDA, Malonic dialdehyde; MFI, mean Fluorescence Intensity; NCOA4, nuclear receptor coactivator 4; NRF2, Nuclear factor erythroid 2-related factor 2; SLC7A11, solute carrier family 7 member 11.

**Figure S15**

**Figure S15.** NCOA4/STAT1/CH25H contributed to Ang II induced Macrophage Ferroptosis. **A**, Ferroptosis related protein levels of PMs in indicated groups (n = 6 per group). **B**, Ferroptosis related protein levels of PMs in indicated groups (n = 6 per group). Data was expressed as the mean ± SEM and analyzed by two-way ANOVA following Tukey’s multiple comparisons. ns indicates no significance; *, p < 0.05; **, p < 0.01; ***, p < 0.001; ****; p < 0.0001. Ang II, angiotensin II; CH25H, cholesterol 25-hydroxylase; GPX4, glutathione peroxidase 4; PBS, Phosphate-Buffered Saline; NCOA4, nuclear receptor coactivator 4; NRF2, Nuclear factor erythroid 2-related factor 2; S STAT1, signal transducer and activator of transcription 1.

**Figure S16.**

**Figure S16.** Additional STAT1 ablation did not augment the protective effects against AAA conferred by NCOA4 deficiency. **A**, Diagram of experiments procedure:6-week-old *ApoE*^-/-^ mice were randomly subjected to 4 groups and injected with AAV-Sh*Ncoa4* or AAV-Sh*Ctrl* and AAV-Sh*Stat1* or AAV-Sh*Nc.* 2 weeks later, the mice subjected to mini-osmotic pump implantation. All mice were fed with Western diet during the procedure. **B**, Representative macroscopic images of abdominal aortas in indicated groups. Scale bars indicate 4 mm. **C**, Quantification of the maximal diameter of suprarenal abdominal aortas (n = 8-12 per group). **D**, Representative images of HE, Masson and EVG staining of crossed-sections of abdominal aortas, Scale bar indicates 100 μm. **E**, Grade of elastin degradation (n = 8 per group). Data was analyzed by Nonparametric Kruskal-Wallis test with Dunn’s post-hoc test. **F**, Quantitative analysis of collagen deposition (n = 8 per group). Data from **C** and **F** were expressed as the mean ± SEM and analyzed by two-way ANOVA following Tukey’s multiple comparisons. ns indicates no significance, * indicates p < 0.05, ** indicates p < 0.01, *** indicates p < 0.001, **** indicates p < 0.0001. AAA, abdominal aortic aneurysm; Ang II, angiotensin II; EVG, elastic Van Gieson staining; HE, hematoxylin-eosin staining.

**Figure S17.**

**Figure S17.** Schematic illustration of fabrication of the SI-*Ch25h*-LNP/MBs *via* microfluidic mixing method. Created in BioRender. P, X. (2026) https://BioRender.com/99d01vg  **Figure S18**

**Figure S18.** Knockdown *Ch25h* in Macrophage within Aneurysm Prevents AAA Progression. **A**, Bright-field microscopy images of the MB and SI-*Ch25h*-LNP/MB. Scale bars: 50 μm. **B**, Hydrodynamic diameter distribution of the MB and SI-*Ch25h*-LNP/MB in aqueous solution measured by Particle Sizing Systems (PSS). **C**, Surface zeta potential measurements of the SI-*Ch25h*-LNP, MB and SI-*Ch25h*-LNP/MB obtained *via* nanoparticle tracking analysis. **D**, Diameter distribution of the SI-*Ch25h*-LNP, MB and SI-*Ch25h*-LNP/MB measured by dynamic light scattering (DLS). **E**, *In vitro* hemodynamic model used to test the ability of Cy5-LNP/MB to be retained with or without ultrasound destruction. Left: almost no retention of flowing Cy5-LNP/MB in the microfluidic hydrogel tube wall without ultrasound destruction. Right: flowing Cy5-LNP/MB were clearly retained in the microfluidic hydrogel tube wall with ultrasound destruction. **F,** Representative contrast-enhanced ultrasound images of a mouse pre-injection, post-injection, microbubble destruction mode, and post-destruction of the SI-*Ch25h*-LNP/MB. **G,** Representative immunofluorescence images showing CH25H expression in mouse aortic tissues, with or without SI-*Ch25h* intervention, counterstained with DAPI (n = 6 per group). White line indicates the vascular tone. Scale bar indicates 100 μm. Data are expressed as mean ± SEM and analyzed by Brown-Forsythe and Welch ANOVA tests. ns indicates no significance; ***, p < 0.001; ****, p < 0.0001. CH25H, cholesterol 25-hydroxylase; DAPI, 4',6-Diamidino-2'-phenylindole; LNP, lipid nanoparticle; MB, microbubble.
**Figure S19**

**Figure S19.** Metabolic Profiles of *ApoE*^-/-^ Mice from indicated groups. **A**, Blood pressure of *ApoE* ^-/-^ mice from indicated groups (n = 8 per group). **B**, Lipid profile of *ApoE* ^-/-^ mice from indicated groups (n = 8 per group). C, Representative images of IF staining of infiltrated macrophages within aortic wall (marked with CD68, white arrow indicated infiltrated macrophages) and the quantification (n = 6 per group). Scale bar indicates 100 μm. Statistical analyses of **A** and **B** were analyzed by two-way ANOVA following Tukey’s multiple comparisons. Statistical analysis of **C** was analyzed by unpaired *t* test. Data are expressed as mean ± SEM. ns indicates no significance; ** indicates p < 0.01; ****, p < 0.0001. Ang II, angiotensin II; HDL-C, high-density lipoprotein cholesterol; LDL-C, low-density lipoprotein cholesterol; LNP/MB, lipid nanoparticle/microbubble; SBP, systolic blood pressure; TC, total cholesterol; TG, triglyceride.

**Figure S20**

**Figure S20.** *In vivo* evaluation of biodistribution of Cy5-LNP/MB and Cy5-LNP/MB with ultrasound destruction. **A,** Representative *ex vivo* fluorescence imaging maps of major organs from each group at 24 hours after invention. From top to bottom: heart, liver, spleen, lung, kidney (n = 3 per group). **B,** Representative *ex vivo* fluorescence imaging maps of aorta from each group at 24 hours after invention. (n = 3 per group). **C,** Representative immunofluorescence images and quantitative analyses of aorta showing the distribution of Cy5 loaded LNP/MB with or without ultrasound destruction (n = 3 per group). Blue: DAPI; Red: Cy5. Data are expressed as mean ± SEM and analyzed by Brown-Forsythe and Welch ANOVA tests. *, p < 0.05. DAPI, 4',6-Diamidino-2'-phenylindole; LNP, lipid nanoparticle; MB, microbubble.

**Figure S21**

**Figure S21.** Biosafety evaluation of SI-*Ch25h*-LNP/MB and SI-*Ch25h*-LNP/MB with ultrasound destruction, compared with control group. **A**, Representative images of HE staining of crossed-sections of multiple organs (including heart, liver, spleen, lung, and kidney) from mice of each group (n = 3 per group). Scale bar indicates 100 μm. **B**, Quantification of liver and kidney function indices from mice of each group (n = 3 per group). Data from **B** were expressed as the mean ± SEM and analyzed by two-way ANOVA following Tukey’s multiple comparisons. ns indicates no significance. HE, hematoxylin-eosin staining; LNP/MB, lipid nanoparticle loaded microbubble.

**Figure S22**

**Figure S22.** Targeting CH25H *via* LNP/MBs attenuates AAA progression. **A**, Diagram of experiments procedure*.* **B**, Representative macroscopic images of abdominal aortas in indicated groups. Scale bars indicate 4 mm. **C**, Quantification of the maximal diameter of suprarenal abdominal aortas (n = 10 per group). **D**, Representative images of HE, Masson and EVG staining of crossed-sections of abdominal aortas, Scale bar indicates 100 μm. **E**, Grade of elastin degradation (n = 8 per group). Data was analyzed by Nonparametric Kruskal-Wallis test with Dunn’s post-hoc test. **F**, Quantitative analysis of collagen deposition (n = 8 per group). Data from **C** and **F** were expressed as the mean ± SEM and analyzed by two-way ANOVA following Tukey’s multiple comparisons. ns indicates no significance, * indicates p < 0.05, ** indicates p < 0.01, *** indicates p < 0.001, **** indicates p < 0.0001. AAA, abdominal aortic aneurysm; Ang II, angiotensin II; EVG, elastic Van Gieson staining; HE, hematoxylin-eosin staining; LNP/MB, lipid nanoparticle loaded microbubble.

**Supplementary Tables**

**Table S1 Characteristics of the individuals enrolled in the human tissue study**

|  | AAA group  (N = 6) | Control group  (N = 6) | *P*-value |
| --- | --- | --- | --- |
| Age (yrs) | 71.83 ± 3.03 | 70.33 ± 5.31 | 0.811 |
| Male gender (n, %) | 3 (50) | 3 (50) | 1.000 |
| Smoking (n, %) | 0 (0) | 0 (0) | 1.000 |
| Body mass index (kg/m^2^) | 24.07 ± 2.53 | 22.73 ± 4.51 | 0.538 |
| Hypertension (n, %) | 6 (100) | 6 (100) | 1.000 |
| Diabetes Mellitus (n, %) | 3 (50) | 2 (33.3) | 0.599 |
| Total cholesterol (mmol/L) | 3.82 ± 1.35 | 4.24 ± 1.45 | 0.615 |
| HDL (mmol/L) | 0.96 ± 0.11 | 1.17 ± 0.33 | 0.173 |
| Triglyceride (mmol/L) | 1.50 ± 1.07 | 1.56 ± 1.31 | 0.929 |
| Fasting Blood Glucose (mmol/L) | 5.00 ± 0.33 | 5.12 ± 1.28 | 0.825 |
| HbA1c (%) | 5.95 ± 0.48 | 6.10 ± 0.93 | 0.730 |
| CRP (mg/L) | 34.28 ± 4.61 | 5.37 ± 0.89 | 0.000 |
| ACEI /ARB (n, %) | 1 (16.7) | 3 (50) | 0.260 |
| Beta-blockers (n, %) | 1 (16.7) | 2 (33.3) | 0.549 |
| CCB (n, %) | 5 (83.3) | 3 (50) | 0.260 |
| Anti-diabetic medications (n, %) | 3 (50) | 1 (16.7) | 0.260 |

Data are shown as mean ± SEM or frequencies (%). Comparisons of continuous variables were performed by Student’s t-test for normally-distributed variables or the Mann–Whitney U test for variables that were not normally distributed. Comparisons of categorical variables were conducted with the Chi-squared test or Fisher’s exact test. Abbreviations: HDL, high-density lipoprotein; CRP, C-reactive protein; ACEI, angiotensin-converting enzyme inhibitor; ARB, angiotensin receptor blocker; CCB, calcium channel blocker; HbA1c, Glycosylated Hemoglobin A1c.

**Table S2 Characteristics of the participants enrolled in the PBMCs study**

|  | AAA group  (N = 10) | Control group  (N = 10) | *P*-value |
| --- | --- | --- | --- |
| Age (yrs) | 71.60 ± 2.31 | 72.50 ± 3.52 | 0.833 |
| Male gender (n, %) | 7 (70) | 7 (70) | 1.000 |
| Smoking (n, %) | 0 (0) | 0 (0) | 1.000 |
| Body mass index (kg/m^2^) | 23.35 ± 0.76 | 24.44 ± 1.31 | 0.483 |
| Hypertension (n, %) | 8 (80) | 8 (80) | 1.000 |
| Diabetes Mellitus (n, %) | 7 (70) | 3 (30) | 0.081 |
| Total cholesterol (mmol/L) | 3.72 ± 0.36 | 3.82 ± 0.36 | 0.851 |
| HDL (mmol/L) | 0.98 ± 0.04 | 1.12 ± 0.09 | 0.164 |
| Triglyceride (mmol/L) | 1.27 ± 0.32 | 1.31 ± 0.29 | 0.911 |
| Fasting Blood Glucose (mmol/L) | 5.37 ± 0.31 | 5.14 ± 0.32 | 0.617 |
| HbA1c (%) | 6.02 ± 0.23 | 5.83 ± 0.12 | 0.480 |
| CRP (mg/L) | 28.65 ± 3.53 | 7.87 ± 1.19 | 0.000 |
| ACEI /ARB (n, %) | 4 (40) | 1 (10) | 0.135 |
| Beta-blockers (n, %) | 3 (30) | 2 (20) | 0.628 |
| CCB (n, %) | 6 (60) | 4 (40) | 0.398 |
| Anti-diabetic medications (n, %) | 7 (70) | 3 (30) | 0.081 |

Data are shown as mean ± SEM or frequencies (%). 20 study participants (10 AAA patients and 10 matched control subjects) were recruited in the metabolomics study. AAA is diagnosed in accordance with the Society for Vascular Surgery Practice Guidelines. Comparisons of continuous variables were performed by Student’s *t*-test for normally-distributed variables or the Mann–Whitney *U* test for variables that were not normally distributed. Comparisons of categorical variables were conducted with the Chi-squared test or Fisher’s exact test. Abbreviations: HDL, high-density lipoprotein; CRP, C-reactive protein; ACEI, angiotensin-converting enzyme inhibitor; ARB, angiotensin receptor blocker; CCB, calcium channel blocker; HbA1c, Glycosylated Hemoglobin A1c.

**Table S3 Primers sequences used in this study**

| Gene | Species | Primer | Sequence | Application |
| --- | --- | --- | --- | --- |
| *Actb* | Mouse | Forward | GGCTGTATTCCCCTCCATCG | RT-qPCR |
|  |  | Reverse | CCAGTTGGTAACAATGCCATGT | RT-qPCR |
| *Ncoa4* | Mouse | Forward | GCCCTACAATGTGAGTGATTGG | RT-qPCR |
|  |  | Reverse | ACTGGTGCAAGGCTCGTTG | RT-qPCR |
| *Trim3* | Mouse | Forward | GCGTCTCAGCCCTACAAAACA | RT-qPCR |
|  |  | Reverse | AAACTCCATTGTCTTGCCTTCA | RT-qPCR |
| *Arg1* | Mouse | Forward | CTCCAAGCCAAAGTCCTTAGAG | RT-qPCR |
|  |  | Reverse | AGGAGCTGTCATTAGGGACATC | RT-qPCR |
| *Thbs2* | Mouse | Forward | CTGGGCATAGGGCCAAGAG | RT-qPCR |
|  |  | Reverse | GCTTGACAATCCTGTTGAGATCA | RT-qPCR |
| *Ccr5* | Mouse | Forward | TTTTCAAGGGTCAGTTCCGAC | RT-qPCR |
|  |  | Reverse | GGAAGACCATCATGTTACCCAC | RT-qPCR |
| *Irf7* | Mouse | Forward | GAGACTGGCTATTGGGGGAG | RT-qPCR |
|  |  | Reverse | GACCGAAATGCTTCCAGGG | RT-qPCR |
| *Ccl5* | Mouse | Forward | GCTGCTTTGCCTACCTCTCC | RT-qPCR |
|  |  | Reverse | TCGAGTGACAAACACGACTGC | RT-qPCR |
| *Gdf3* | Mouse | Forward | ATGCAGCCTTATCAACGGCTT | RT-qPCR |
|  |  | Reverse | AGGCGCTTTCTCTAATCCCAG | RT-qPCR |
| *Ch25h* | Mouse | Forward | TGCTACAACGGTTCGGAGC | RT-qPCR |
|  |  | Reverse | AGAAGCCCACGTAAGTGATGAT | RT-qPCR |
| *Ciita* | Mouse | Forward | TGCGTGTGATGGATGTCCAG | RT-qPCR |
|  |  | Reverse | CCAAAGGGGATAGTGGGTGTC | RT-qPCR |
| *Nfatc4* | Mouse | Forward | GAGCTGGAATTTAAGCTGGTGT | RT-qPCR |
|  |  | Reverse | CATGGAGGGGTATCCTCTGAG | RT-qPCR |
| *Il1b* | Mouse | Forward | GCAACTGTTCCTGAACTCAACT | RT-qPCR |
|  |  | Reverse | ATCTTTTGGGGTCCGTCAACT | RT-qPCR |
| *Il6* | Mouse | Forward | TAGTCCTTCCTACCCCAATTTCC | RT-qPCR |
|  |  | Reverse | TTGGTCCTTAGCCACTCCTTC | RT-qPCR |
| *Tnf* | Mouse | Forward | CCCTCACACTCAGATCATCTTCT | RT-qPCR |
|  |  | Reverse | GCTACGACGTGGGCTACAG | RT-qPCR |
| *Ptgs2* | Mouse | Forward | TGAGCAACTATTCCAAACCAGC | RT-qPCR |
|  |  | Reverse | GCACGTAGTCTTCGATCACTATC | RT-qPCR |
| *NCOA4* | Human | Forward | GCTCAGCAGCTCTACTCGTTA | RT-qPCR |
|  |  | Reverse | GGCACACAGAGACTTGATTGG | RT-qPCR |
| *TRIM3* | Human | Forward | GTCTTCCGTGTTGGCAGTC | RT-qPCR |
|  |  | Reverse | CTGTCTGCTACCACGATGCG | RT-qPCR |
| *CH25H* | Human | Forward | ATCACCACATACGTGGGCTTT | RT-qPCR |
|  |  | Reverse | GTCAGGGTGGATCTTGTAGCG | RT-qPCR |
| *ACTB* | Human | Forward | CATGTACGTTGCTATCCAGGC | RT-qPCR |
|  |  | Reverse | CTCCTTAATGTCACGCACGAT | RT-qPCR |
| *Ch25h* | Mouse | Forward | TCCCAAGGCAGAATCGCTCT | ChIP-qPCR |
|  |  | Reverse | AAATCTCCCCTTCACGCAAGC | ChIP-qPCR |
| *Ncoa4*-KO | Mouse | Wild type | ATGAGATGGATTAGCAGTGAGGG | Genotyping |
|  |  | Common  Mutant | AAGGAAGCCAGAAAAGGAAGTTAG  ACGGGAGGGAGGGAGAAAAT | Genotyping  Genotyping |
| *Ch25h* | Mouse | Sence | ACGACAUGCAUCACUCUCATT | Knockdown |
|  |  | Antisense | UGAGAGUGAUGCAUGUCGUTT | Knockdown |
| *Stat1* | Mouse | Sence | GCTGGACAGTAAAGTCAGA | Knockdown |
|  |  | Antisense | TCTGACTTTACTGTCCAGC | Knockdown |
| *Ncoa4* | Mouse | Sence | GGAGTAUACTCAGAACAAA | Knockdown |
|  |  | Antisense | TTTGTTCTGAGTATACTCC | Knockdown |

**Table S4. Antibodies used in this study.**

| Antibody | Source | Identifier | Application |
| --- | --- | --- | --- |
| β-Actin | Share-Bio | SB-AB0035 | WB |
| NCOA4 | Invitrogen | PA5-96398 | WB, IF |
| NCOA4 | Cell Signaling Technology | 66849 | IP |
| Flag | Sigma | F1804 | WB, IP |
| HA | Cell Signaling Technology | 3724S | WB, IP |
| MYC | Cell Signaling Technology | 2276S | WB, IP |
| MMP2 | Cell Signaling Technology | 87809S | WB |
| MMP9 | Cell Signaling Technology | 24317S | WB |
| TRIM3 | Proteintech | 28392-1-AP | WB |
| GPX4 | Abways | CY6959 | WB |
| FTH1 | Abways | CY5648 | WB |
| SLC7A11 | Abways | CY7046 | WB, IF |
| CH25H | Santa Cruz | sc-293256 | WB, IF |
| NRF2 | Proteintech | 16396-1-AP | WB |
| STAT1 | Proteintech | 10144-2-AP | CUT&Tag, ChIP, WB |
| Phospho-Stat1 (Tyr701) | Cell Signaling Technology | 9167 | WB |
| Phospho-Stat1 (Ser727) | Cell Signaling Technology | 9177 | WB |
| CD68 | Proteintech | 28058-1-AP | IF |
| CD31 | Proteintech | 28083-1-AP | IF |
| α-SMA | Proteintech | 14395-1-AP | IF |

**Table S5: Materials**

| Materials | Company | Cas.No |
| --- | --- | --- |
| 1,2-dilinoleyloxy-3-dimethylaminopropane  (Dlin-MC3-DMA) | AVT Pharmaceutical Tech | O02006 |
| 1,2-distearoyl-sn-glycero-3-phosphocholine  (DSPC) | Lipoid | 556500-2150325-01 |
| 1,2-dimyristoyl-rac-glycero-3-methoxypolyethylene glycol-2000  (DMG-PEG2000) | AVT Pharmaceutical Tech | O02005 |
| 1,2-distearoyl-sn-glycero-3-phosphoethanolamine-N-(polyethylene glycol-2000)-thiol  (DSPE-PEG2000-SH) | Nanosoft Polymers (NSP) | 2350-2000 |
| Ethylenediamine tetraacetic acid  (EDTA) | Sinopharm Chemical Reagent | 10009617 |
| SonoVue® Sulfur Hexafluoride | Bracco | 120312 |
| DMG-PEG2000-MAL | APExBIO | M2005 |

**Table S6: Instruments**

| Instruments | Company | Model |
| --- | --- | --- |
| PARTICLE SIZING SYSTEMS | PSS NICOMP | AccuSizer 780A Autodiluter |
| Dynamic light scattering | Malvern | Zetasizer Lab |
| *In vivo* fluorescence imaging system | Vieworks | VISQUE^®^ InVivo ART 400 |
| All-in-One Fluorescence Microscope | Keyence | BZ-X810 |
| Ultrasound System | Mindray | Resona R9 Pro |
| High-Frequency Ultrasound System | FUJIFILM VisualSonics | *Vevo 2100* |
| Digital slide scanner | Hamamatsu | NanoZoomer S360 |
| Confocal microscope | Nikon | AX R MP |

**Table S7 Complete Blood Count of the three groups in the biosafety evaluation study**

|  | Control  (N = 3) | SI-*Ch25h*-LNP/MB (N = 3) | SI-*Ch25h*-LNP/MB+US (N = 3) | *P*-value |
| --- | --- | --- | --- | --- |
| WBC (10^9/L) | 3.62 ± 0.30 | 4.55 ± 0.01 | 3.51 ± 0.47 | 0.117 |
| Neu# (10^9/L) | 0.39 ± 0.07 | 0.41 ± 0.01 | 0.27 ± 0.08 | 0.318 |
| Lym# (10^9/L) | 3.13 ± 0.22 | 4.00 ± 0.02 | 3.15 ± 0.39 | 0.091 |
| Mon# (10^9/L) | 0.07 ± 0.01 | 0.10 ± 0.03 | 0.05 ± 0.00 | 0.157 |
| Eos# (10^9/L) | 0.04 ± 0.01 | 0.03 ± 0.01 | 0.04 ± 0.00 | 0.236 |
| Bas# (10^9/L) | 0.00 ± 0.00 | 0.01 ± 0.00 | 0.00 ± 0.00 | 0.079 |
| Neu% | 10.47 ± 1.29 | 9.03 ± 0.26 | 7.47 ± 1.12 | 0.186 |
| Lym% | 86.50 ± 1.51 | 87.90 ± 0.55 | 89.83 ± 0.78 | 0.151 |
| Mon% | 1.73 ± 0.15 | 2.10 ± 0.61 | 1.43 ± 0.27 | 0.525 |
| Eos% | 1.20 ± 0.06 | 0.80 ± 0.12 | 1.17 ± 0.13 | 0.070 |
| Bas% | 0.10 ± 0.06 | 0.17 ± 0.09 | 0.10 ± 0.06 | 0.746 |
| RBC (10^12/L) | 7.27 ± 0.20 | 8.18 ± 0.07 | 8.01 ± 0.08 | 0.006 |
| HGB (g/L) | 119.67 ± 4.70 | 132.67 ± 0.33 | 131.67 ± 1.20 | 0.030 |
| HCT (%) | 38.67 ± 3.79 | 44.90 ± 0.42 | 46.60 ± 1.67 | 0.124 |
| MCV (fL) | 53.00 ± 3.70 | 54.87 ± 0.64 | 58.17 ± 2.23 | 0.399 |
| MCH (pg) | 16.47 ± 0.20 | 16.17 ± 0.18 | 16.47 ± 0.23 | 0.422 |
| MCHC (g/L) | 313.33 ± 17.46 | 295.00 ± 2.00 | 283.67 ± 9.17 | 0.258 |
| RDW-CV (%) | 32.53 ± 5.52 | 31.50 ± 0.80 | 24.03 ± 3.00 | 0.276 |
| RDW-SD (fL) | 69.90± 8.05 | 72.13 ± 2.97 | 57.33 ± 4.86 | 0.221 |
| PLT (10^9/L) | 1283.67 ± 97.78 | 1269.67 ± 45.67 | 1424.00 ± 41.00 | 0.270 |
| MPV (fL) | 7.13 ± 0.34 | 6.77 ± 0.07 | 7.50 ± 0.25 | 0.191 |
| PDW (%) | 15.93 ± 0.45 | 15.80 ± 0.25 | 16.13 ± 0.37 | 0.815 |
| PCT (%) | 0.86 ± 0.06 | 0.86 ± 0.04 | 0.93 ± 0.01 | 0.479 |

Data are shown as mean ± SEM and analyzed by one-way ANOVA following LSD post-hoc analysis. Abbreviations: WBC, white blood cell counts; Neu#, neutrophil counts; Lym#, lymphocyte counts; Mon#, monocyte counts; Eos#, eosinophil counts; Bas#, basophil counts; Neu%, neutrophil percentage; Lym%, lymphocyte percentage; Mon%, monocyte percentage; Eos%, eosinophil percentage; Bas%, basophil percentage; RBC, red blood cell counts; HGB, hemoglobin; HCT, hematocrit; MCV, mean corpuscular volume; MCH, mean corpuscular hemoglobin; MCHC, mean corpuscular hemoglobin concentration; RDW-CV, red blood cell volume distribution width-coefficient of variation; RDW-SD, red blood cell volume distribution width-standard deviation; PLT, platelet counts; MPV, mean platelet volume; PDW, platelet distribution width; PCT, plateletcrit.
